# Supplementary figures and images for: Spider phylogenomics: untangling the Spider Tree of Life
Source: PeerJ. 2016 Feb 23;4:e1719. doi: 10.7717/peerj.1719 (PMC4768681; doi:10.7717/peerj.1719)

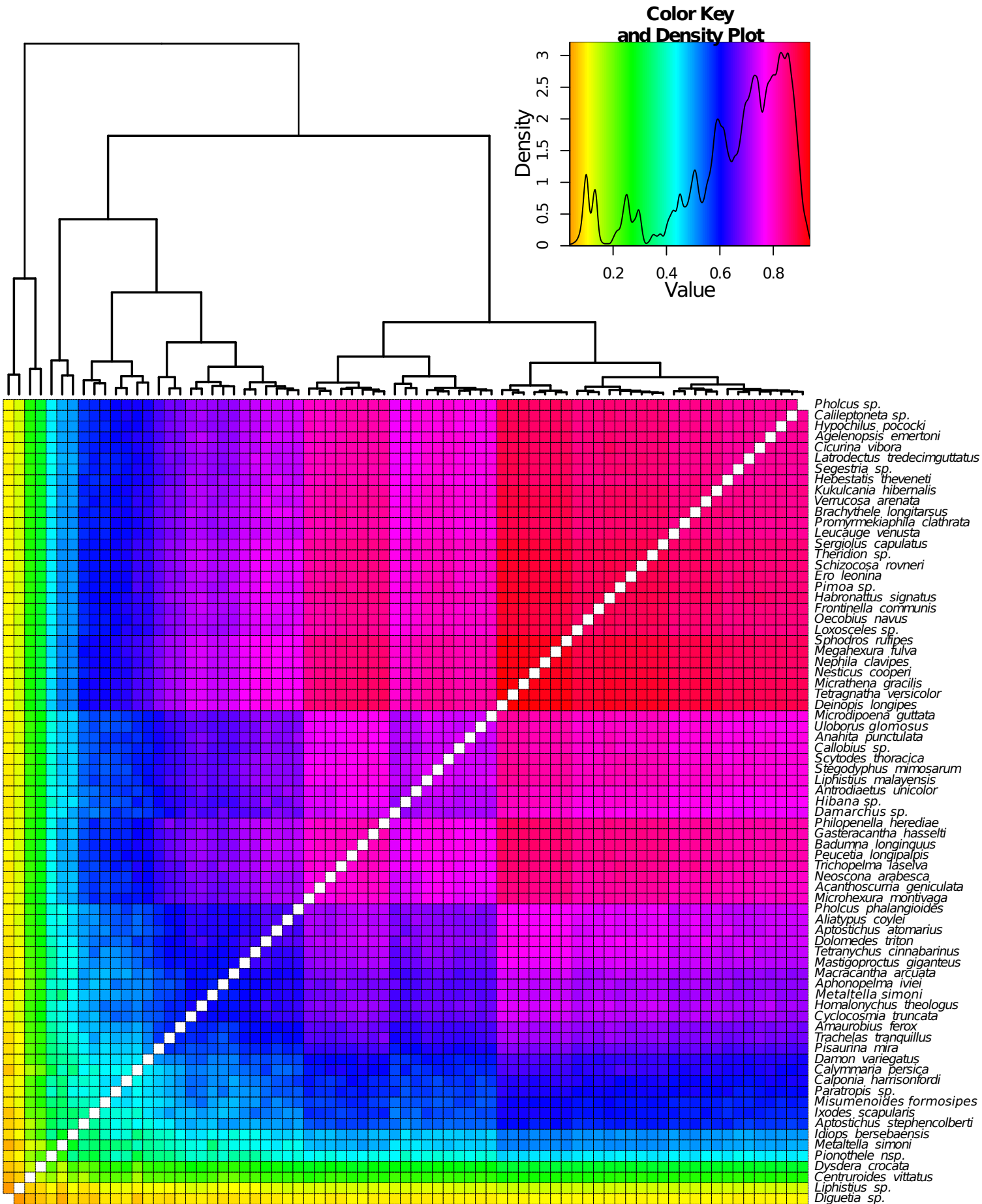

Supplement: Figure S1 — Missing data reduction (matrix 3, Table 2). Degree of positive data overlap indicated by a color-coded heatmap (yellow = low, red = high); species order from right to left in the same order as listed from top to bottom on right side of figure. Lack of phylogenetic clustering indicates bias from shared data does not explain relationships seen in phylogenomic analyses. [file peerj-04-1719-s001.pdf]

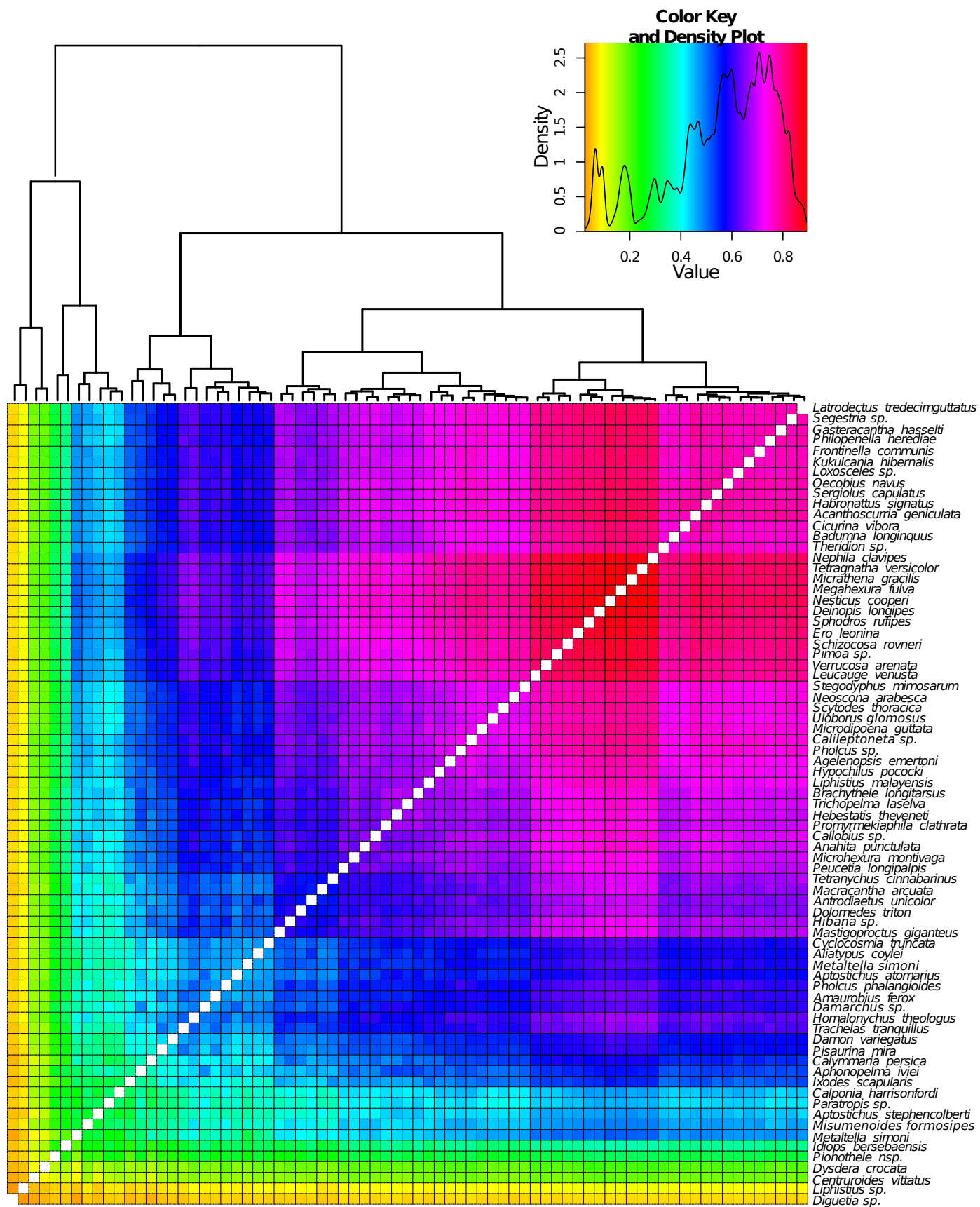

Supplement: Figure S2 — Missing data reduction (matrix 2, Table 2). Degree of positive data overlap indicated by a color-coded heatmap (yellow = low, red = high); species order from right to left in the same order as listed from top to bottom on right side of figure. Lack of phylogenetic clustering indicates bias from shared data does not explain relationships seen in phylogenomic analyses. [file peerj-04-1719-s002.pdf]

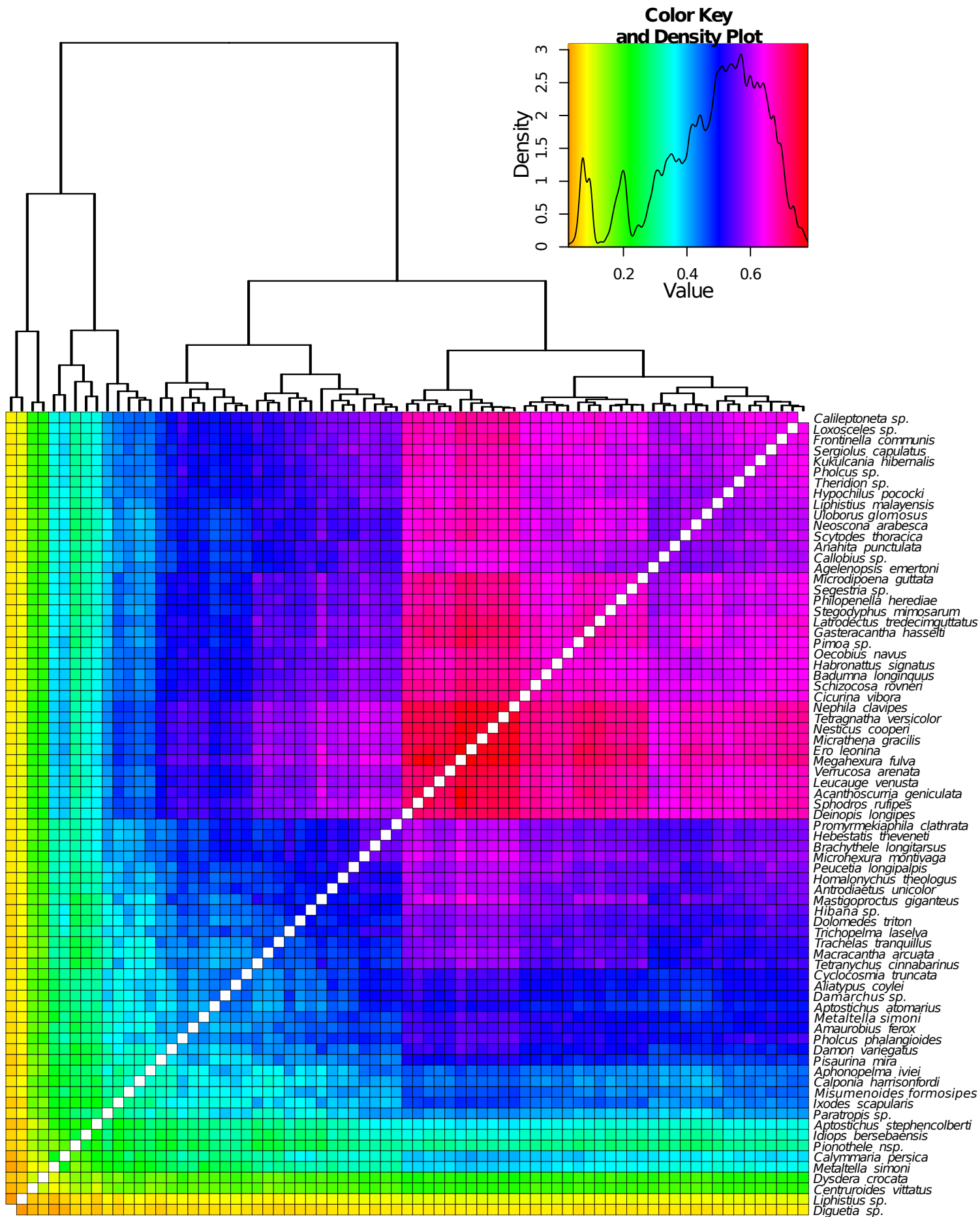

Supplement: Figure S3 — BaCoCa reduced matrix (matrix 4, Table 2). Degree of positive data overlap indicated by a color-coded heatmap (yellow = low, red = high); species order from right to left in the same order as listed from top to bottom on right side of figure. Lack of phylogenetic clustering indicates bias from shared data does not explain relationships seen in phylogenomic analyses. [file peerj-04-1719-s003.pdf]

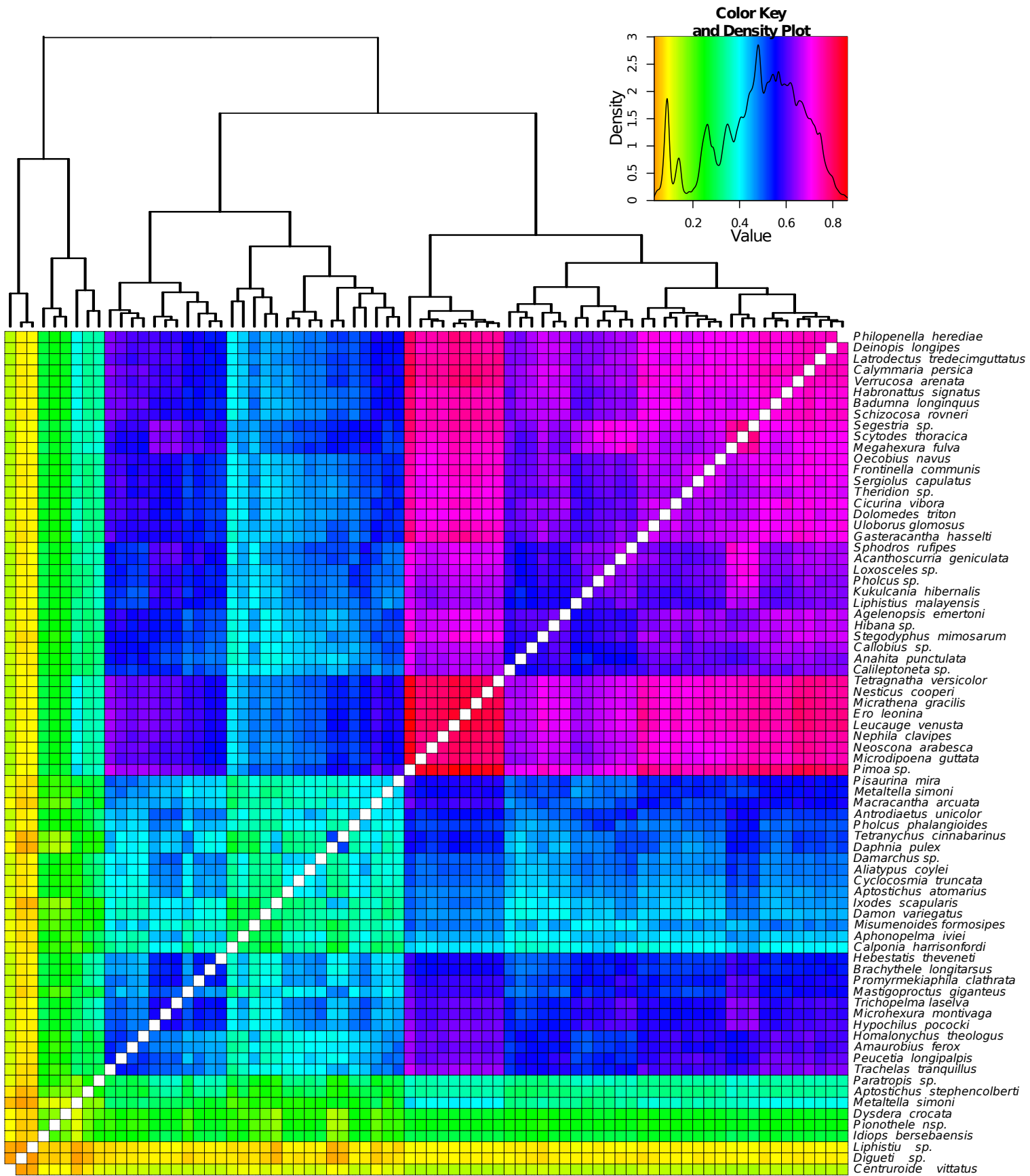

Supplement: Figure S4 — Arthropod core ortholog matrix (matrix 5, Table 2). Degree of positive data overlap indicated by a color-coded heatmap (yellow = low, red = high); species order from right to left in the same order as listed from top to bottom on right side of figure. Lack of phylogenetic clustering indicates bias from shared data does not explain relationships seen in phylogenomic analyses. [file peerj-04-1719-s004.pdf]

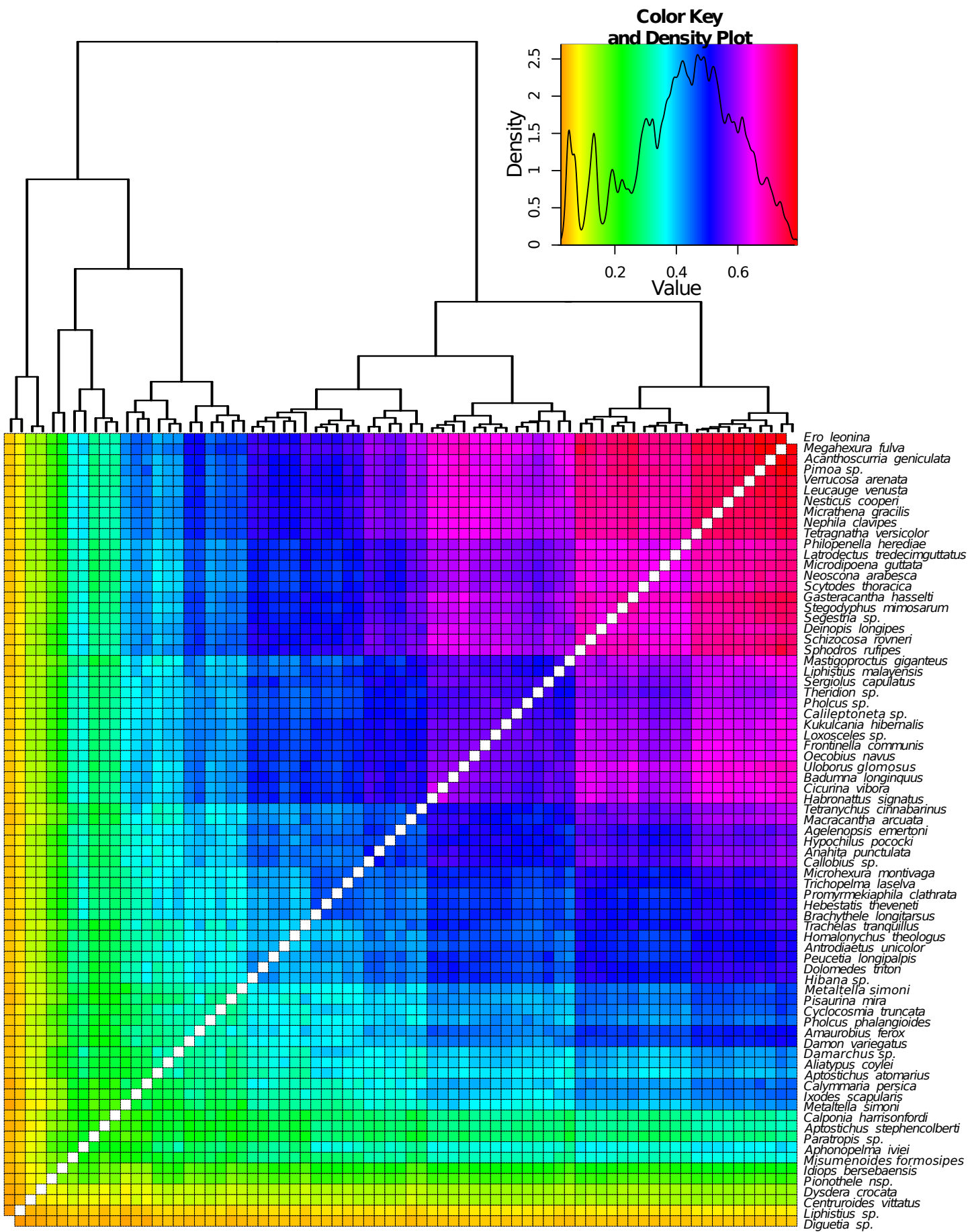

Supplement: Figure S5 — Full spider ortholog matrix (matrix 1, Table 2). Degree of positive data overlap indicated by a color-coded heatmap (yellow = low, red = high); species order from right to left in the same order as listed from top to bottom on right side of figure. Lack of phylogenetic clustering indicates bias from shared data does not explain relationships seen in phylogenomic analyses. [file peerj-04-1719-s005.pdf]

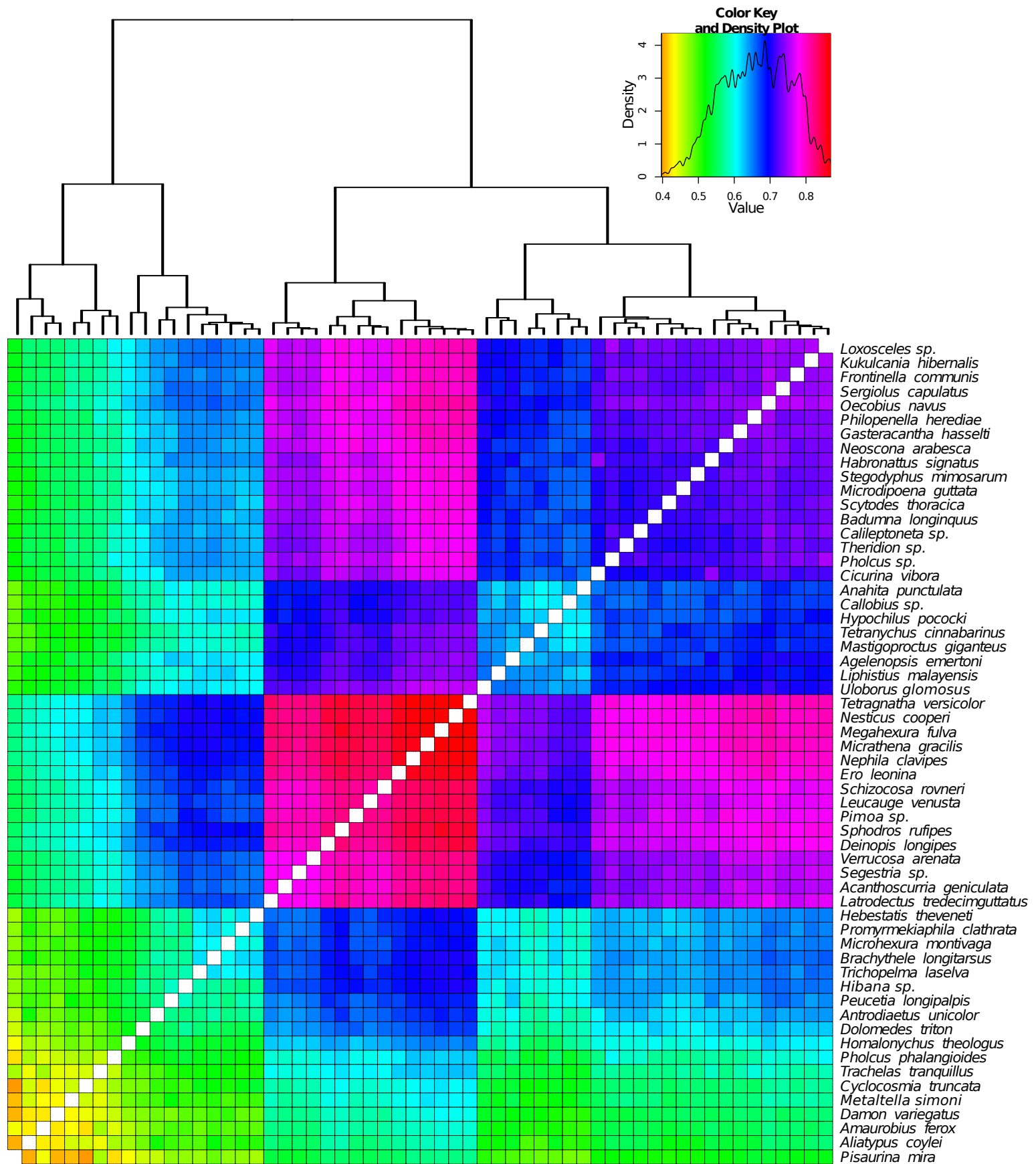

Supplement: Figure S6 — MARE matrix (matrix 7, Table 2). Degree of positive data overlap indicated by a color-coded heatmap (yellow = low, red = high); species order from right to left in the same order as listed from top to bottom on right side of figure. Lack of phylogenetic clustering indicates bias from shared data does not explain relationships seen in phylogenomic analyses. [file peerj-04-1719-s006.pdf]

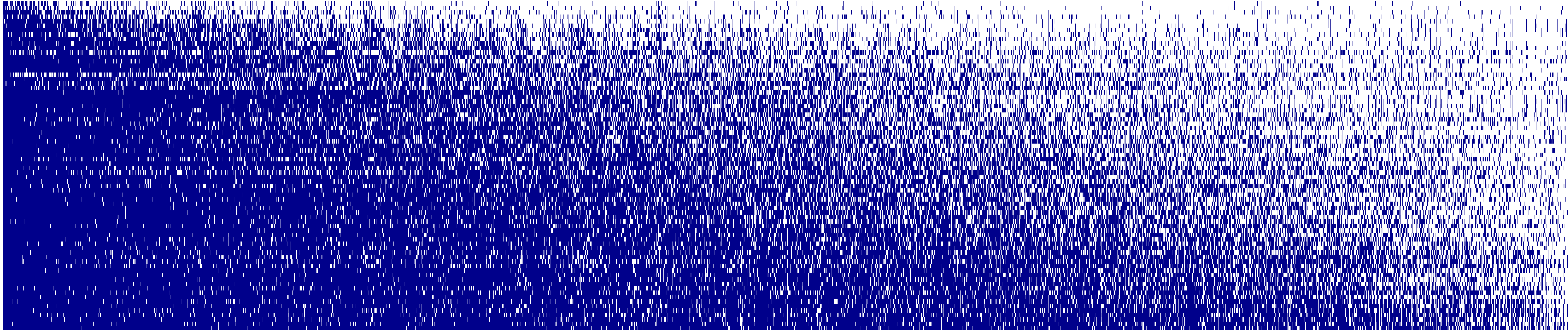

Supplement: Figure S7 — Colored squares represent partitions present in matrix for each OTU (x-axis, in descending order of OTU representation from left to right) and each partition or gene (y-axis, in ascending order of partition representation). [file peerj-04-1719-s007.pdf]

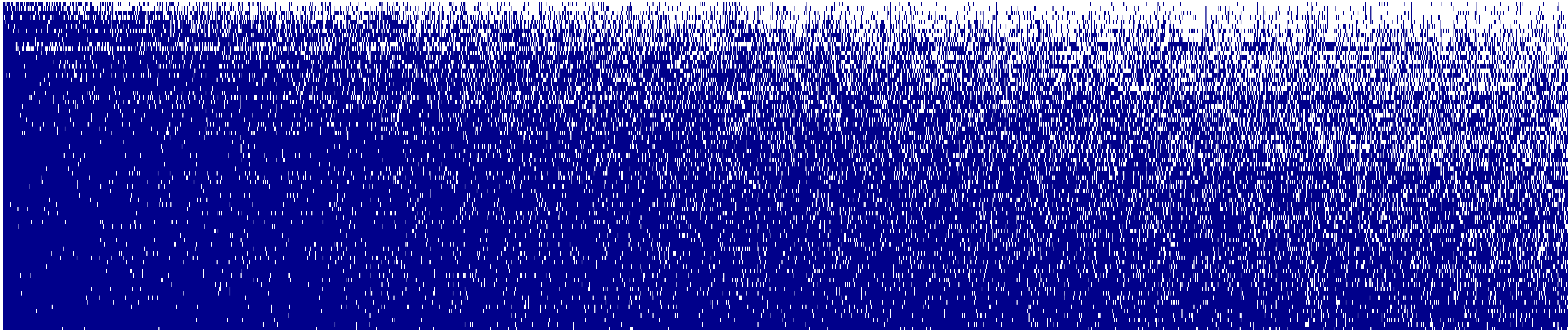

Supplement: Figure S8 — Colored squares represent partitions present in matrix for each OTU (x-axis, in descending order of OTU representation from left to right) and each partition or gene (y-axis, in ascending order of partition representation). Figure [file peerj-04-1719-s008.pdf]

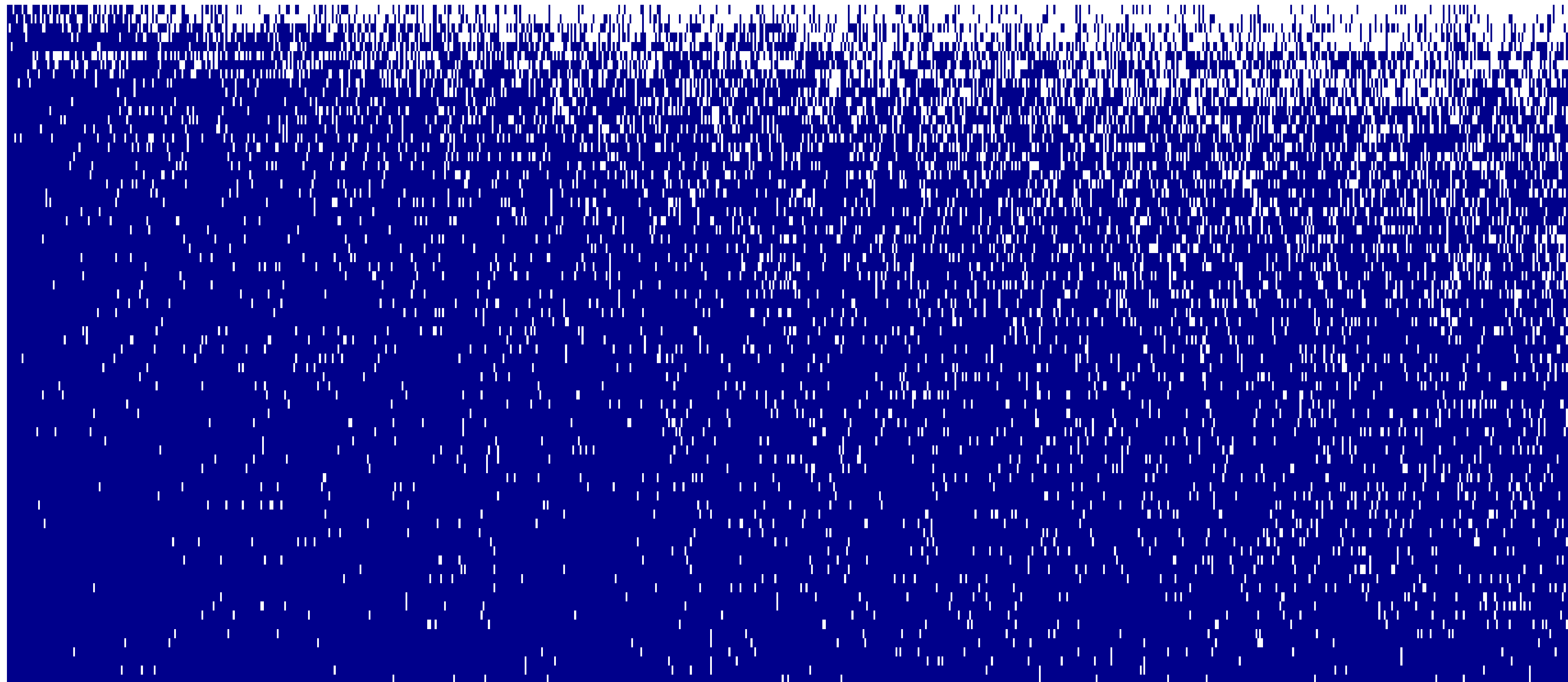

Supplement: Figure S9 — Colored squares represent partitions present in matrix for each OTU (y-axis, in descending order of OTU representation from bottom to top) and each partition or gene (x-axis, in descending order of partition representation from left to right). [file peerj-04-1719-s009.pdf]

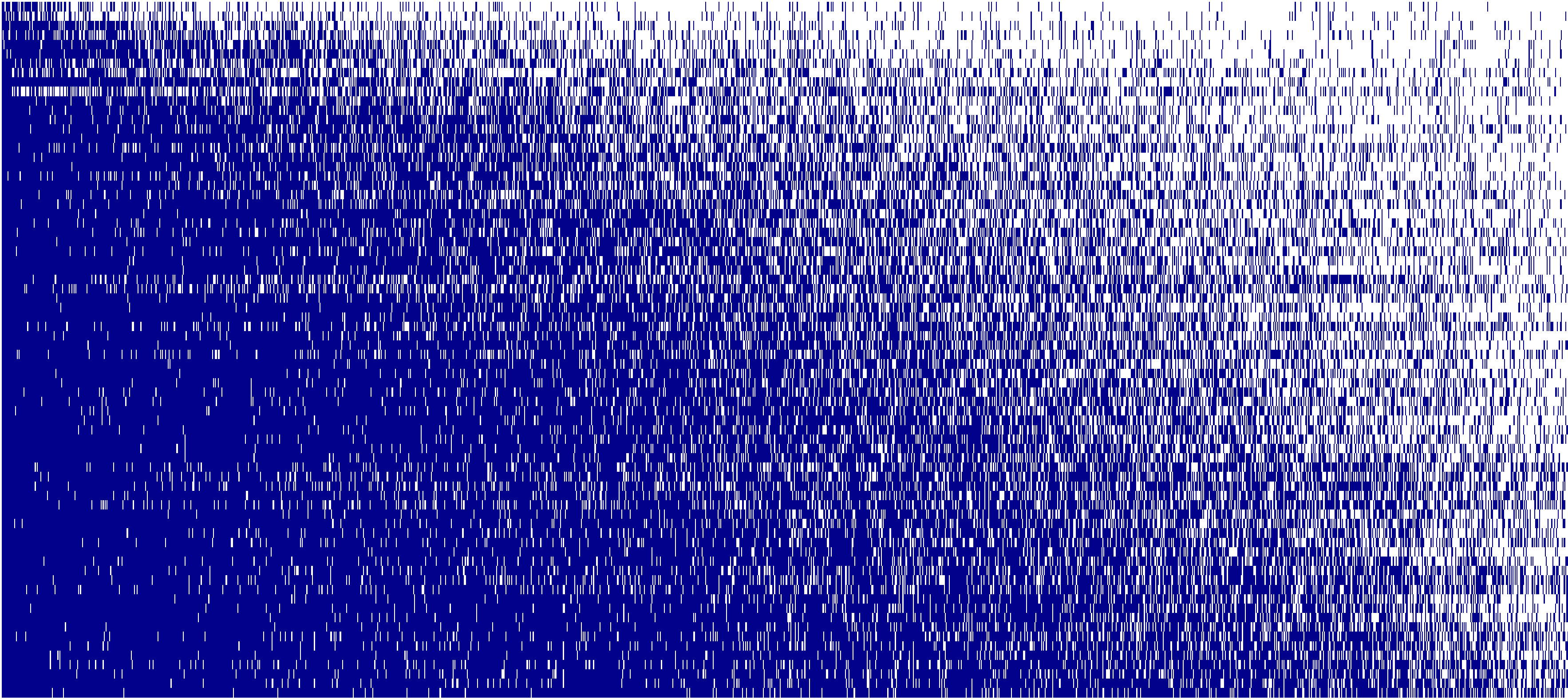

Supplement: Figure S10 — Colored squares represent partitions present in matrix for each OTU (y-axis, in descending order of OTU representation from bottom to top) and each partition or gene (x-axis, in descending order of partition representation from left to right). [file peerj-04-1719-s010.pdf]

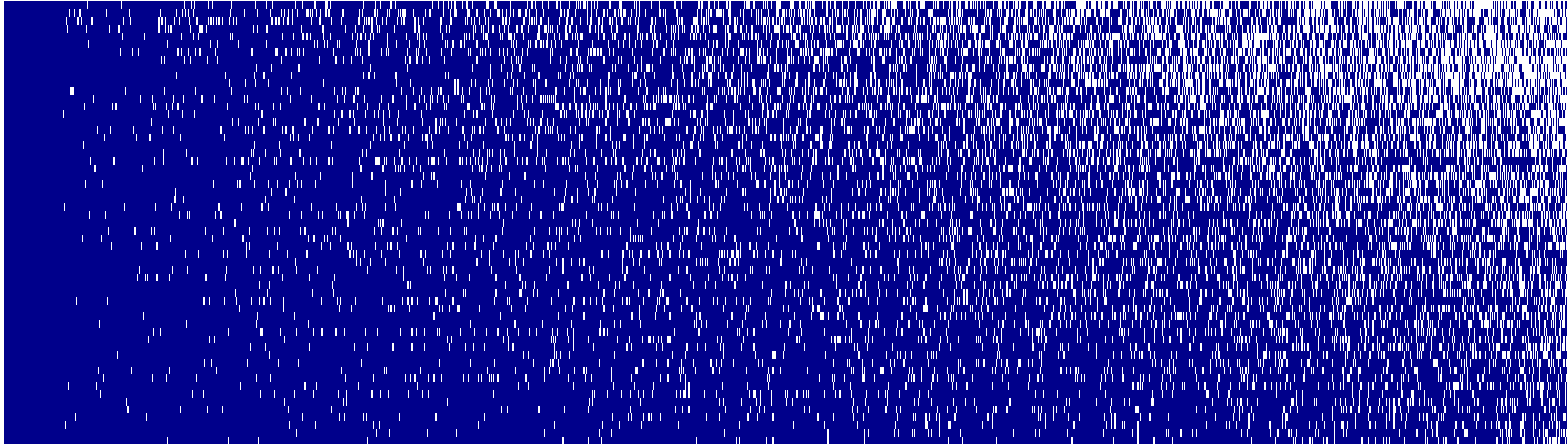

Supplement: Figure S11 — Colored squares represent partitions present in matrix for each OTU (y-axis, in descending order of OTU representation from bottom to top) and each partition or gene (x-axis, in descending order of partition representation from left to right). [file peerj-04-1719-s011.pdf]

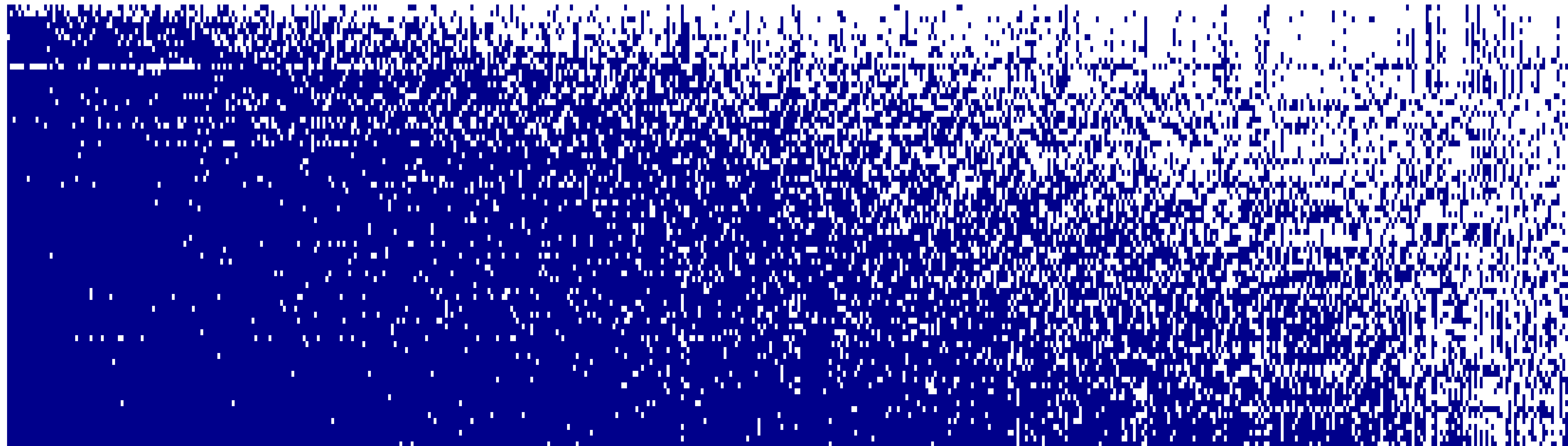

Supplement: Figure S12 — Colored squares represent partitions present in matrix for each OTU (y-axis, in descending order of OTU representation from bottom to top) and each partition or gene (x-axis, in descending order of partition representation from left to right). [file peerj-04-1719-s012.pdf]

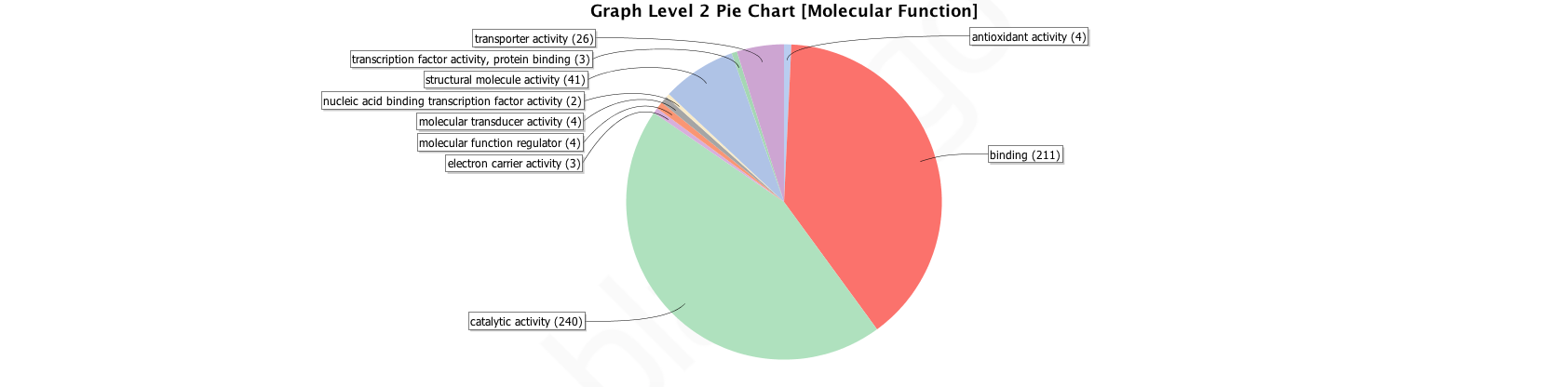

Supplement: Figure S13 — Gene Ontology molecular functions, levels 2 for OGs shared by Arthropod and Spider Core sets. Figures generated by Blast2GO analysis. [file peerj-04-1719-s013.png]

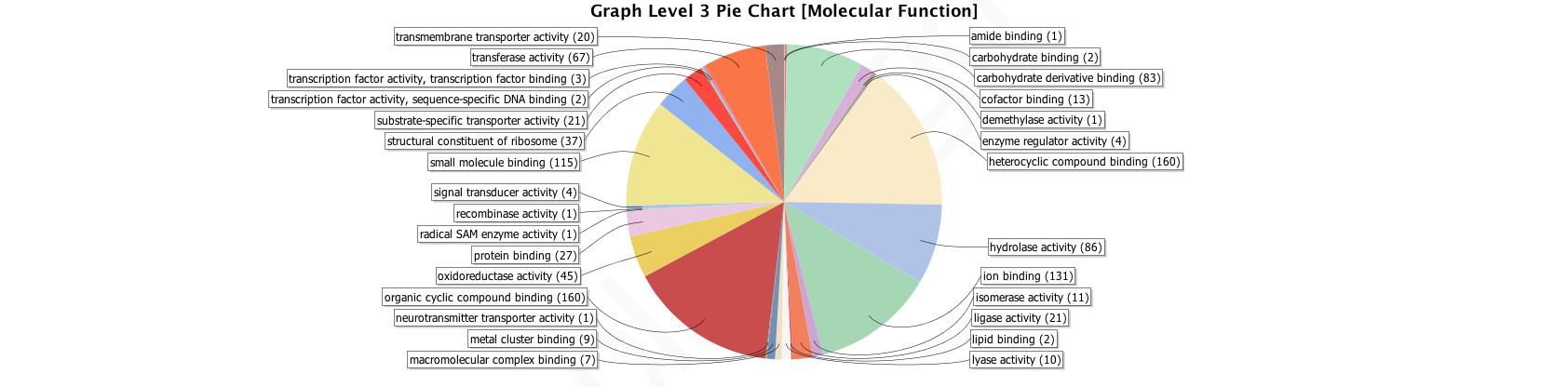

Supplement: Figure S14 — Gene Ontology molecular functions, level 3 for OGs shared by Arthropod and Spider Core sets. Figures generated by Blast2GO analysis. [file peerj-04-1719-s014.png]
