# Supplementary material for: Spider phylogenomics: untangling the Spider Tree of Life
Source: PeerJ. 2016 Feb 23;4:e1719. doi: 10.7717/peerj.1719 (PMC4768681; doi:10.7717/peerj.1719)
Supplement: Supplemental Information 4 [file peerj-04-1719-s018.pdf]

SUPPLEMENTAL MATERIALS

Table 1. Specimen locality data for newly sequenced transcriptomes.

Family	Genus	Specific Epithet	Specimen ID	Locality	Latitude/Longitude
Agelenidae	*Agelenopsis*	*emertoni*	AUMS5739	Auburn, Lee Co, AL, USA	32.6028°N 85.4554°W
Amaurobiidae	*Callobius*	*sp.*	AUMS11930	Muir Woods, Marin Co, CA, USA	37.8922°N 122.5717°W
Amphinectidae	*Metaltella*	*simoni*	AUMS11905	Auburn University, Lee Co, AL, USA	32.5986°N 85.4833°W
Amphinectidae	*Metaltella*	*simoni*	AUMS11915	SanDiego Co, CA, USA	NA
Antrodiaetidae	*Aliatypus*	*coylei*	AUMS56	near Monterey, Monterey Co, CA, USA	36.5714°N 121.9043°W
Antrodiaetidae	*Antrodiaetus*	*unicolor*	MY2335	near Hendersonville, Transylvania Co, NC, USA	35.2192°N 82.7794°W
Anyphaenidae	*Hibana*	*sp.*	AUMS11902	Louise Kreher Forest Ecology Preserve, Auburn, Lee Co, AL, USA	32.6603°W 85.4836°W
Araneidae	*Micrathena*	*gracilis*	AUMS5620	Dadeville, Co, AL, USA	32.8318°N 85.7636°W
Araneidae	*Verrucosa*	*arenata*	AUMS11901	Louise Kreher Forest Ecology Preserve, Auburn, Lee Co, AL, USA	32.6603°N 85.4836°W
Atypidae	*Sphodros*	*rufipes*	AUMS146	EV Smith Research Station, Macon Co, AL, USA	32.4257°N 85.9015°W
Barychelidae	*Trichopelma*	*laselva*	AUMS8485	La Selva, CR	10.4295°N 84.0095°W

Supplemental Table 1 – continued from previous page

Family	Genus	Specific Epithet	Specimen ID	Locality	Latitude/Longitude
Caponiidae	*Calponia*	*harrisonfordi*	1347-JML-001	Russian Ridge Open Space Park, San Mateo Co, CA, USA	37.3149°N 122.1872°W
Ctenidae	*Anahita*	*punctulata*	AUMS11932	Auburn, Lee Co, AL, USA	32.6028°N 85.4554°W
Ctenizidae	*Cyclocosmia*	*truncata*	AUMS120	Grove Hill, Auburn, Lee Co, AL, USA	32.5841°N 85.4604°W
Ctenizidae	*Hebestatis*	*theveneti*	AUMS152	Tuolumne Co, CA, USA	38.0453°N 119.9747°W
Deinopidae	*Deinopis*	*longipes*	AUMS8783	La Selva, CR	10.4295°N 84.0095°W
Desidae	*Badumna*	*longinqua*	AUMS11914	Petaluma, Sonoma Co, CA, USA	38.2247°N 122.6264°W
Dictynidae	*Cicurina*	*vibora*	HED003	Temples of Thor Cave, Williamson Co, TX, USA	NA
Diguetidae	*Diguetia*	*sp.*	AUMS11919	Iperial Co, CA, USA	NA
Dipluridae	*Microhexura*	*montivaga*	HED001	Mt Gibbes, Mt Mitchell SP, Yancey Co, NC, USA	35.7394°N 82.2850°W
Dysderidae	*Dysdera*	*crocata*	AUMS11924	Golden Gate Park, San Francisco Co, CA, USA	37.7686°N 122.4659°W
Euctenizidae	*Aptostichus*	*atomarius*	MY4002	Winchester, Riverside Co, CA, USA	33.7069°N 117.0844°W
Euctenizidae	*Aptostichus*	*stephencolberti*	AUMS20	Marina Dunes State Park, Monterey Co, CA, USA	36.7035°N 121.8068°W

Supplemental Table 1 – continued from previous page

Family	Genus	Specific Epithet	Specimen ID	Locality	Latitude/Longitude
Euctenizidae	*Promyrmekiaphila*	*clathrata*	AUMS5761	near Cazadero, Sonoma Co, CA, USA	38.5231°N 123.0845°W
Filistatidae	*Kukulcania*	*hibernalis*	AUMS8597	Auburn, Lee Co, AL, USA	32.6094°N 85.4544°W
Gnaphosidae	*Sergiolus*	*capulatus*	AUMS5674	Opelika, Lee Co, AL, USA	32.6887°N 85.4012°W
Hahniidae	*Calymmaria*	*persica*	AUMS11926	Petaluma, Sonoma Co, CA, USA	38.2247°N 122.6264°W
Homalonychidae	*Homalonychus*	*theologus*	AUMS11917	Imperial Co, CA, USA	NA
Hypochilidae	*Hypochilus*	*pococki*	AUMS155	Laurel Falls Trail, Sevier Co, TN, USA	35.6782°N 83.5929°W
Idiopidae	*Idiops*	*bersebaensis*	AUMS6746	Namibia, Africa	17.0021°S 13.2445°E
Leptonetidae	*Calileptoneta*	*californica*	1348-JML-001	Mount Diablo State Park, Contra Co, CA, USA	37.8745°N 121.9616°W
Linyphiidae	*Frontinella*	*communis*	AUMS5733	Auburn University, Lee Co, AL, USA	32.5987°N 85.4835°W
Liphistiidae	*Liphistius*	*sp.*	AUMS5743	Thailand	NA
Lycosidae	*Schizocosa*	*rovneri*	AUMS5122	Oxford, Lafayette Co, MS, USA	32.4116°N 89.4183°W
Mecicobothriidae	*Megahexura*	*fulva*	AUMS154	East of Groveland, Tuolumne Co, CA, USA	38.0453°N 119.9747°W
Mimetidae	*Ero*	*leonina*	AUMS8817	Tuskegee National Forest, Macon Co, AL, USA	32.4418°N 85.6328°W
Nemesiidae	*Calisoga*	*longitarsis*	AUMS47	Jasper Ridge, San Mateo Co, CA, USA	37.4132°N 122.2050°W

Supplemental Table 1 – continued from previous page

Family	Genus	Specific Epithet	Specimen ID	Locality	Latitude/Longitude
Nemesiidae	*Damarchus*	*sp.*	AUMS5127	Ramnagar Nepal	NA
Nemesiidae	*Pionothele*	*n.sp.*	AUMS6718	Namibia, Africa	23.5698°S 15.0398°E
Nephilidae	*Nephila*	*clavipes*	AUMS5736	Donald E. Davis Arboretum, Auburn University, Lee Co, AL, USA	32.5950°N 85.4834°W
Nesticidae	*Nesticus*	*cooperi*	HED002	Nantahala River Gorge, Swain Co, NC, USA	35.4111°N 83.5220°W
Oecobiidae	*Oecobius*	*navus*	AUMS5741	Auburn University, Lee Co, AL, USA	32.6000°N 85.4823°W
Oxyopidae	*Peucetia*	*longipalpis*	AUMS5740	Opelika, Lee Co, AL, USA	32.6887°N 85.4012°W
Paratropididae	*Paratropis*	*sp.*	AUMS148	Socotá, Departamento Boyaca, Columbia	6.08976°N 72.6198°W
Pholcidae	*Pholcus*	*phalangioides*	AUMS5732	Auburn University, Lee Co, AL, USA	32.6000°N 85.4823°W
Pholcidae	*Pholcus*	*phalangioides*	1304-JML-001	Petaluma, Sonoma Co, CA, USA	38.2247°N 122.6264°W
Pimoidae	*Pimoa*	*sp.*	AUMS14951	Del Norte Co, CA	NA
Pisauridae	*Dolomedes*	*triton*	AUMS11906	Opelika, Lee Co, AL, USA	32.6544°N 85.3973°W
Salticidae	*Habronattus*	*signatus*	HED004	Ocotillo, Imperial Co, CA, USA	32.7386°N 115.9941°W
Scytodidae	*Scytodes*	*thoracica*	AUMS5673	Grove Hill, Lee Co, AL, USA	32.5841°N 85.4604°W
Segestriidae	*Segestria*	*sp.*	AUMS11925	Golden Gate Park, San Francisco, CA, USA	37.7686°N 122.4659°W

Supplemental Table 1 – continued from previous page

Family	Genus	Specific Epithet	Specimen ID	Locality	Latitude/Longitude
Sicariidae	*Loxosceles*	*deserta*	1346-JML-001	Yarnell, Yavapai Co, AZ	32.0756°N 110.6258°W
Tetragnathidae	*Leucauge*	*venusta*	AUMS11903	Chewacla State Park, Auburn, Lee Co, AL, USA	32.5536°N 85.4845°W
Tetragnathidae	*Tetragnatha*	*versicolor*	AUMS5738	Donald E. Davis Arboretum, Auburn University, Lee Co, AL, USA	32.5950°N 85.4834°W
Theraphosidae	*Aphonopelma*	*iviei*	APH2038	East of Plymouth, Amador Co, CA, USA	38.4759°N 120.8230°W
Theridiidae	*Theridion*	*sp.*	AUMS5737	Donald E. Davis Arboretum, Auburn University, AL, USA	32.5950°N 85.4834°W
Thomisidae	*Misumenoides*	*formosipes*	AUMS6454	Opelika, Lee Co, AL, USA	32.6887°N 85.4012°W
Uloboridae	*Philopenella*	*herediae*	AUMS8784	La Selva, CR	10.4295°N 84.0095°W
Uloboridae	*Uloborus*	*glomosus*	AUMS11904	Chewacla State Park, Auburn, Lee Co, AL, USA	32.5536°N 85.4845°W

Table 2. Transcriptome sequence and assembly data.

Family	Species	Reads	Contigs	Ave. Len.	TransDecoder	ARTH	SPID
Agelenidae	*Agelenopsis emertoni*	27264400	20517	719.7	8192	751	2613
Amaurobiidae	*Amaurobius ferox*	12549070	202311	530.8	35361	976	3247
Amaurobiidae	*Callobius sp.*	26240373	62055	499	21591	919	3401
Amphinectidae	*Metaltella simoni*	14728368	42301	574.7	18716	889	3039
Amphinectidae	*Metaltella simoni*	12935049	62734	612.4	23086	950	3174
Antrodiaetidae	*Aliatypus coylei*	29958173	23447	744.6	6098	695	2222
Antrodiaetidae	*Antrodiaetus unicolor*	32624239	14062	550.2	9850	756	2745
Anyphaenidae	*Hibana sp.*	40346328	55291	615.1	15212	909	2789
Araneidae	*Verrucosa arenata*	14468378	94721	911.9	41450	976	3993
Araneidae	*Gasteracantha hasselti*	12564452	50554	858.2	19944	978	4020
Araneidae	*Macracantha arcuata*	17523883	34411	787.9	16560	949	3826
Araneidae	*Neoscona arabesca*	28551664	161311	745.6	47096	992	3834
Araneidae	*Micrathena gracilis*	56963267	38210	856.6	16269	935	3719
Atypidae	*Sphodros rufipes*	51968533	27266	715.5	11504	915	3704
Barychelidae	*Trichopelma laselva*	27264400	33544	665.4	9061	807	2861
Buthidae	*Centruroides vittatus*	45691843	17788	378.2	4854	660	1099
Caponiidae	*Calponia harrisonfordi*	59921781	14397	542.1	4297	551	1604
Ctenidae	*Anahita punctulata*	44538794	75653	483.4	23111	961	3407
Ctenizidae	*Cyclocosmia truncata*	33664901	26408	604.7	8446	736	2679
Ctenizidae	*Hebestatis theveneti*	40097804	16167	650	6711	713	2647
Deinopidae	*Deinopis longipes*	39222056	36976	705.7	12764	880	3375
Desidae	*Badumna longinqua*	16455974	85746	661.9	34737	968	3557
Dictynidae	*Cicurina vibora*	29071083	175943	412.7	20256	852	3293
Diguetidae	*Diguetia sp.*	19838746	6816	306.9	1368	309	457
Dipluridae	*Microhexura montivaga*	24680385	19680	635.8	8286	761	2695

Supplemental Table 2 – continued from previous page

Family	Species	Reads	Contigs	Ave. Len.	TransDecoder	ARTH	SPID
Dysderidae	*Dysdera crocata*	1718572	18286	452.8	5058	566	932
Eresidae	*Stegodyphus mimosarum*	NA	NA	NA	26888	965	4055
Euctenizidae	*Aptostichus atomarius*	27431535	14152	709.2	5795	643	2149
Euctenizidae	*Aptostichus stephencolberti*	30904990	13267	779.5	5344	589	1944
Euctenizidae	*Promyrmekiaphila clathrata*	24733435	22423	669.4	8445	758	2646
Filistatidae	*Kukulcania hibernalis*	42693292	29491	702.4	11121	835	3141
Gnaphosidae	*Sergiolus capulatus*	32765239	28757	725.6	11340	820	2989
Hahniidae	*Calymmaria persica*	19286137	110707	644.9	20501	982	2165
Homalonychidae	*Homalonychus theologus*	31165362	40734	462.1	13258	933	3167
Hypochilidae	*Hypochilus pococki*	25747925	19793	540.2	7561	796	2860
Idiopidae	*Idiops bersebaensis*	23040778	6270	649.1	2897	421	1151
Ixodidae	*Ixodes scapularis*	NA	18810	668.4	17799	815	2726
Leptonetidae	*Calileptoneta californica*	59806212	37641	582	11687	875	3240
Linyphiidae	*Frontinella communis*	28476743	27195	757.1	11753	862	3126
Liphistiidae	*Liphistius sp.*	54043289	7830	370.5	1938	333	667
Liphistiidae	*Liphistius malayanus*	62897982	83669	515.4	19784	941	3568
Lycosidae	*Schizocosa rovneri*	132349831	42744	871.6	14965	911	3584
Mecicobothriidae	*Megahexura fulva*	59599533	40526	671.9	15303	964	4147
Mimetidae	*Ero leonina*	32363996	78785	801.7	24565	980	3971
Mysmenidae	*Microdipoena guttata*	16972695	156439	617.5	70785	998	3940
Nemesiidae	*Calisoga longitarsis*	30773715	20721	574.8	7674	743	2635
Nemesiidae	*Damarchus sp.*	21876221	13519	707.8	5558	623	2089
Nemesiidae	*Pionothele n.sp.*	20155275	5158	631.7	2283	368	970
Nephilidae	*Nephila clavipes*	34853551	32305	753.9	15200	935	3653
Nesticidae	*Nesticus cooperi*	20188741	41169	714	15744	945	3715

Supplemental Table 2 – continued from previous page

Family	Species	Reads	Contigs	Ave. Len.	TransDecoder	ARTH	SPID
Oecobiidae	*Oecobius navus*	25031200	24989	695.4	12582	881	3183
Oxyopidae	*Peucetia longipalpis*	23273514	18810	668.4	7994	737	2536
Paratropididae	*Paratropis sp.*	18409810	9021	605	3694	473	1463
Pholcidae	*Pholcus phalangioides*	24861584	17870	556.9	6698	703	3183
Pholcidae	*Pholcus phalangioides*	58019035	40396	760.8	11883	902	2129
Phrynichidae	*Damon variegatus*	64733221	83669	515.4	27304	944	3327
Pimoidae	*Pimoa sp.*	46352103	160889	978.3	47496	998	3915
Pisauridae	*Dolomedes triton*	13590617	81075	625.3	32520	951	3209
Pisauridae	*Pisaurina mira*	6887470	129530	563.8	27116	958	3219
Salticidae	*Habronattus signatus*	75391275	26276	574.9	10447	843	3259
Scytodidae	*Scytodes thoracica*	30924460	59599	855.7	18223	962	3657
Segestriidae	*Segestria sp.*	38407502	191839	708.3	54753	987	3804
Sicariidae	*Loxosceles deserta*	61963685	40702	582.5	11078	876	3256
Tetragnathidae	*Leucauge venusta*	15567091	127727	919	51004	988	3972
Tetragnathidae	*Tetragnatha versicolor*	33465090	34328	815.4	15917	938	3672
Tetranychidae	*Tetranychus cinnabarinus*	26040173	30288	963.8	17083	938	3063
Thelyphonidae	*Mastigoproctus giganteus*	25983006	157263	623.7	43626	994	3785
Theraphosidae	*Acanthoscurria geniculata*	NA	NA	NA	76237	933	4249
Theraphosidae	*Aphonopelma iviei*	36326210	13442	605.1	4717	596	2035
Theridiidae	*Latrodectus tredecimguttatus*	27605467	10259	1110.6	9266	904	3399
Theridiidae	*Theridion sp.*	37459365	24669	733.7	10013	800	2810
Thomisidae	*Misumenoides formosipes*	25351927	58396	570.4	21546	810	2161
Trachelidae	*Trachelas tranquillus*	15506968	118533	467.9	30037	950	3546
Uloboridae	*Philoponella herediae*	47234871	40659	655.9	16468	947	3656
Uloboridae	*Uloborus glomosus*	12362545	114137	657	40782	970	3810

4

Supplemental Table 3

See /Supplemental_Material/AnnotationTable_S3.tex :too large to compile within main.tex

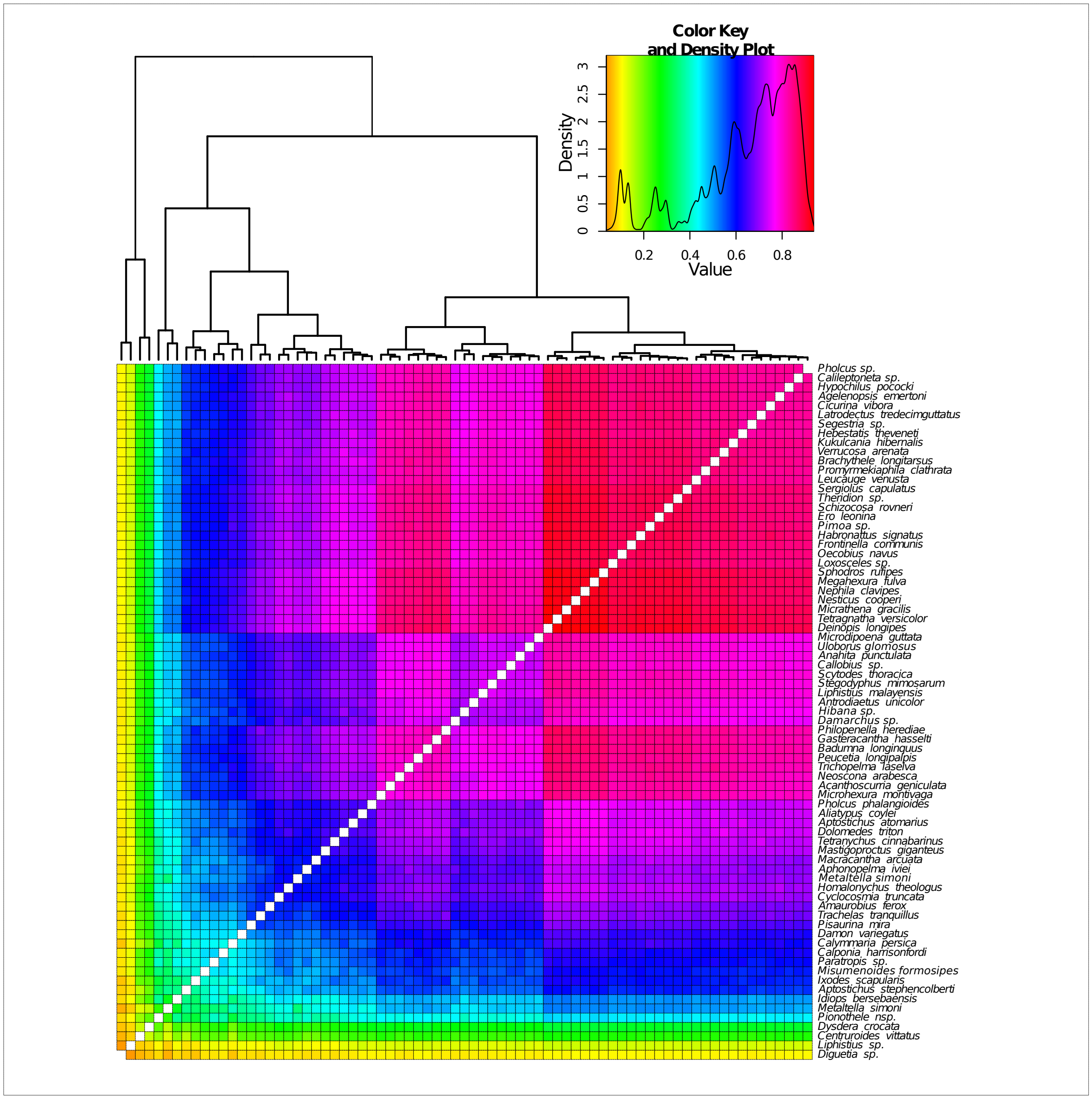

Figure 1. Clustering/heatmap analysis depicting degree of shared data between each species pair; missing data reduction (matrix 3, Table 2). Degree of positive data overlap indicated by a color-coded heatmap (yellow = low, red = high); species order from right to left in the same order as listed from top to bottom on right side of figure. Lack of phylogenetic clustering indicates bias from shared data does not explain relationships seen in phylogenomic analyses.

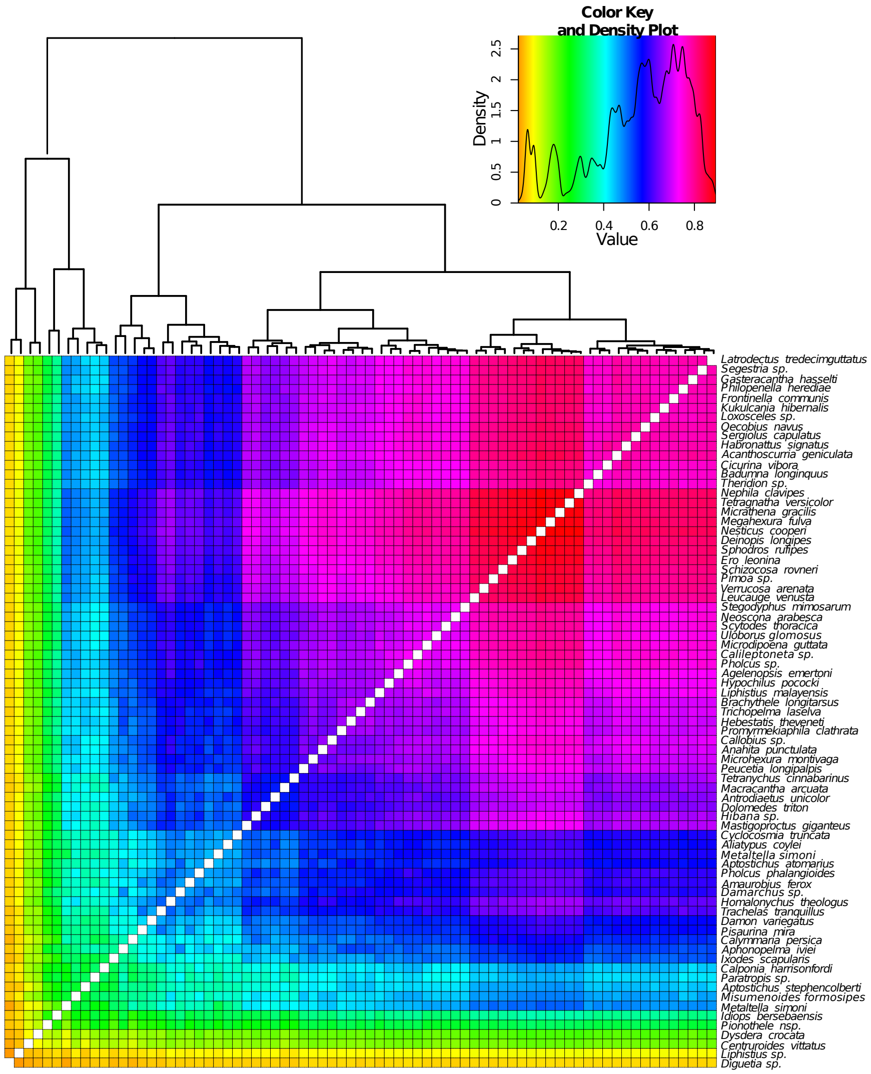

Figure 2. Clustering/heatmap analysis depicting degree of shared data between each species pair; missing data reduction (matrix 2, Table 2). Degree of positive data overlap indicated by a color-coded heatmap (yellow = low, red = high); species order from right to left in the same order as listed from top to bottom on right side of figure. Lack of phylogenetic clustering indicates bias from shared data does not explain relationships seen in phylogenomic analyses.

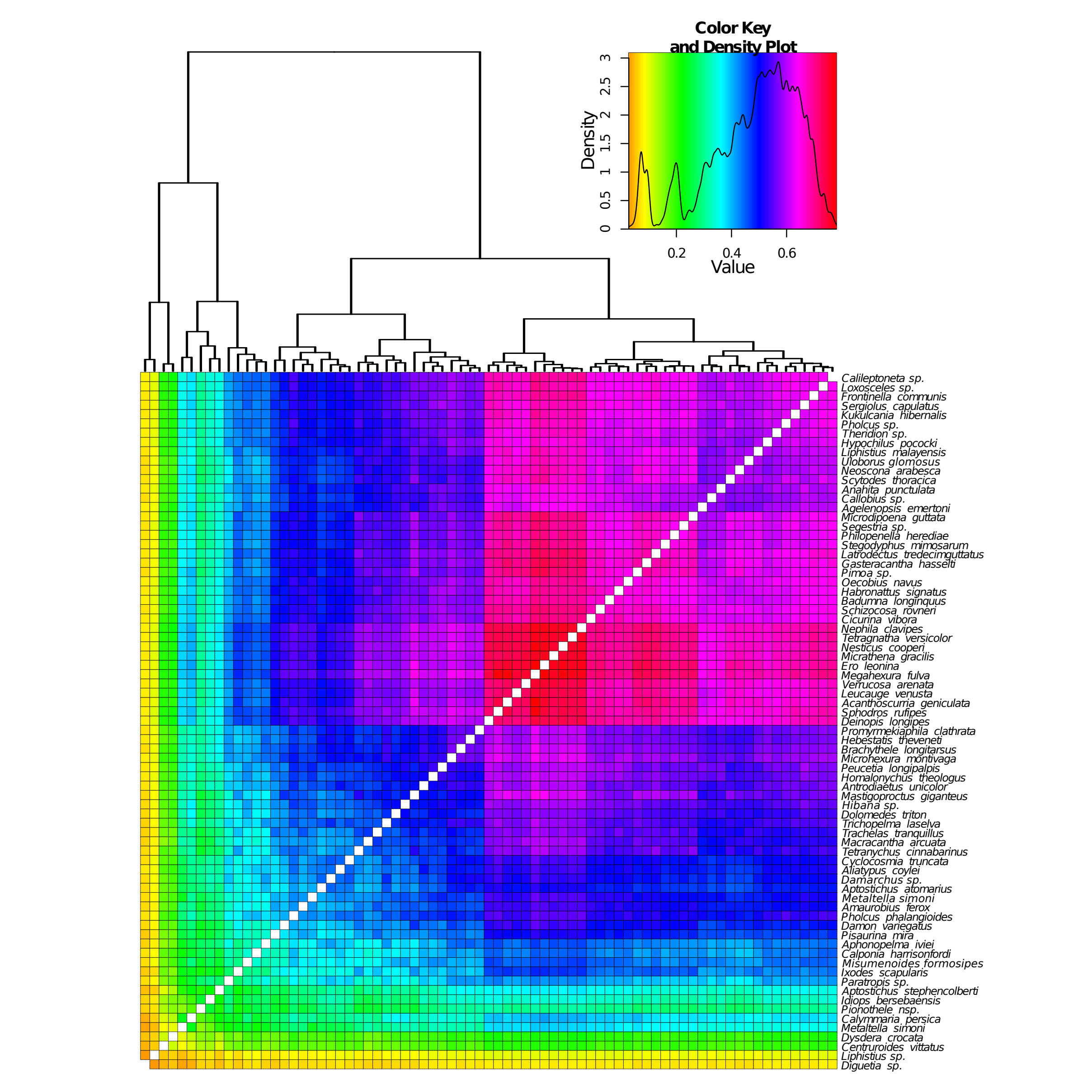

Figure 3. Clustering/heatmap analysis depicting degree of shared data between each species pair; BaCoCa reduced matrix (matrix 4, Table 2). Degree of positive data overlap indicated by a color-coded heatmap (yellow = low, red = high); species order from right to left in the same order as listed from top to bottom on right side of figure. Lack of phylogenetic clustering indicates bias from shared data does not explain relationships seen in phylogenomic analyses.

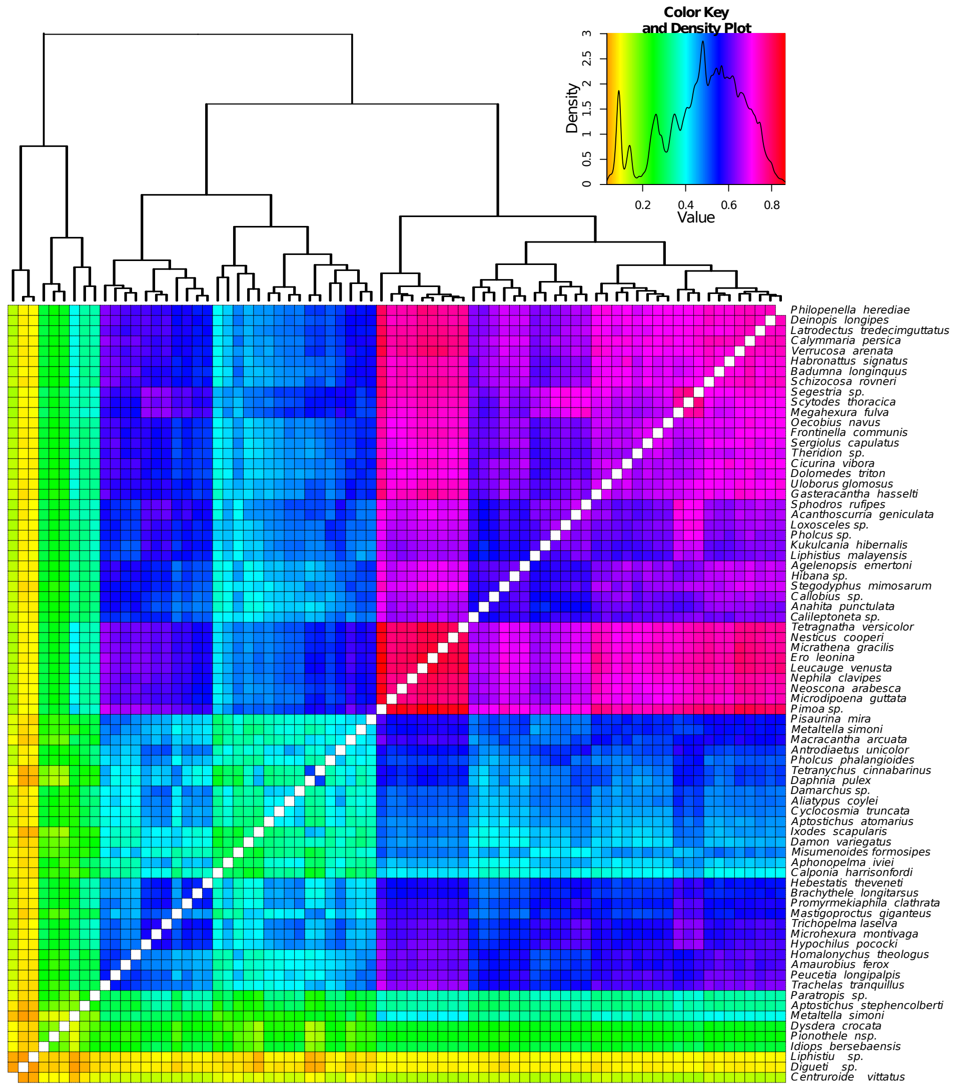

Figure 4. Clustering/heatmap analysis depicting degree of shared data between each species pair; arthropod core ortholog matrix (matrix 5, Table 2). Degree of positive data overlap indicated by a color-coded heatmap (yellow = low, red = high); species order from right to left in the same order as listed from top to bottom on right side of figure. Lack of phylogenetic clustering indicates bias from shared data does not explain relationships seen in phylogenomic analyses.

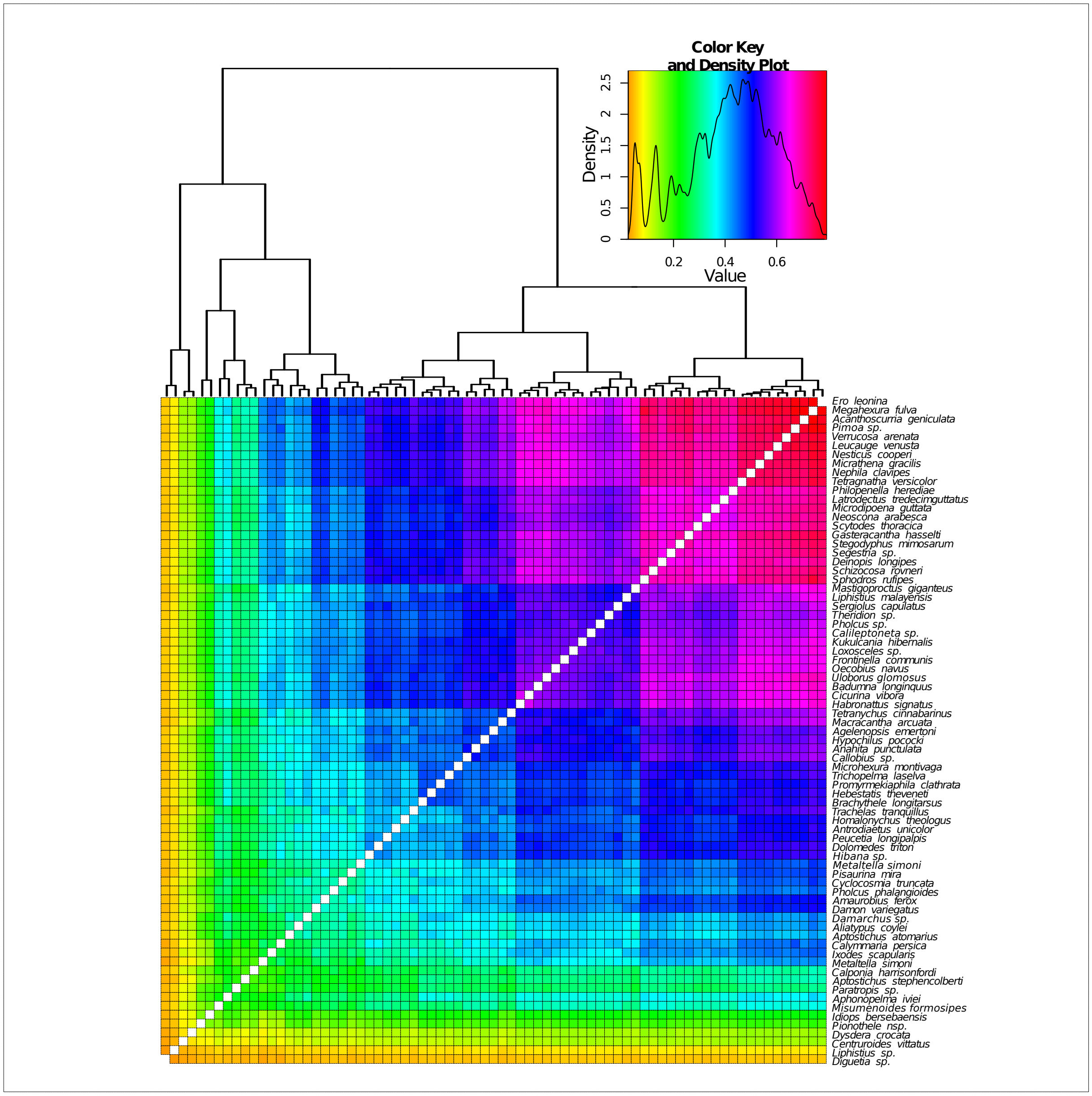

Figure 5. Clustering/heatmap analysis depicting degree of shared data between each species pair; full spider ortholog matrix (matrix 1, Table 2). Degree of positive data overlap indicated by a color-coded heatmap (yellow = low, red = high); species order from right to left in the same order as listed from top to bottom on right side of figure. Lack of phylogenetic clustering indicates bias from shared data does not explain relationships seen in phylogenomic analyses.

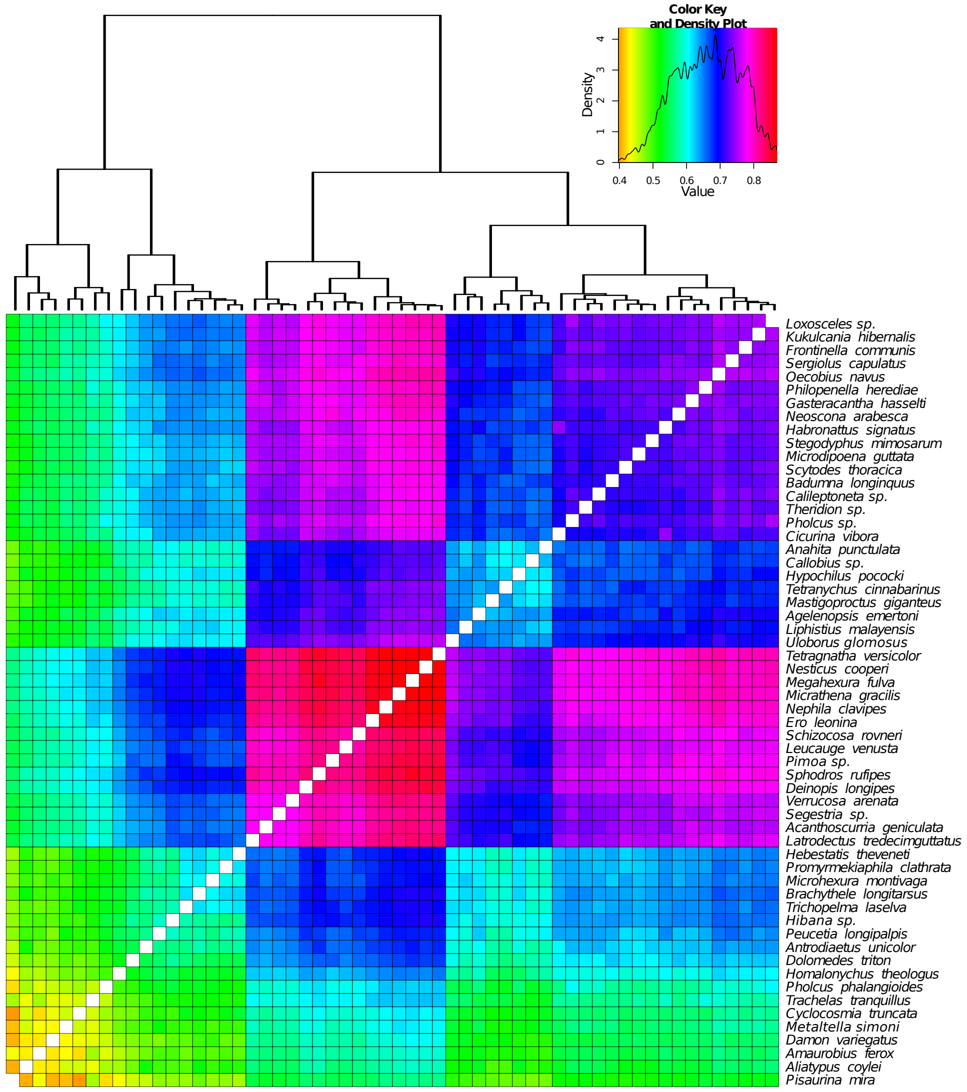

Figure 6. Clustering/heatmap analysis depicting degree of shared data between each species pair; MARE matrix (matrix 7, Table 2). Degree of positive data overlap indicated by a color-coded heatmap (yellow = low, red = high); species order from right to left in the same order as listed from top to bottom on right side of figure. Lack of phylogenetic clustering indicates bias from shared data does not explain relationships seen in phylogenomic analyses.

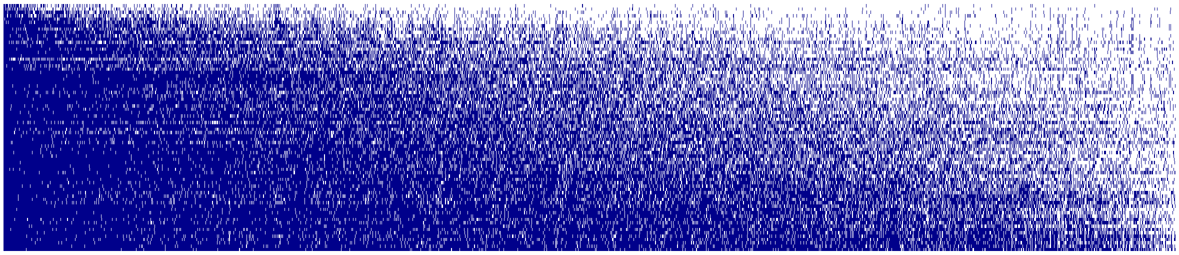

Figure 7. Gene occupancy of matrix 1 (see Table 2). Colored squares represent partitions present in matrix for each OTU (x-axis, in descending order of OTU representation from left to right) and each partition or gene (y-axis, in ascending order of partition representation).

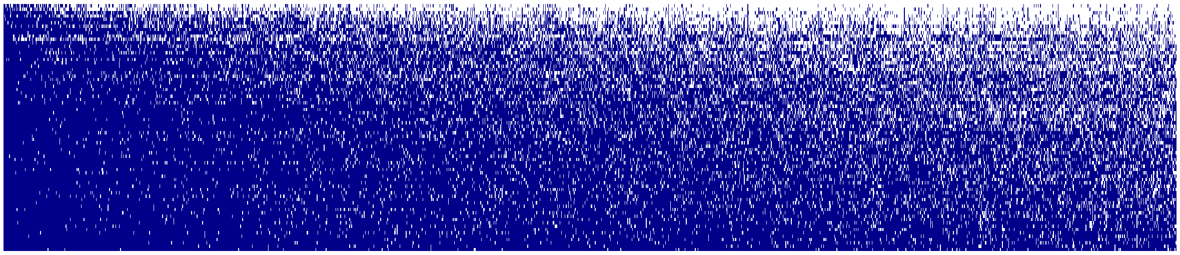

Figure 8. Gene occupancy of matrix 2 (see Table 2). Colored squares represent partitions present in matrix for each OTU (x-axis, in descending order of OTU representation from left to right) and each partition or gene (y-axis, in ascending order of partition representation).

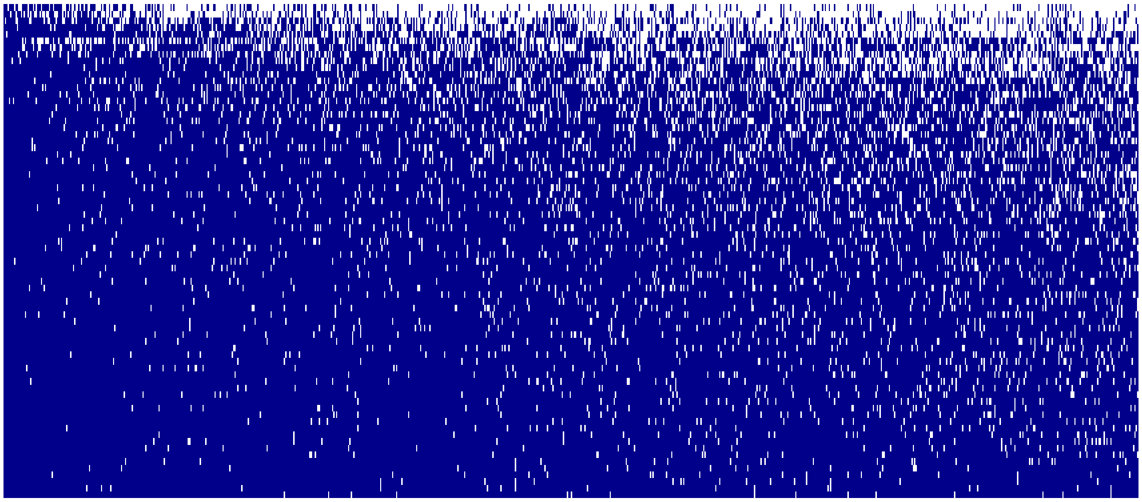

Figure 9. Gene occupancy of matrix 3 (see Table 2). Colored squares represent partitions present in matrix for each OTU (y-axis, in descending order of OTU representation from bottom to top) and each partition or gene (x-axis, in descending order of partition representation from left to right).

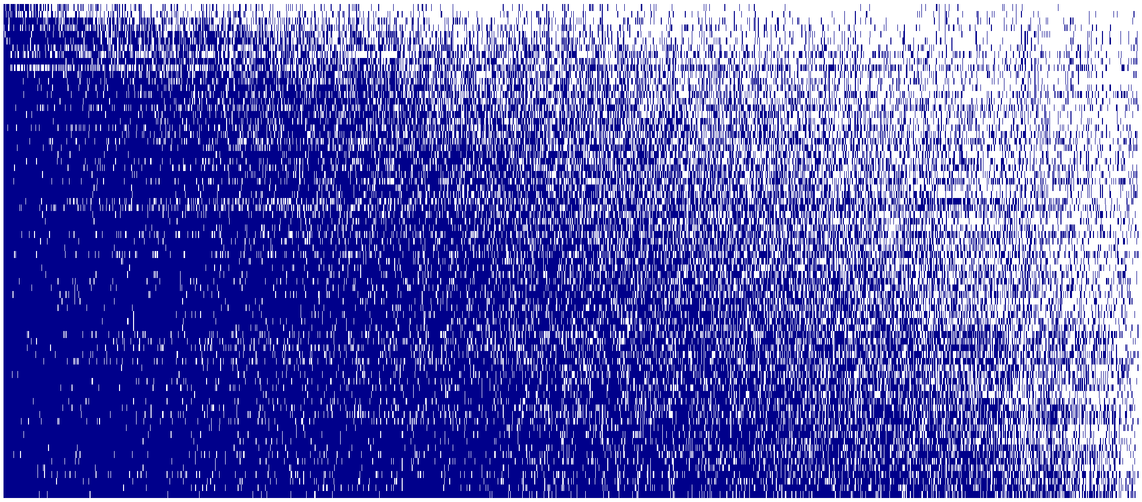

Figure 10. Gene occupancy of matrix 4 (see Table 2). Colored squares represent partitions present in matrix for each OTU (y-axis, in descending order of OTU representation from bottom to top) and each partition or gene (x-axis, in descending order of partition representation from left to right).

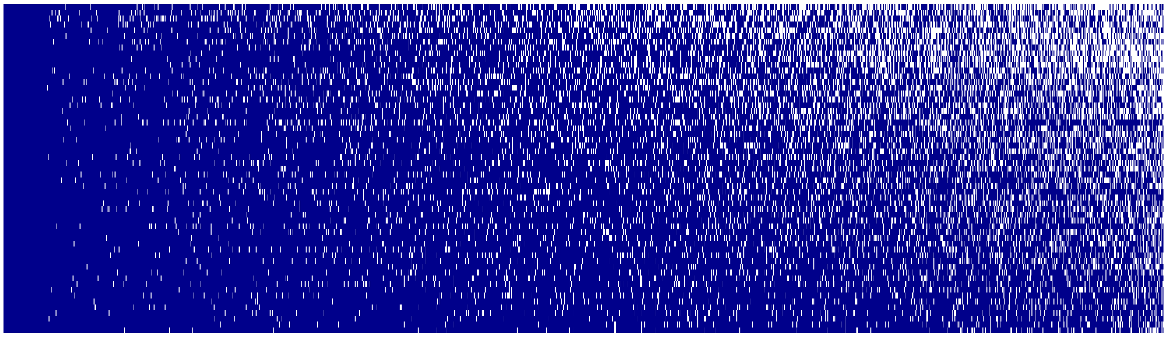

Figure 11. Gene occupancy of matrix 7 (see Table 2). Colored squares represent partitions present in matrix for each OTU (y-axis, in descending order of OTU representation from bottom to top) and each partition or gene (x-axis, in descending order of partition representation from left to right).

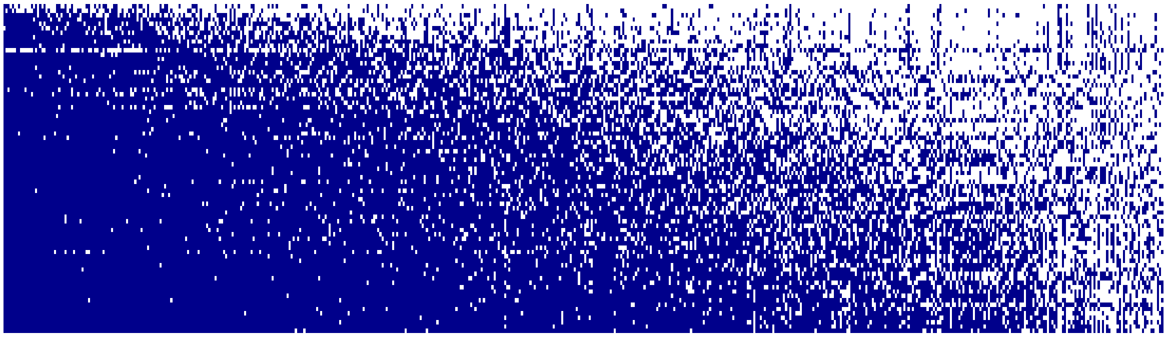

Figure 12. Gene occupancy of matrix 5 (see Table 2). Colored squares represent partitions present in matrix for each OTU (y-axis, in descending order of OTU representation from bottom to top) and each partition or gene (x-axis, in descending order of partition representation from left to right).

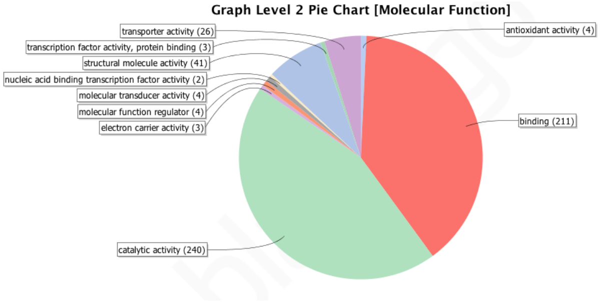

Figure 13. Gene Ontology molecular functions, levels 2 for OGs shared by Arthropod and Spider Core sets. Figures generated by Blast2GO analysis.

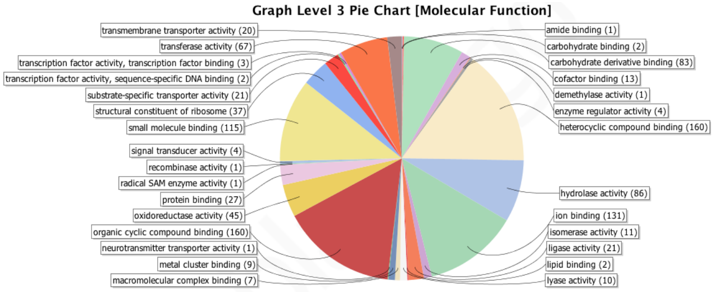

Figure 14. Gene Ontology molecular functions, level 3 for OGs shared by Arthropod and Spider Core sets. Figures generated by Blast2GO analysis.
