# Supplementary material for: Spider phylogenomics: untangling the Spider Tree of Life
Source: PeerJ. 2016 Feb 23;4:e1719. doi: 10.7717/peerj.1719 (PMC4768681; doi:10.7717/peerj.1719)
Supplement: Supplemental Information 5 [file peerj-04-1719-s019.pdf]

Table 3. Spider Core Ortholog Annotations. BLASTP (top hit, evalue 1E-10) of Spider Core OGs found in reference taxon (Acanthoscurria geniculata) against either 1) custom BLAST database of Stegodyphus mimosarum proteins downloaded from GenBank (BioProject Accession PRJNA222714; 27,135 protein sequences) or 2) nr, the non-redundant protein database maintained by NCBI. Arthropod Core OG matches to Spider Core OG listed in first column.

Arthropod Core ID	Spider Core ID	Putative Ortholog Description	Target Database
NA	356	Protein spinster partial	custom
NA	358	MAP/microtubule affinity-regulating kinase 3 partial	custom
NA	359	Metastasis-associated protein MTA1 partial	custom
NA	360	Oxysterol-binding protein-related protein 9 partial	custom
NA	363	NAD-dependent deacetylase sirtuin-1 partial	custom
NA	365	putative fatty acyl-CoA reductase partial	custom
90433	369	Superkiller viralicidic activity 2-like 2 partial	custom
NA	370	hypothetical protein X975 26308 partial	custom
NA	371	5'-3' exoribonuclease 2 partial	custom
90338	372	Calcium homeostasis endoplasmic reticulum protein partial	custom
NA	375	Lysine-specific demethylase 5A partial	custom
NA	382	MAGUK p55 subfamily member 5 partial	custom
NA	383	Catenin alpha partial	custom
NA	388	Protein alan shepard partial	custom
NA	389	Caskin-1 partial	custom
NA	392	Glycerophosphocholine phosphodiesterase GPCPD1 partial	custom
NA	393	Ataxin-2 partial	custom

Supplemental Table 3 – continued from previous page

Arthropod Core ID	Spider Core ID	Putative Ortholog Description	Target Database
NA	395	Transcription elongation factor SPT6 partial	custom
NA	399	E3 ubiquitin-protein ligase UBR2 partial	custom
NA	410	Phosphatidylinositol-binding clathrin assembly protein LAP partial	custom
NA	411	Sushi von Willebrand factor type A EGF and pentraxin domain-containing protein 1 partial	custom
NA	413	Cubilin partial	custom
NA	415	Ras GTPase-activating protein 1 partial	custom
NA	416	Cytoplasmic dynein 1 intermediate chain 2 partial	custom
89661	418	Bifunctional 3'-phosphoadenosine 5'-phosphosulfate synthase partial	custom
NA	419	Glycogen synthase kinase-3 beta partial	custom
NA	422	Irregular chiasm C-roughest protein partial	custom
89807	423	Actin-interacting protein 1 partial	custom
NA	425	hypothetical protein X975 25889 partial	custom
NA	426	Transforming growth factor-beta-induced protein ig-h3 partial	custom
NA	427	Coatomer subunit alpha partial	custom
NA	429	putative E3 ubiquitin-protein ligase MYCBP2 partial	custom
NA	430	Macoilin-1 partial	custom
90507	431	Stress-activated protein kinase JNK partial	custom
NA	432	5'-3' exoribonuclease 1 partial	custom

Supplemental Table 3 – continued from previous page

Arthropod Core ID	Spider Core ID	Putative Ortholog Description	Target Database
NA	437	U5 small nuclear ribonucleoprotein helicase partial	custom
90171	438	U5 small nuclear ribonucleoprotein component partial	custom
90478	439	Nucleobindin-1 partial	custom
NA	440	Protein transport protein Sec24A partial	custom
NA	441	hypothetical protein X975 00738 partial	custom
NA	443	Receptor-type tyrosine-protein phosphatase mu partial	custom
90468	444	Beta-galactosidase partial	custom
NA	446	hypothetical protein X975 09242 partial	custom
NA	449	Tyrosine-protein kinase PR2 partial	custom
NA	454	Muscle M-line assembly protein unc-89 partial	custom
90448	455	Endoplasmin partial	custom
89844	456	Ubiquitin-like modifier-activating enzyme 1 partial	custom
90063	457	hypothetical protein X975 10054 partial	custom
90057	460	Glycogen debranching enzyme partial	custom
NA	462	Protein LSM14-like protein partial	custom
89946	464	Integrin alpha-9 partial	custom
NA	465	Lysine-specific demethylase 2B partial	custom
90251	466	Ubiquitin-like modifier-activating enzyme ATG7 partial	custom
NA	470	Glycerol-3-phosphate acyltransferase 1 mitochondrial partial	custom

Supplemental Table 3 – continued from previous page

Arthropod Core ID	Spider Core ID	Putative Ortholog Description	Target Database
NA	473	Serine/threonine-protein phosphatase 6 regulatory subunit 3 partial	custom
NA	474	KAT8 regulatory NSL complex subunit 1 partial	custom
89714	475	UDP-glucose:glycoprotein glucosyltransferase 1 partial	custom
90034	476	Sterol regulatory element-binding protein 1 partial	custom
NA	477	Glypican-6 partial	custom
90536	478	Pyruvate kinase muscle isozyme partial	custom
NA	480	Influenza virus NS1A-binding protein-like protein partial	custom
90317	481	Armadillo segment polarity protein partial	custom
NA	482	ATP-dependent RNA helicase DDX1 partial	custom
90596	484	Leucine–tRNA ligase cytoplasmic partial	custom
89750	485	Eukaryotic peptide chain release factor GTP-binding subunit ERF3A partial	custom
90499	486	Serrate RNA effector molecule-like protein partial	custom
89876	491	Exportin-1 partial	custom
NA	504	Oxysterol-binding protein 2 partial	custom
NA	505	Serine/threonine-protein kinase tousled-like 1 partial	custom
89918	509	Splicing factor 3A subunit 1 partial	custom
NA	510	Band 4.1-like protein 4A partial	custom
90393	512	FACT complex subunit spt16 partial	custom

Supplemental Table 3 – continued from previous page

Arthropod Core ID	Spider Core ID	Putative Ortholog Description	Target Database
NA	513	ATP-citrate synthase partial	custom
NA	514	Choline/ethanolaminephosphotransferase 1 partial	custom
89829	518	Ubiquitin carboxyl-terminal hydrolase 7 partial	custom
NA	519	Carnitine O-palmitoyltransferase 1 liver isoform partial	custom
90354	520	Pre-mRNA-processing factor 40-like protein partial	custom
NA	522	CAP-Gly domain-containing linker protein 1 partial	custom
NA	524	Carboxypeptidase D partial	custom
NA	525	hypothetical protein X975 17021 partial	custom
NA	529	Eukaryotic initiation factor 4A-III partial	custom
89912	534	AP-2 complex subunit alpha-2 partial	custom
NA	535	hypothetical protein X975 00501 partial	custom
NA	537	hypothetical protein X975 01393 partial	custom
NA	539	Phytanoyl-CoA dioxygenase peroxisomal partial	custom
NA	540	Cullin-1 partial	custom
NA	541	Lethal(3)malignant brain tumor-like protein 3 partial	custom
89995	542	putative ATP-dependent RNA helicase DDX23 partial	custom
NA	544	Ataxin-2 partial	custom
NA	545	hypothetical protein X975 18913 partial	custom
NA	549	Kynurenine–oxoglutarate transaminase 3 partial	custom
NA	550	Poly(U)-binding-splicing factor half pint partial	custom

Supplemental Table 3 – continued from previous page

Arthropod Core ID	Spider Core ID	Putative Ortholog Description	Target Database
89734	551	putative phospholipid-transporting ATPase IIB partial	custom
NA	553	hypothetical protein X975 21588 partial	custom
NA	554	Zinc finger X-linked protein ZXDB partial	custom
NA	560	Bone morphogenetic protein receptor type-2 partial	custom
90335	561	Threonine–tRNA ligase cytoplasmic partial	custom
90055	562	Valine–tRNA ligase partial	custom
NA	563	AP-3 complex subunit delta-1 partial	custom
90418	565	cAMP-dependent protein kinase regulatory subunit partial	custom
NA	567	Inactive dipeptidyl peptidase 10 partial	custom
NA	568	Uridine-cytidine kinase-like 1 partial	custom
NA	569	hypothetical protein X975 14140 partial	custom
NA	570	Protein transport protein Sec23A partial	custom
NA	572	Integrator complex subunit 4 partial	custom
NA	574	putative ATP-dependent RNA helicase TDRD9 partial	custom
NA	576	Ubiquitin carboxyl-terminal hydrolase 47 partial	custom
NA	577	hypothetical protein X975 19997 partial	custom
NA	582	Vitellogenin-6 partial	custom
NA	584	Protein phosphatase 1B partial	custom
NA	585	Protein FAM134C partial	custom
NA	586	YTH domain family protein 1 partial	custom

Supplemental Table 3 – continued from previous page

Arthropod Core ID	Spider Core ID	Putative Ortholog Description	Target Database
NA	587	Ral GTPase-activating protein subunit beta partial	custom
NA	588	Vacuolar protein sorting-associated protein 35 partial	custom
NA	590	Ubiquitin thioesterase Zranb1 partial	custom
NA	592	hypothetical protein X975 10048 partial	custom
NA	595	Angiotensin-converting enzyme partial	custom
89663	597	Procollagen-lysine2-oxoglutarate 5-dioxygenase 3 partial	custom
NA	598	Tropomodulin partial	custom
NA	600	Ectonucleoside triphosphate diphosphohydrolase 1 partial	custom
89883	601	Monocarboxylate transporter 14 partial	custom
90447	602	Aconitate hydratase mitochondrial partial	custom
NA	603	hypothetical protein X975 15148 partial	custom
NA	604	Acetyl-coenzyme A synthetase cytoplasmic partial	custom
NA	605	ATP-sensitive inward rectifier potassium channel 12 partial	custom
NA	609	Homeobox protein cut partial	custom
NA	610	Pumilio-like protein partial	custom
NA	613	Protein Hook-like protein partial	custom
NA	615	Trithorax group protein osa partial	custom
90516	619	Glutamine synthetase 2 cytoplasmic partial	custom
90333	620	Non-specific lipid-transfer protein partial	custom

Supplemental Table 3 – continued from previous page

Arthropod Core ID	Spider Core ID	Putative Ortholog Description	Target Database
NA	621	E3 ubiquitin-protein ligase HUWE1 partial	custom
NA	622	Stathmin-1-A partial	custom
NA	623	Neuropathy target esterase partial	custom
90470	624	Serine/threonine-protein kinase TAO1 partial	custom
89658	625	Nuclease domain-containing protein 1 partial	custom
NA	627	Cytoplasmic polyadenylation element-binding protein 1-B partial	custom
NA	631	Vacuolar protein sorting-associated protein 41-like protein partial	custom
NA	632	E3 ubiquitin-protein ligase RNF123 partial	custom
NA	633	Pyruvate dehydrogenase phosphatase regulatory subunit mitochondrial partial	custom
NA	635	hypothetical protein X975 12682 partial	custom
NA	636	Ring canal kelch-like protein partial	custom
NA	641	hypothetical protein X975 17022 partial	custom
90318	644	Chloride channel protein 2 partial	custom
NA	650	Homogentisate 12-dioxygenase partial	custom
90595	652	hypothetical protein X975 02019 partial	custom
NA	653	Proteasome activator complex subunit 4 partial	custom
NA	655	Secretory carrier-associated membrane protein 1 partial	custom

Supplemental Table 3 – continued from previous page

Arthropod Core ID	Spider Core ID	Putative Ortholog Description	Target Database
90300	656	Nuclear export mediator factor Nemf partial	custom
89830	660	Carboxypeptidase M partial	custom
NA	661	Cytosolic purine 5'-nucleotidase partial	custom
NA	664	GMP synthase [glutamine-hydrolyzing] partial	custom
NA	665	Neuropilin and tolloid-like protein 2 partial	custom
90247	666	Myosin heavy chain 95F partial	custom
NA	668	Upstream stimulatory factor 2 partial	custom
NA	669	TBC1 domain family member 9 partial	custom
NA	677	PHD and RING finger domain-containing protein 1 partial	custom
NA	680	Apoptosis-stimulating of p53 protein 1 partial	custom
NA	681	putative hexokinase HKDC1 partial	custom
NA	684	Ribosome-binding protein 1 partial	custom
90510	686	Arginine kinase partial	custom
NA	688	Hepatocyte growth factor-regulated tyrosine kinase substrate partial	custom
NA	689	Striatin-3 partial	custom
NA	691	Cullin-3-A partial	custom
NA	692	Laminin subunit alpha-2 partial	custom
NA	693	hypothetical protein X975 05141 partial	custom
90142	697	Protein unc-45-like protein partial	custom
90353	699	Protein henna partial	custom
NA	700	hypothetical protein X975 22176 partial	custom

Supplemental Table 3 – continued from previous page

Arthropod Core ID	Spider Core ID	Putative Ortholog Description	Target Database
NA	703	Alpha-taxilin partial	custom
NA	705	Phosphatidylinositol 4-kinase beta partial	custom
NA	707	Zinc finger FYVE domain-containing protein 1 partial	custom
NA	708	Cyclic AMP-responsive element-binding protein 3-like protein 3 partial	custom
NA	711	E3 ubiquitin-protein ligase LRSAM1 partial	custom
89656	717	Nipped-B-like protein partial	custom
NA	719	Protein flightless-1 partial	custom
NA	724	hypothetical protein X975 08766 partial	custom
NA	725	Choline-phosphate cytidylyltransferase B partial	custom
89902	726	putative ATP-dependent RNA helicase DDX4 partial	custom
NA	727	Isocitrate dehydrogenase [NADP] mitochondrial partial	custom
90358	728	Cyclin-dependent kinase 2 partial	custom
NA	729	C-1-tetrahydrofolate synthase cytoplasmic partial	custom
NA	730	PDZ and LIM domain protein Zasp partial	custom
NA	731	cAMP-specific 3'5'-cyclic phosphodiesterase partial	custom
NA	732	Syntaxin-binding protein 5 partial	custom
NA	733	Histone-lysine N-methyltransferase EZH2 partial	custom
NA	734	26S proteasome non-ATPase regulatory subunit 1 partial	custom

Supplemental Table 3 – continued from previous page

Arthropod Core ID	Spider Core ID	Putative Ortholog Description	Target Database
NA	735	Importin-9 partial	custom
NA	737	hypothetical protein X975 03177 partial	custom
NA	738	Regulator of chromosome condensation partial	custom
NA	739	putative palmitoyl-transferase ZDHHC8 partial	custom
NA	740	Unconventional myosin-XV partial	custom
NA	746	Mannosyl-oligosaccharide alpha-12-mannosidase isoform A partial	custom
89937	748	[Pyruvate dehydrogenase [lipoamide]] kinase mitochondrial partial	custom
NA	750	Exocyst complex component 6B partial	custom
NA	751	Katanin p80 WD40-containing subunit B1 partial	custom
NA	752	Transmembrane and coiled-coil domain-containing protein 7 partial	custom
NA	757	Protein MON2-like protein partial	custom
NA	758	Zinc finger FYVE domain-containing protein 9 partial	custom
NA	761	hypothetical protein X975 06477 partial	custom
NA	763	Protein diaphanous-like protein partial	custom
NA	767	Calcium uptake protein 1-like protein mitochondrial partial	custom
NA	770	Solute carrier organic anion transporter family member 4A1 partial	custom
NA	771	Restin-like protein partial	custom

Supplemental Table 3 – continued from previous page

Arthropod Core ID	Spider Core ID	Putative Ortholog Description	Target Database
NA	772	PDZ and LIM domain protein Zasp partial	custom
NA	773	Homer protein-like protein partial	custom
NA	775	Allene oxide synthase-lipoxygenase protein partial	custom
NA	777	Tumor susceptibility 101 protein partial	custom
NA	779	Solute carrier family 12 member 4 partial	custom
NA	782	putative gamma-glutamyltransferase ywrD partial	custom
NA	783	Heat shock protein 12A partial	custom
NA	784	Coagulation factor X partial	custom
NA	785	Endothelin-converting enzyme 2 partial	custom
NA	788	Lysyl oxidase-like protein partial	custom
NA	789	Cysteine and glycine-rich protein 2 partial	custom
NA	792	Chondroitin sulfate N-acetylgalactosaminyltransferase 1 partial	custom
NA	793	Stearoyl-CoA desaturase 5 partial	custom
89752	794	hypothetical protein X975 01626 partial	custom
NA	797	StAR-related lipid transfer protein 13 partial	custom
NA	800	Collagen type IV alpha-3-binding protein partial	custom
NA	801	Nuclear receptor-binding protein-like protein partial	custom
90155	803	Cullin-4B partial	custom
NA	804	Interference hedgehog partial	custom

Supplemental Table 3 – continued from previous page

Arthropod Core ID	Spider Core ID	Putative Ortholog Description	Target Database
NA	805	hypothetical protein X975 01883 partial	custom
NA	808	Nostrin partial	custom
NA	809	Ubiquitin carboxyl-terminal hydrolase 32 partial	custom
NA	812	F-box-like/WD repeat-containing protein TBL1XR1 partial	custom
NA	814	Golgi reassembly-stacking protein 2 partial	custom
NA	815	SEC23-interacting protein partial	custom
89733	817	Coatomer subunit beta partial	custom
NA	818	Peroxisomal targeting signal 1 receptor partial	custom
NA	819	Syntaxin-7 partial	custom
NA	821	Clathrin interactor 1 partial	custom
89869	823	WASH complex subunit strumpellin partial	custom
NA	824	Protein cereblon partial	custom
NA	825	hypothetical protein X975 06244 partial	custom
NA	827	Ribosome biogenesis protein BMS1-like protein partial	custom
NA	829	Aldehyde dehydrogenase family 3 member B1 partial	custom
NA	830	Lipase maturation factor 2 partial	custom
NA	832	Neural cell adhesion molecule 1-A partial	custom
NA	833	hypothetical protein X975 18325 partial	custom
NA	835	hypothetical protein X975 17516 partial	custom
90215	840	DNA damage-binding protein 1 partial	custom

Supplemental Table 3 – continued from previous page

Arthropod Core ID	Spider Core ID	Putative Ortholog Description	Target Database
NA	841	BAI1-associated protein 3 partial	custom
90126	842	ATP-dependent RNA helicase abstrakt partial	custom
NA	845	putative adenosylhomocysteinase 3 partial	custom
NA	847	Transcription factor CP2 partial	custom
NA	851	Heterogeneous nuclear ribonucleoprotein K partial	custom
NA	853	U5 small nuclear ribonucleoprotein helicase partial	custom
NA	856	Dipeptidyl peptidase 1 partial	custom
89646	857	UTP–glucose-1-phosphate uridylyltransferase partial	custom
NA	859	U4/U6 small nuclear ribonucleoprotein Prp3 partial	custom
89888	861	Protein arginine N-methyltransferase 1 partial	custom
NA	862	Myotubularin-related protein 3 partial	custom
90431	863	Poly(A) polymerase gamma partial	custom
NA	864	Programmed cell death 6-interacting protein partial	custom
NA	868	F-box/WD repeat-containing protein 7 partial	custom
NA	874	Short spindle protein 4 partial	custom
NA	875	Alkaline phosphatase tissue-nonspecific isozyme partial	custom
NA	876	Extracellular domains-containing protein partial	custom
NA	877	Crooked neck-like protein 1 partial	custom

Supplemental Table 3 – continued from previous page

Arthropod Core ID	Spider Core ID	Putative Ortholog Description	Target Database
90122	879	putative glycogen [starch] synthase partial	custom
NA	880	WD repeat and FYVE domain-containing protein 2 partial	custom
NA	881	Nuclear hormone receptor E75 partial	custom
NA	884	Eukaryotic translation initiation factor 2A partial	custom
NA	886	AT-rich interactive domain-containing protein 5B partial	custom
NA	887	DNA polymerase sigma partial	custom
NA	888	FGGY carbohydrate kinase domain-containing protein partial	custom
NA	890	Trafficking protein particle complex subunit 12 partial	custom
NA	892	hypothetical protein X975 17435 partial	custom
NA	895	PREDICTED:transmembrane protein 131-like	nr
NA	896	putative oxidoreductase GLYR1-like protein partial	custom
NA	897	Protein mothers against dpp partial	custom
NA	903	hypothetical protein X975 20223 partial	custom
NA	909	RRP12-like protein partial	custom
89759	910	Sodium- and chloride-dependent GABA transporter 1 partial	custom
NA	911	Differentially expressed in FDCP 8-like protein partial	custom
89701	912	Zinc finger RNA-binding protein partial	custom

Supplemental Table 3 – continued from previous page

Arthropod Core ID	Spider Core ID	Putative Ortholog Description	Target Database
NA	914	Serine/threonine-protein kinase minibrain partial	custom
NA	915	MOG interacting and ectopic P-granules protein 1 partial	custom
NA	916	Splicing factor proline- and glutamine-rich partial	custom
90537	917	Eukaryotic translation initiation factor 3 subunit A partial	custom
NA	918	putative phospholipase B-like 2 partial	custom
NA	921	28S ribosomal protein S23 mitochondrial partial	custom
NA	922	Alpha-L-fucosidase partial	custom
NA	924	Phosphoinositide 3-kinase regulatory subunit 4 partial	custom
NA	925	Cellular tumor antigen p53 partial	custom
NA	927	3-hydroxy-3-methylglutaryl-coenzyme A reductase partial	custom
90382	929	Paraplegin partial	custom
NA	931	Protein phosphatase 1 regulatory subunit 16A partial	custom
NA	932	Choline kinase alpha partial	custom
NA	934	NA	nr
NA	935	GTP-binding protein 1 partial	custom
NA	936	Excitatory amino acid transporter 2 partial	custom
NA	937	hypothetical protein X975 06292 partial	custom
NA	939	Serine proteinase stubble partial	custom

Supplemental Table 3 – continued from previous page

Arthropod Core ID	Spider Core ID	Putative Ortholog Description	Target Database
NA	940	Macrophage mannose receptor 1 partial	custom
NA	942	hypothetical protein X975 27120 partial	custom
NA	944	hypothetical protein X975 12055 partial	custom
NA	945	hypothetical protein X975 24338 partial	custom
NA	947	Insulin receptor substrate 2-B partial	custom
90168	948	Glycerol-3-phosphate dehydrogenase mitochondrial partial	custom
89758	950	Alanine–glyoxylate aminotransferase 2 mitochondrial partial	custom
NA	952	Cystathionine beta-synthase partial	custom
90181	953	Transmembrane GTPase Marf partial	custom
89904	954	Cell division cycle protein 23-like protein partial	custom
NA	961	hypothetical protein X975 05470 partial	custom
NA	969	Elongation factor 1-alpha 1 partial	custom
NA	970	Armadillo repeat-containing protein 8 partial	custom
NA	973	Titin partial	custom
NA	975	Tetratricopeptide repeat protein 38 partial	custom
NA	976	Sodium/potassium/calcium exchanger 6 partial	custom
NA	978	Alkyldihydroxyacetonephosphate synthase peroxisomal partial	custom
NA	979	putative cation-transporting ATPase 13A3 partial	custom
89884	980	Exportin-2 partial	custom
NA	981	hypothetical protein X975 08408 partial	custom
NA	982	Vinculin partial	custom

Supplemental Table 3 – continued from previous page

Arthropod Core ID	Spider Core ID	Putative Ortholog Description	Target Database
NA	984	Plastin-2 partial	custom
NA	985	OTU domain-containing protein 5 partial	custom
NA	987	Ran-binding protein 9 partial	custom
NA	988	Splicing factor 3B subunit 3 partial	custom
90165	992	Coatomer subunit gamma-2 partial	custom
NA	993	Next to BRCA1 1 protein partial	custom
NA	994	Transforming protein p54/c-ets-1 partial	custom
NA	996	hypothetical protein X975 15012 partial	custom
NA	997	Vigilin partial	custom
NA	998	CUB and sushi domain-containing protein 3 partial	custom
89857	1000	Peroxisomal multi-functional enzyme type 2 partial	custom
90292	1001	Nck-associated protein 1 partial	custom
NA	1003	Segmentation protein cap'n'collar partial	custom
NA	1004	Sodium/hydrogen exchanger 7 partial	custom
NA	1007	cAMP-specific 3'5'-cyclic phosphodiesterase 7B partial	custom
NA	1008	RNA-binding protein 28 partial	custom
NA	1010	Acetolactate synthase-like protein partial	custom
NA	1011	Cytosolic 10-formyltetrahydrofolate dehydrogenase partial	custom
NA	1012	Polynucleotide 5'-hydroxyl-kinase NOL9 partial	custom
NA	1015	Zinc transporter ZIP9-A partial	custom

Supplemental Table 3 – continued from previous page

Arthropod Core ID	Spider Core ID	Putative Ortholog Description	Target Database
NA	1016	Calcium/calmodulin-dependent protein kinase kinase 2 partial	custom
NA	1017	Phosphatidylinositide phosphatase SAC1 partial	custom
NA	1019	hypothetical protein X975 10524 partial	custom
90082	1020	Calcium/calmodulin-dependent protein kinase type 1 partial	custom
NA	1023	hypothetical protein X975 22675 partial	custom
NA	1024	Elongator complex protein 1 partial	custom
NA	1030	Exocyst complex component 5 partial	custom
NA	1034	Monocarboxylate transporter 9 partial	custom
NA	1035	Double-stranded RNA-binding protein Staufen-like protein partial	custom
NA	1036	FK506-binding protein 15 partial	custom
NA	1037	Vam6/Vps39-like protein partial	custom
NA	1040	Transmembrane and coiled-coil domains protein 2 partial	custom
NA	1041	LIM and SH3 domain protein Lasp partial	custom
NA	1042	Metastasis-associated protein MTA1 partial	custom
NA	1043	Sodium-independent sulfate anion transporter partial	custom
NA	1044	Oxidation resistance protein 1 partial	custom
NA	1045	Lysosomal protective protein partial	custom
90377	1046	Dynamin-like protein mitochondrial partial	custom
90302	1047	U1 small nuclear ribonucleoprotein partial	custom

Supplemental Table 3 – continued from previous page

Arthropod Core ID	Spider Core ID	Putative Ortholog Description	Target Database
NA	1048	ADP-ribosylation factor GTPase-activating protein 2 partial	custom
90363	1049	Phosphoenolpyruvate carboxykinase [GTP] mitochondrial partial	custom
90616	1050	Phosphatidylinositol 3-kinase catalytic subunit type 3 partial	custom
NA	1051	YTH domain-containing protein 1 partial	custom
90489	1053	Dual oxidase 2 partial	custom
89815	1054	Alpha-2-macroglobulin receptor-associated protein partial	custom
NA	1055	DnaJ-like protein subfamily B member 2 partial	custom
NA	1056	Chromobox protein-like protein partial	custom
NA	1058	RNA-binding protein MEX3B partial	custom
NA	1059	DnaJ-like protein subfamily B member 4 partial	custom
NA	1060	Golgin subfamily A member 2 partial	custom
90096	1063	Calcium-binding mitochondrial carrier protein Aralar1 partial	custom
NA	1064	hypothetical protein X975 02870 partial	custom
NA	1065	Protein GDAP2-like protein partial	custom
NA	1068	Rap guanine nucleotide exchange factor 4 partial	custom
NA	1069	Cip1-interacting zinc finger protein partial	custom
90585	1070	Methylcrotonoyl-CoA carboxylase beta chain mitochondrial partial	custom
NA	1071	Lysyl oxidase-like protein partial	custom

Supplemental Table 3 – continued from previous page

Arthropod Core ID	Spider Core ID	Putative Ortholog Description	Target Database
NA	1073	Multiple inositol polyphosphate phosphatase 1 partial	custom
NA	1074	Transforming growth factor-beta-induced protein ig-h3 partial	custom
89898	1075	Succinate dehydrogenase [ubiquinone] flavoprotein subunit mitochondrial partial	custom
90396	1076	Transitional endoplasmic reticulum ATPase TER94 partial	custom
NA	1077	hypothetical protein X975 21094 partial	custom
89711	1079	Eukaryotic translation initiation factor 3 subunit B partial	custom
90397	1081	Coronin-7 partial	custom
89991	1082	Calsyntenin-1 partial	custom
NA	1083	hypothetical protein X975 02271 partial	custom
89775	1086	putative RNA-binding protein Luc7-like 2 partial	custom
NA	1088	WD repeat-containing protein mio partial	custom
NA	1090	X-ray repair cross-complementing protein 5 partial	custom
NA	1091	Protein PAT1-like protein partial	custom
89672	1092	Signal transducer and activator of transcription 5A partial	custom
NA	1096	Luc7-like protein 3 partial	custom
NA	1097	Lymphoid-restricted membrane protein partial	custom
NA	1098	F-box only protein 18 partial	custom
NA	1100	CREB-binding protein partial	custom
NA	1101	Hephaestin-like protein 1 partial	custom

Supplemental Table 3 – continued from previous page

Arthropod Core ID	Spider Core ID	Putative Ortholog Description	Target Database
NA	1108	Cysteine protease ATG4B partial	custom
NA	1109	Serine/threonine-protein kinase LATS1 partial	custom
NA	1110	Rhophilin-2 partial	custom
NA	1111	G protein-coupled receptor kinase 1 partial	custom
NA	1118	Protein FAM172A partial	custom
NA	1119	ATP-dependent RNA helicase A partial	custom
90362	1122	Histone H3.3 partial	custom
NA	1123	23-bisphosphoglycerate-independent phosphoglycerate mutase partial	custom
NA	1124	Histone-arginine methyltransferase CARMER partial	custom
NA	1125	hypothetical protein X975 00161 partial	custom
NA	1126	Scaffold attachment factor B2 partial	custom
NA	1127	Protein tweety partial	custom
NA	1129	Sodium/calcium exchanger 3 partial	custom
89834	1130	DNA topoisomerase 1 partial	custom
NA	1131	Neurocalcin-like protein partial	custom
90042	1132	Serine/threonine-protein kinase VRK1 partial	custom
NA	1133	Transcriptional repressor protein YY1 partial	custom
NA	1135	Glutaredoxin domain-containing cysteine-rich protein partial	custom
NA	1136	Cytoplasmic dynein 1 light intermediate chain 2 partial	custom
NA	1137	Lon protease-like protein peroxisomal partial	custom
NA	1139	Metaxin-2 partial	custom

Supplemental Table 3 – continued from previous page

Arthropod Core ID	Spider Core ID	Putative Ortholog Description	Target Database
NA	1140	Programmed cell death protein 4 partial	custom
NA	1141	5-aminolevulinate synthase nonspecific mitochondrial partial	custom
NA	1142	NA	nr
89885	1143	putative ATP-dependent RNA helicase me31b partial	custom
90500	1144	Nucleoside diphosphate-linked moiety X motif 19 mitochondrial partial	custom
90170	1146	Thrombospondin-4 partial	custom
NA	1147	hypothetical protein X975 14086 partial	custom
NA	1148	Protein FAM46C partial	custom
NA	1149	hypothetical protein X975 05113 partial	custom
NA	1150	Thimet oligopeptidase partial	custom
NA	1152	Cysteine sulfinic acid decarboxylase partial	custom
NA	1153	putative helicase with zinc finger partial	custom
NA	1154	Very long-chain specific acyl-CoA dehydrogenase mitochondrial partial	custom
NA	1155	Monocarboxylate transporter 12 partial	custom
NA	1157	Steryl-sulfatase partial	custom
90559	1158	Pre-mRNA-splicing factor RBM22 partial	custom
NA	1159	Protein KRI1-like protein partial	custom
NA	1160	putative ATP-dependent RNA helicase DDX46 partial	custom
NA	1163	hypothetical protein X975 21286 partial	custom

Supplemental Table 3 – continued from previous page

Arthropod Core ID	Spider Core ID	Putative Ortholog Description	Target Database
NA	1164	CDP-diacylglycerol–glycerol-3-phosphate 3-phosphatidyltransferase mitochondrial partial	custom
NA	1165	Zinc transporter 1 partial	custom
NA	1166	Ectonucleoside triphosphate diphosphohydrolase 5 partial	custom
NA	1168	Proton-coupled amino acid transporter 1 partial	custom
NA	1171	Arylsulfatase B partial	custom
NA	1172	Mitogen-activated protein kinase-binding protein 1 partial	custom
NA	1179	Protein scribble-like protein partial	custom
NA	1180	Transforming growth factor-beta receptor-associated protein 1 partial	custom
NA	1181	hypothetical protein X975 16365 partial	custom
NA	1182	Equilibrative nucleoside transporter 3 partial	custom
NA	1184	F-box/WD repeat-containing protein 5 partial	custom
NA	1196	Sodium-coupled monocarboxylate transporter 1 partial	custom
NA	1197	Serine/threonine-protein kinase PINK1 mitochondrial partial	custom
NA	1198	hypothetical protein X975 26123 partial	custom
NA	1199	Beta-hexosaminidase subunit beta partial	custom
NA	1201	Myelin expression factor 2 partial	custom

Supplemental Table 3 – continued from previous page

Arthropod Core ID	Spider Core ID	Putative Ortholog Description	Target Database
90633	1202	Plasminogen activator inhibitor 1 RNA-binding protein partial	custom
NA	1203	Survival of motor neuron-related-splicing factor 30 partial	custom
NA	1204	hypothetical protein X975 05678 partial	custom
NA	1205	HBS1-like protein partial	custom
NA	1210	EH domain-containing protein 1 partial	custom
89726	1211	putative ferric-chelate reductase 1-like protein partial	custom
90041	1212	Multiple C2 and transmembrane domain-containing protein 2 partial	custom
NA	1213	Rab-like protein 1 partial	custom
NA	1215	putative cation-transporting ATPase 13A1 partial	custom
NA	1218	V-type proton ATPase subunit H partial	custom
NA	1219	hypothetical protein X975 12591 partial	custom
NA	1220	Phosphotidylinositol phosphatase PTPRQ partial	custom
NA	1221	Protein SET partial	custom
NA	1222	Tyrosine-protein phosphatase Lar partial	custom
NA	1223	Septin-2 partial	custom
NA	1225	Galactosylceramide sulfotransferase partial	custom
NA	1226	Microtubule-associated protein 2 partial	custom

Supplemental Table 3 – continued from previous page

Arthropod Core ID	Spider Core ID	Putative Ortholog Description	Target Database
NA	1227	Ubiquitin carboxyl-terminal hydrolase 4 partial	custom
NA	1230	Spectrin beta chain partial	custom
NA	1231	Fasciclin-2 partial	custom
NA	1233	Endothelin-converting enzyme 2 partial	custom
90493	1234	Zinc transporter 9 partial	custom
NA	1240	Coiled-coil and C2 domain-containing protein 1-like protein partial	custom
NA	1241	Acyl-CoA-binding domain-containing protein 5-B partial	custom
NA	1242	Membralin partial	custom
NA	1244	hypothetical protein X975 02454 partial	custom
NA	1245	Cullin-2 partial	custom
NA	1247	putative sodium-coupled neutral amino acid transporter 10 partial	custom
90080	1248	N-alpha-acetyltransferase 25 NatB auxiliary subunit partial	custom
NA	1249	Sodium/hydrogen exchanger 8 partial	custom
NA	1252	Major vault protein partial	custom
90152	1255	Nicalin-1 partial	custom
NA	1257	NA	nr
NA	1258	Kinesin light chain partial	custom
NA	1260	Rabenosyn-5 partial	custom
90518	1261	Cohesin subunit SA-1 partial	custom
90087	1265	DNA-directed RNA polymerase III subunit RPC1 partial	custom

Supplemental Table 3 – continued from previous page

Arthropod Core ID	Spider Core ID	Putative Ortholog Description	Target Database
90575	1279	Serine/threonine-protein phosphatase 2A regulatory subunit A alpha isoform partial	custom
NA	1280	Dynamin-1-like protein partial	custom
NA	1281	Actin cytoplasmic partial	custom
NA	1282	hypothetical protein X975 09546 partial	custom
NA	1283	Sushi von Willebrand factor type A EGF and pentraxin domain-containing protein 1 partial	custom
NA	1284	Cytosolic Fe-S cluster assembly factor nubp1 partial	custom
NA	1285	Alpha-aminoadipic semialdehyde dehydrogenase partial	custom
89794	1286	Signal recognition particle protein partial	custom
NA	1287	Paxillin partial	custom
NA	1289	hypothetical protein X975 17880 partial	custom
NA	1291	Protein sprint partial	custom
90113	1292	Myelin expression factor 2 partial	custom
NA	1293	Erlin-2-B partial	custom
NA	1294	Cold shock domain-containing protein E1 partial	custom
NA	1295	Alkaline phosphatase tissue-nonspecific isozyme partial	custom
NA	1297	hypothetical protein X975 04361 partial	custom
NA	1298	WD repeat and SOCS box-containing protein 1 partial	custom
NA	1299	hypothetical protein X975 11777 partial	custom
NA	1302	DNA-binding protein D-ETS-4 partial	custom

Supplemental Table 3 – continued from previous page

Arthropod Core ID	Spider Core ID	Putative Ortholog Description	Target Database
NA	1303	Integrin-linked kinase-associated serine/threonine phosphatase 2C partial	custom
NA	1304	NADPH–cytochrome P450 reductase partial	custom
NA	1305	Myosin light chain kinase smooth muscle partial	custom
89960	1310	mRNA export factor partial	custom
NA	1312	6-phosphogluconate dehydrogenase decarboxylating partial	custom
NA	1313	Multiple PDZ domain protein partial	custom
NA	1314	Hemocytin partial	custom
NA	1316	Oligopeptidase A partial	custom
NA	1317	hypothetical protein X975 14403 partial	custom
NA	1318	Prestin partial	custom
NA	1320	Cysteine desulfurase mitochondrial partial	custom
90460	1323	Nuclear protein localization protein 4-like protein partial	custom
90319	1326	Aspartate–tRNA ligase cytoplasmic partial	custom
NA	1327	Mitochondrial inner membrane protein partial	custom
90138	1328	hypothetical protein X975 03952 partial	custom
NA	1329	Mitochondrial uncoupling protein 2 partial	custom
NA	1331	Ubiquitin carboxyl-terminal hydrolase 5 partial	custom
NA	1335	hypothetical protein X975 00748 partial	custom
NA	1338	Exocyst complex component 3 partial	custom

Supplemental Table 3 – continued from previous page

Arthropod Core ID	Spider Core ID	Putative Ortholog Description	Target Database
NA	1339	hypothetical protein X975 23947 partial	custom
89721	1340	Structural maintenance of chromosomes protein 1A partial	custom
NA	1344	F-box only protein 7 partial	custom
NA	1347	Dual oxidase maturation factor 1 partial	custom
NA	1348	THO complex subunit 2 partial	custom
NA	1354	hypothetical protein X975 09429 partial	custom
NA	1356	Structural maintenance of chromosomes protein 4 partial	custom
NA	1357	hypothetical protein X975 03670 partial	custom
NA	1359	Egl nine-like protein partial	custom
NA	1360	Transmembrane protein 184B partial	custom
NA	1361	Activating signal cointegrator 1 complex subunit 2 partial	custom
NA	1362	Guanosine-3'5'-bis(diphosphate) 3'-pyrophosphohydrolase MESH1 partial	custom
NA	1363	Sorting nexin-25 partial	custom
NA	1364	Mediator of RNA polymerase II transcription subunit 17 partial	custom
NA	1365	Peroxisomal sarcosine oxidase partial	custom
NA	1367	Ankyrin-2 partial	custom
NA	1369	Vacuolar protein sorting-associated protein 11-like protein partial	custom
NA	1371	Epidermal growth factor receptor partial	custom

Supplemental Table 3 – continued from previous page

Arthropod Core ID	Spider Core ID	Putative Ortholog Description	Target Database
NA	1374	Guanine nucleotide-binding protein G(s) subunit alpha isoform partial	custom
NA	1375	Zinc finger protein 26 partial	custom
NA	1376	hypothetical protein X975 05252 partial	custom
NA	1378	Ribosomal protein S6 kinase 2 alpha partial	custom
NA	1379	Symplekin partial	custom
NA	1380	Nuclear pore complex protein Nup205 partial	custom
NA	1382	GRIP1-associated protein 1 partial	custom
90332	1391	N-terminal kinase-like protein partial	custom
NA	1392	Lamin Dm0 partial	custom
NA	1393	Phosphoserine phosphatase partial	custom
NA	1394	Mitochondrial Rho GTPase 1 partial	custom
NA	1396	Radixin partial	custom
NA	1397	Selenide water dikinase partial	custom
NA	1398	ATPase family AAA domain-containing protein 3 partial	custom
NA	1402	Vacuolar protein sorting-associated protein 18-like protein partial	custom
90617	1403	REST corepressor 3 partial	custom
NA	1404	Zinc finger protein rotund partial	custom
NA	1406	hypothetical protein X975 01518 partial	custom
90188	1407	putative rRNA-processing protein EBP2 partial	custom
NA	1408	Protein-L-isoaspartate(D-aspartate) O-methyltransferase partial	custom

Supplemental Table 3 – continued from previous page

Arthropod Core ID	Spider Core ID	Putative Ortholog Description	Target Database
NA	1412	Eukaryotic translation initiation factor 3 subunit D partial	custom
NA	1413	PI-PLC X domain-containing protein 3 partial	custom
NA	1415	Stromal interaction molecule-like protein partial	custom
NA	1416	Vesicle-fusing ATPase 1 partial	custom
NA	1417	Hsp90 co-chaperone Cdc37 partial	custom
89894	1418	Ras-related protein Rab-5C partial	custom
NA	1419	Protein Red partial	custom
NA	1420	Iron/zinc purple acid phosphatase-like protein partial	custom
NA	1421	hypothetical protein X975 05682 partial	custom
NA	1422	Dolichyl-diphosphooligosaccharide–protein glycosyltransferase subunit 2 partial	custom
90586	1424	Nucleolar protein 56 partial	custom
NA	1425	hypothetical protein X975 11637 partial	custom
89929	1426	Glutamate dehydrogenase mitochondrial partial	custom
NA	1427	Protein IWS1-like protein partial	custom
NA	1428	Prolyl endopeptidase partial	custom
NA	1429	putative glutamine–tRNA ligase partial	custom
NA	1431	Monocarboxylate transporter 9 partial	custom
NA	1435	FYVE RhoGEF and PH domain-containing protein 6 partial	custom
NA	1437	Serine/threonine-protein kinase TBK1 partial	custom

Supplemental Table 3 – continued from previous page

Arthropod Core ID	Spider Core ID	Putative Ortholog Description	Target Database
NA	1438	Protein Smaug-like protein partial	custom
NA	1440	Mitochondrial import inner membrane translocase subunit TIM50 partial	custom
NA	1441	hypothetical protein X975 19160 partial	custom
NA	1442	SWI/SNF-related matrix-associated actin-dependent regulator of chromatin subfamily B member 1 partial	custom
NA	1443	Brain-specific angiogenesis inhibitor 1-associated protein 2 partial	custom
NA	1444	Mediator of RNA polymerase II transcription subunit 16 partial	custom
NA	1445	Translocation protein SEC63-like protein partial	custom
NA	1449	Dual oxidase partial	custom
NA	1450	Zinc finger protein 143 partial	custom
NA	1451	DnaJ-like protein subfamily C member 16 partial	custom
NA	1452	Cyclin-D-binding Myb-like transcription factor 1 partial	custom
NA	1456	Solute carrier family 15 member 2 partial	custom
NA	1458	COP9 signalosome complex subunit 1 partial	custom
NA	1462	hypothetical protein X975 13615 partial	custom
NA	1465	TGF-beta receptor type-1 partial	custom
NA	1470	Cleavage and polyadenylation specificity factor subunit 2 partial	custom
NA	1474	Endophilin-B1 partial	custom

Supplemental Table 3 – continued from previous page

Arthropod Core ID	Spider Core ID	Putative Ortholog Description	Target Database
NA	1476	Conserved oligomeric Golgi complex subunit 6 partial	custom
NA	1477	Terminal uridylyltransferase 4 partial	custom
NA	1481	Hairy/enhancer-of-split related with YRPW motif protein 1 partial	custom
NA	1482	Rho GTPase-activating protein 39 partial	custom
NA	1487	Microtubule-associated protein futsch partial	custom
90502	1493	Electron transfer flavoprotein-ubiquinone oxidoreductase mitochondrial partial	custom
NA	1495	Calsequestrin-2 partial	custom
90009	1496	Caspase-1 partial	custom
NA	1498	Glycogenin-2 partial	custom
NA	1500	UV excision repair protein RAD23-like protein partial	custom
NA	1501	Yorkie-like protein partial	custom
NA	1502	hypothetical protein X975 11550 partial	custom
NA	1504	hypothetical protein X975 08113 partial	custom
NA	1505	Caprin-1 partial	custom
NA	1506	AT-rich interactive domain-containing protein 4B partial	custom
NA	1507	Mitochondrial import receptor subunit TOM70 partial	custom
89831	1508	V-type proton ATPase catalytic subunit A partial	custom

Supplemental Table 3 – continued from previous page

Arthropod Core ID	Spider Core ID	Putative Ortholog Description	Target Database
NA	1509	S-adenosylmethionine decarboxylase proenzyme partial	custom
NA	1510	ATP-dependent zinc metalloprotease YME1-like protein partial	custom
NA	1513	Rab11 family-interacting protein 4A partial	custom
NA	1515	Ocellar opsin partial	custom
NA	1516	Pleckstrin domain-containing family G member 1 partial	custom
NA	1518	WD repeat-containing protein 43 partial	custom
NA	1519	Src substrate protein p85 partial	custom
NA	1520	Ran-binding protein 3 partial	custom
NA	1522	Peroxisomal membrane protein PEX13 partial	custom
NA	1523	PX domain-containing protein kinase-like protein partial	custom
NA	1525	Intron-binding protein aquarius partial	custom
NA	1527	Sorting nexin-32 partial	custom
NA	1528	putative rRNA methyltransferase 3 partial	custom
NA	1529	Transmembrane protein 184C partial	custom
NA	1531	Nuclear factor related to kappa-B-binding protein partial	custom
NA	1532	Protein PRRC1 partial	custom
90643	1533	Homeobox protein extradenticle partial	custom
NA	1534	Elongator complex protein 3 partial	custom

Supplemental Table 3 – continued from previous page

Arthropod Core ID	Spider Core ID	Putative Ortholog Description	Target Database
NA	1535	X-ray repair cross-complementing protein 5 partial	custom
NA	1536	hypothetical protein X975 21837 partial	custom
NA	1537	SH3 domain-binding glutamic acid-rich protein-like protein partial	custom
NA	1539	Coproporphyrinogen-III oxidase partial	custom
90417	1540	putative protein-like protein partial	custom
NA	1542	Renin receptor partial	custom
NA	1543	Serine/threonine-protein kinase PRP4-like protein partial	custom
90249	1545	Minor histocompatibility antigen H13 partial	custom
NA	1546	LIM domain kinase 1 partial	custom
NA	1547	Monocarboxylate transporter 10 partial	custom
NA	1548	Aldo-keto reductase family 1 member B10 partial	custom
NA	1552	Dual specificity mitogen-activated protein kinase kinase 1 partial	custom
NA	1554	Transmembrane 9 superfamily member 2 partial	custom
NA	1555	Trans-2-enoyl-CoA reductase mitochondrial partial	custom
NA	1556	Aspartate aminotransferase cytoplasmic partial	custom
NA	1557	Thioredoxin reductase 1 cytoplasmic partial	custom
NA	1558	Beta-amyloid-like protein partial	custom
NA	1559	hypothetical protein X975 17569 partial	custom

Supplemental Table 3 – continued from previous page

Arthropod Core ID	Spider Core ID	Putative Ortholog Description	Target Database
90656	1560	heat shock protein mitochondrial partial	custom
NA	1561	Glucose-6-phosphate 1-dehydrogenase partial	custom
NA	1563	Serum response factor-binding protein 1 partial	custom
NA	1564	2-amino-3-ketobutyrate coenzyme A ligase mitochondrial partial	custom
90085	1565	RuvB-like 2 partial	custom
NA	1567	Phosphoribosyl pyrophosphate synthase-associated protein 2 partial	custom
90626	1568	Mitochondrial 2-oxoglutarate/malate carrier protein partial	custom
NA	1572	Protein HEXIM partial	custom
NA	1573	Succinyl-CoA ligase [ADP-forming] subunit beta mitochondrial partial	custom
NA	1574	Importin-4 partial	custom
NA	1576	hypothetical protein X975 01772 partial	custom
NA	1577	Techylectin-5B partial	custom
NA	1578	Periostin partial	custom
90424	1579	Dolichyl-diphosphooligosaccharide–protein glycosyltransferase subunit partial	custom
NA	1580	Peptidyl-prolyl cis-trans isomerase FKBP4 partial	custom
NA	1581	hypothetical protein X975 06760 partial	custom
NA	1582	hypothetical protein X975 25386 partial	custom
90059	1583	T-complex protein 1 subunit beta partial	custom
NA	1584	Trifunctional enzyme subunit alpha mitochondrial partial	custom

Supplemental Table 3 – continued from previous page

Arthropod Core ID	Spider Core ID	Putative Ortholog Description	Target Database
NA	1585	Tetratricopeptide repeat protein 4 partial	custom
NA	1587	Pumilio domain-containing protein partial	custom
90482	1588	DnaJ-like protein subfamily C member 2 partial	custom
NA	1589	DET1- and DDB1-associated protein 1 partial	custom
NA	1590	DNA damage-regulated autophagy modulator protein 1 partial	custom
NA	1591	mRNA-capping enzyme partial	custom
NA	1592	Pyruvate carboxylase mitochondrial partial	custom
NA	1596	Sphingomyelin phosphodiesterase D LrSicTox-alphaIB1 partial	custom
NA	1597	hypothetical protein X975 05837 partial	custom
NA	1598	Calpain-7 partial	custom
NA	1601	Vesicle-associated membrane protein 7 partial	custom
89906	1602	Peroxisome assembly factor 2 partial	custom
NA	1606	Zinc finger protein 768 partial	custom
89870	1609	Integrator complex subunit 6-A partial	custom
NA	1610	Exocyst complex component 4 partial	custom
NA	1611	Acidic mammalian chitinase partial	custom
NA	1612	Leucine-rich repeat and calponin domain-containing protein 1 partial	custom
NA	1613	Otopetrin-2 partial	custom
NA	1614	Serine/threonine-protein kinase pelle partial	custom

Supplemental Table 3 – continued from previous page

Arthropod Core ID	Spider Core ID	Putative Ortholog Description	Target Database
NA	1617	Replication protein A subunit partial	custom
NA	1620	hypothetical protein X975 06323 partial	custom
NA	1622	Maltase-glucoamylase intestinal partial	custom
NA	1627	Excitatory amino acid transporter 3 partial	custom
NA	1629	F-box only protein 11 partial	custom
NA	1630	Ankyrin repeat and FYVE domain-containing protein 1 partial	custom
NA	1631	Protein osteopotentia-like protein partial	custom
90231	1633	E3 ubiquitin-protein ligase MARCH6 partial	custom
NA	1646	Serine/threonine-protein phosphatase PP1-gamma catalytic subunit partial	custom
NA	1649	Retinoid isomerohydrolase partial	custom
NA	1650	Rho GTPase-activating protein 28 partial	custom
NA	1651	Protein phosphatase 1 regulatory subunit 12A partial	custom
NA	1652	hypothetical protein X975 01699 partial	custom
NA	1653	ATP-binding cassette sub-family F member 2 partial	custom
90207	1654	AP-1 complex subunit gamma-1 partial	custom
NA	1656	putative ATP-dependent RNA helicase DDX47 partial	custom
NA	1657	hypothetical protein X975 25774 partial	custom

Supplemental Table 3 – continued from previous page

Arthropod Core ID	Spider Core ID	Putative Ortholog Description	Target Database
NA	1658	Cell division cycle protein 27-like protein partial	custom
NA	1659	Actin cytoplasmic partial	custom
NA	1660	hypothetical protein X975 24063 partial	custom
NA	1661	Eukaryotic translation initiation factor 5 partial	custom
89662	1662	Far upstream element-binding protein 1 partial	custom
NA	1663	Cysteine string protein partial	custom
NA	1664	Protein toll partial	custom
NA	1665	NF-X1-type zinc finger protein NFXL1 partial	custom
NA	1668	Glycerol-3-phosphate acyltransferase 4 partial	custom
NA	1669	hypothetical protein X975 25298 partial	custom
89877	1670	Regulator of nonsense transcripts 2 partial	custom
NA	1671	Scavenger receptor class B member 1 partial	custom
90245	1673	Nucleolar GTP-binding protein 2 partial	custom
NA	1674	Dynactin subunit 1 partial	custom
90291	1675	Cleavage and polyadenylation specificity factor subunit 6 partial	custom
90400	1676	V-type proton ATPase subunit d 1 partial	custom
NA	1678	hypothetical protein X975 18972 partial	custom
NA	1679	Notchless protein-like protein partial	custom

Supplemental Table 3 – continued from previous page

Arthropod Core ID	Spider Core ID	Putative Ortholog Description	Target Database
NA	1680	Enhancer of mRNA-decapping protein 4 partial	custom
NA	1681	Nuclear pore complex protein Nup107 partial	custom
NA	1682	Inositol monophosphatase 2 partial	custom
NA	1683	putative sulfite oxidase mitochondrial partial	custom
NA	1684	NSFL1 cofactor p47 partial	custom
NA	1685	Glucosidase 2 subunit beta partial	custom
NA	1686	Glycogenin-1 partial	custom
NA	1687	26S proteasome non-ATPase regulatory subunit 14 partial	custom
NA	1688	Amyloid protein-binding protein 2 partial	custom
NA	1689	Cytosolic carboxypeptidase 1 partial	custom
89947	1690	2-oxoisovalerate dehydrogenase subunit beta mitochondrial partial	custom
NA	1692	hypothetical protein X975 22465 partial	custom
NA	1693	B(0+)-type amino acid transporter 1 partial	custom
90137	1694	Protein disulfide-isomerase A6 partial	custom
90455	1696	Nucleolar protein 16 partial	custom
90309	1698	Phosphoserine phosphatase partial	custom
89683	1699	Calnexin partial	custom
NA	1701	ATP-binding cassette sub-family F member 3 partial	custom
90296	1702	26S proteasome non-ATPase regulatory subunit 12 partial	custom

Supplemental Table 3 – continued from previous page

Arthropod Core ID	Spider Core ID	Putative Ortholog Description	Target Database
90619	1703	Zygotic DNA replication licensing factor mcm6-B partial	custom
90020	1704	RuvB-like 1 partial	custom
NA	1705	hypothetical protein X975 13285 partial	custom
NA	1707	Transforming growth factor-beta-induced protein ig-h3 partial	custom
NA	1709	Actin-related protein 2 partial	custom
NA	1710	AP-2 complex subunit mu partial	custom
NA	1711	Inositol-trisphosphate 3-kinase B partial	custom
NA	1712	hypothetical protein X975 06095 partial	custom
NA	1713	Alpha-ketoglutarate-dependent dioxygenase alkB-like protein partial	custom
NA	1715	Serine/threonine-protein kinase pim-3 partial	custom
NA	1718	hypothetical protein X975 14727 partial	custom
NA	1719	Serine/threonine-protein kinase RIO3 partial	custom
90106	1720	U4/U6 small nuclear ribonucleoprotein Prp31 partial	custom
89783	1722	T-complex protein 1 subunit eta partial	custom
89916	1723	Coatomer subunit delta partial	custom
NA	1724	ATPase family AAA domain-containing protein 1-B partial	custom
NA	1725	PTB domain-containing engulfment adapter protein 1 partial	custom
NA	1726	hypothetical protein X975 15128 partial	custom
NA	1727	hypothetical protein X975 10062 partial	custom

Supplemental Table 3 – continued from previous page

Arthropod Core ID	Spider Core ID	Putative Ortholog Description	Target Database
89850	1729	Importin-5 partial	custom
NA	1730	hypothetical protein X975 18273 partial	custom
NA	1731	Lysine–tRNA ligase partial	custom
NA	1732	Early growth response protein 1 partial	custom
90590	1733	tRNA pseudouridine synthase A mitochondrial partial	custom
89738	1734	DnaJ-like protein subfamily C member 3 partial	custom
89746	1735	WD repeat-containing protein 7 partial	custom
NA	1736	Presequence protease mitochondrial partial	custom
89961	1737	39S ribosomal protein L22 mitochondrial partial	custom
NA	1739	Translation initiation factor eIF-2B subunit epsilon partial	custom
NA	1740	Eukaryotic translation initiation factor 3 subunit E-A partial	custom
90058	1741	PREDICTED:pescadillo-like	nr
NA	1743	BSD domain-containing protein 1 partial	custom
NA	1744	Angiomotin partial	custom
NA	1746	Lateral signaling target protein 2-like protein partial	custom
90368	1747	hypothetical protein X975 18921 partial	custom
NA	1749	Neutral alpha-glucosidase AB partial	custom
NA	1750	hypothetical protein X975 25520 partial	custom
NA	1751	Conserved oligomeric Golgi complex subunit 1 partial	custom

Supplemental Table 3 – continued from previous page

Arthropod Core ID	Spider Core ID	Putative Ortholog Description	Target Database
89754	1752	Serine/threonine-protein kinase RIO2 partial	custom
NA	1756	MOB kinase activator-like 2 partial	custom
NA	1760	Nesprin-1 partial	custom
89958	1761	Pre-mRNA-processing factor 6 partial	custom
NA	1762	Nuclear hormone receptor HR96 partial	custom
NA	1764	Leucine-rich repeats and immunoglobulin-like domains protein 3 partial	custom
NA	1767	FAST kinase domain-containing protein 2 partial	custom
NA	1768	Splicing factor suppressor of white-apricot-like protein partial	custom
NA	1769	hypothetical protein X975 14597 partial	custom
NA	1771	hypothetical protein X975 06629 partial	custom
90429	1774	1-phosphatidylinositol-3-phosphate 5-kinase partial	custom
NA	1775	Ubiquitin carboxyl-terminal hydrolase 10 partial	custom
NA	1778	Leucine-rich repeat protein 1 partial	custom
NA	1781	Epithelial chloride channel protein partial	custom
NA	1783	Protein phosphatase 1 regulatory subunit 14B partial	custom
NA	1784	PREDICTED:syntaxin-17-like	nr
90177	1785	Cap-specific mRNA (nucleoside-2'-O-)-methyltransferase 1 partial	custom

Supplemental Table 3 – continued from previous page

Arthropod Core ID	Spider Core ID	Putative Ortholog Description	Target Database
NA	1787	Anaphase-promoting complex subunit 2 partial	custom
NA	1789	Histone deacetylase 6 partial	custom
89823	1790	Phosphatidylserine decarboxylase proenzyme partial	custom
NA	1791	Major facilitator superfamily domain-containing protein 1 partial	custom
NA	1792	hypothetical protein X975 10476 partial	custom
NA	1795	Rab proteins geranyl-geranyltransferase component A 1 partial	custom
NA	1797	Fat-like cadherin-related tumor suppressor-like protein partial	custom
NA	1798	Fidgetin-like protein 1 partial	custom
89728	1800	Transportin-1 partial	custom
90576	1806	ATP-binding cassette sub-family D member 2 partial	custom
NA	1808	hypothetical protein X975 03993 partial	custom
NA	1812	Phosphofurin acidic cluster sorting protein 2 partial	custom
90610	1814	Lysine-specific demethylase 6A partial	custom
NA	1822	PREDICTED:Golgi-associated PDZ and coiled-coil motif-containing protein-like	nr
NA	1824	Heterogeneous nuclear ribonucleo-protein H partial	custom
NA	1825	H/ACA ribonucleo-protein complex sub-unit 4 partial	custom
NA	1827	NA	nr

Supplemental Table 3 – continued from previous page

Arthropod Core ID	Spider Core ID	Putative Ortholog Description	Target Database
89952	1828	tRNA-dihydrouridine(20) synthase [NAD(P)+]-like protein partial	custom
NA	1829	14-3-3 protein epsilon partial	custom
90278	1830	Double-stranded RNA-specific adenosine deaminase partial	custom
NA	1831	Mitochondrial glutamate carrier 1 partial	custom
NA	1832	Dimethylglycine dehydrogenase mitochondrial partial	custom
NA	1833	Hormone-sensitive lipase partial	custom
NA	1834	UBX domain-containing protein 4	nr
NA	1835	Nucleosome assembly protein 1-like 4 partial	custom
NA	1836	Protein bric-a-brac 1 partial	custom
NA	1837	hypothetical protein X975 09406 partial	custom
NA	1838	Dehydrodolichyl diphosphate synthase partial	custom
NA	1841	SRSF protein kinase 1 partial	custom
NA	1842	E3 ubiquitin-protein ligase KCMF1 partial	custom
NA	1843	hypothetical protein X975 08235 partial	custom
90209	1844	Splicing factor U2AF subunit partial	custom
89674	1845	2-hydroxyacyl-CoA lyase 1 partial	custom
NA	1846	Sorting nexin-2 partial	custom
NA	1848	Clusterin-associated protein 1-like protein partial	custom
89676	1849	Tripeptidyl-peptidase 2 partial	custom

Supplemental Table 3 – continued from previous page

Arthropod Core ID	Spider Core ID	Putative Ortholog Description	Target Database
NA	1850	Vascular endothelial growth factor receptor 3 partial	custom
NA	1851	Protein FAM192A partial	custom
NA	1852	Calmodulin partial	custom
NA	1853	Transducin beta-like protein 3 partial	custom
NA	1854	Transcription factor Sox-6 partial	custom
NA	1855	Microfibrillar-associated protein 1 partial	custom
NA	1857	putative ribosome biogenesis protein partial	custom
NA	1858	Enoyl-CoA delta isomerase 2 mitochondrial partial	custom
NA	1859	Protein canopy 4 partial	custom
NA	1860	Carnitine O-acetyltransferase partial	custom
NA	1861	putative isocitrate dehydrogenase [NAD] subunit alpha mitochondrial partial	custom
NA	1862	Folliculin partial	custom
NA	1864	26S proteasome non-ATPase regulatory subunit 7 partial	custom
NA	1866	14-alpha-glucan-branching enzyme partial	custom
NA	1868	ELAV-like protein 2 partial	custom
NA	1870	RAC serine/threonine-protein kinase partial	custom
90227	1873	26S proteasome non-ATPase regulatory subunit 11 partial	custom
NA	1874	Delta-aminolevulinic acid dehydratase partial	custom
NA	1876	putative oxidoreductase partial	custom

Supplemental Table 3 – continued from previous page

Arthropod Core ID	Spider Core ID	Putative Ortholog Description	Target Database
NA	1879	Tryptophan–tRNA ligase cytoplasmic partial	custom
89979	1880	5'-AMP-activated protein kinase catalytic subunit alpha-2 partial	custom
NA	1882	Integrin-linked protein kinase partial	custom
NA	1883	Methylmalonate-semialdehyde dehydrogenase [acylating] mitochondrial partial	custom
90597	1885	26S protease regulatory subunit 7 partial	custom
NA	1886	Thioredoxin domain-containing protein 5 partial	custom
NA	1887	putative serpin-like protein partial	custom
NA	1888	Heterogeneous nuclear ribonucleoprotein F partial	custom
NA	1890	Polyadenylate-binding protein-interacting protein 1 partial	custom
NA	1891	Alanine–tRNA ligase cytoplasmic partial	custom
NA	1892	Adenylosuccinate synthetase partial	custom
NA	1893	SH2 domain-containing protein 3C partial	custom
89949	1894	Pre-mRNA-splicing factor SLU7 partial	custom
89821	1895	Inorganic pyrophosphatase partial	custom
90535	1897	26S proteasome non-ATPase regulatory subunit 2 partial	custom
NA	1901	hypothetical protein X975 01182 partial	custom
NA	1902	Exocyst complex component 7 partial	custom
NA	1903	WASH complex subunit 7 partial	custom

Supplemental Table 3 – continued from previous page

Arthropod Core ID	Spider Core ID	Putative Ortholog Description	Target Database
NA	1906	Organic cation transporter protein partial	custom
NA	1907	hypothetical protein X975 11906 partial	custom
NA	1908	Transmembrane protein 87A partial	custom
NA	1911	Protein ROP partial	custom
NA	1912	BTB/POZ domain-containing adapter for CUL3-mediated RhoA degradation protein 3 partial	custom
NA	1913	N-acetyllactosaminide beta-13-N-acetylglucosaminyltransferase partial	custom
NA	1915	Mucolipin-3 partial	custom
NA	1916	hypothetical protein X975 02383 partial	custom
NA	1918	NF-kappa-B inhibitor cactus partial	custom
NA	1919	N-acetylglucosamine-1-phosphotransferase subunit gamma partial	custom
NA	1921	N-acetyltransferase 9-like protein partial	custom
NA	1922	Carbonic anhydrase 2 partial	custom
NA	1924	RING finger protein nhl-1 partial	custom
NA	1925	CBM21 domain-containing protein partial	custom
89732	1927	N-acetylglucosaminyl-phosphatidylinositol biosynthetic protein partial	custom
NA	1928	WD repeat-containing protein 11 partial	custom
NA	1930	putative methyltransferase NSUN4 partial	custom

Supplemental Table 3 – continued from previous page

Arthropod Core ID	Spider Core ID	Putative Ortholog Description	Target Database
NA	1931	3-hydroxyanthranilate 34-dioxygenase partial	custom
NA	1932	Splicing factor 3A subunit 2 partial	custom
NA	1933	Myotubularin-related protein 8 partial	custom
NA	1934	TNF receptor-associated factor 6 partial	custom
NA	1935	Myotubularin-related protein 9 partial	custom
NA	1936	Lysine-specific histone demethylase 1A partial	custom
NA	1937	Pre-mRNA-splicing regulator female-lethal(2)D partial	custom
NA	1939	Chromatin accessibility complex protein 1 partial	custom
NA	1940	Dipeptidyl peptidase 9 partial	custom
NA	1946	Cleft lip and palate transmembrane protein 1-like protein partial	custom
NA	1949	GC-rich sequence DNA-binding factor 1 partial	custom
NA	1951	hypothetical protein X975 00693 partial	custom
NA	1956	Kelch domain-containing protein 4 partial	custom
NA	1958	Iduronate 2-sulfatase partial	custom
NA	1959	Protein bicaudal D partial	custom
NA	1960	Pro-interleukin-16 partial	custom
NA	1961	hypothetical protein X975 10251 partial	custom
NA	1962	Inosine-5'-monophosphate dehydrogenase 1 partial	custom

Supplemental Table 3 – continued from previous page

Arthropod Core ID	Spider Core ID	Putative Ortholog Description	Target Database
90477	1964	Protein phosphatase PTC7-like protein partial	custom
NA	1966	WD repeat-containing protein 75 partial	custom
NA	1967	hypothetical protein X975 13894 partial	custom
89687	1969	DNA topoisomerase 3-beta-1 partial	custom
NA	1971	tRNA-dihydrouridine(47) synthase [NAD(P)(+)]-like protein partial	custom
NA	1980	F-box/LRR-repeat protein 2 partial	custom
NA	1981	Ubiquilin-1 partial	custom
NA	1982	Protein SDA1-like protein partial	custom
90553	1984	hypothetical protein X975 24184 partial	custom
NA	1986	Polypyrimidine tract-binding protein 2 partial	custom
NA	1988	Tyrosine-protein kinase transmembrane receptor ROR1 partial	custom
NA	1989	hypothetical protein X975 10634 partial	custom
NA	1990	NFX1-type zinc finger-containing protein 1 partial	custom
NA	1991	E3 ubiquitin-protein ligase synoviolin B partial	custom
NA	1992	hypothetical protein X975 25549 partial	custom
NA	1993	hypothetical protein X975 27039 partial	custom
NA	1994	60S ribosomal protein L3 partial	custom
NA	1995	L-asparaginase partial	custom
NA	1999	Ethanolamine-phosphate cytidylyltransferase partial	custom

Supplemental Table 3 – continued from previous page

Arthropod Core ID	Spider Core ID	Putative Ortholog Description	Target Database
NA	2000	Methylmalonic aciduria and homocystinuria type D protein mitochondrial partial	custom
NA	2001	Nostrin partial	custom
NA	2004	Mannose-1-phosphate guanyltransferase alpha-B partial	custom
NA	2005	Protein held out wing partial	custom
NA	2006	hypothetical protein X975 03273 partial	custom
NA	2007	putative lysine-specific demethylase 4B partial	custom
NA	2008	MIF4G domain-containing protein A partial	custom
NA	2009	Histidine triad nucleotide-binding protein 2 mitochondrial partial	custom
NA	2010	Nose resistant to fluoxetine protein 6 partial	custom
NA	2013	Inhibitor of nuclear factor kappa-B kinase subunit beta partial	custom
NA	2015	Pre-mRNA-splicing factor SYF1 partial	custom
NA	2016	Pre-rRNA-processing protein TSR1-like protein partial	custom
90376	2018	Methylthioribose-1-phosphate isomerase partial	custom
NA	2020	GAS2-like protein 1 partial	custom
NA	2022	DDRGK domain-containing protein 1 partial	custom
NA	2023	DDB1- and CUL4-associated factor 13 partial	custom
NA	2024	Sugar phosphate exchanger 3 partial	custom

Supplemental Table 3 – continued from previous page

Arthropod Core ID	Spider Core ID	Putative Ortholog Description	Target Database
89921	2025	hypothetical protein X975 13109 partial	custom
NA	2028	Williams-Beuren syndrome chromosomal region 16 protein-like protein partial	custom
NA	2030	hypothetical protein X975 08621 partial	custom
NA	2031	Transmembrane 9 superfamily member 3 partial	custom
89710	2032	V-type proton ATPase subunit B partial	custom
NA	2033	Steroidogenic acute regulatory protein mitochondrial partial	custom
NA	2034	Tubulointerstitial nephritis antigen-like protein partial	custom
NA	2036	HIV Tat-specific factor 1 partial	custom
NA	2037	Nucleoredoxin-like protein 2 partial	custom
89845	2038	Sideroflexin-1 partial	custom
NA	2040	COP9 signalosome complex subunit 2 partial	custom
90436	2041	Dihydrolipoyllysine-residue succinyltransferase component of 2-oxoglutarate dehydrogenase complex mitochondrial partial	custom
89999	2042	Lupus La protein partial	custom
NA	2043	hypothetical protein X975 05087 partial	custom
NA	2045	Lissencephaly-1-like protein partial	custom
NA	2046	Nucleolar protein 58 partial	custom
90375	2047	Delta-1-pyrroline-5-carboxylate dehydrogenase mitochondrial partial	custom
NA	2048	T-complex protein 1 subunit theta partial	custom

Supplemental Table 3 – continued from previous page

Arthropod Core ID	Spider Core ID	Putative Ortholog Description	Target Database
NA	2049	ATP-binding cassette sub-family E member 1 partial	custom
NA	2050	Protein disulfide-isomerase TMX3 partial	custom
NA	2053	Inhibin beta B chain partial	custom
NA	2054	RING finger protein 31 partial	custom
NA	2055	Protein Gawky partial	custom
89827	2056	Methionine aminopeptidase 2 partial	custom
NA	2057	Zinc transporter SLC39A7 partial	custom
NA	2059	Deoxyribonuclease-2 partial	custom
NA	2060	Protein ERGIC-53 partial	custom
NA	2062	hypothetical protein X975 23445 partial	custom
NA	2067	MAM domain-containing glycosylphosphatidylinositol anchor protein 1 partial	custom
NA	2069	Glutathione S-transferase theta-1 partial	custom
NA	2075	mRNA-decapping enzyme 2 partial	custom
NA	2078	Beta-13-glucosyltransferase partial	custom
NA	2079	CCAAT/enhancer-binding protein zeta partial	custom
90011	2080	putative ATP-dependent RNA helicase DDX27 partial	custom
NA	2081	Endoplasmic reticulum resident protein 44 partial	custom
NA	2082	Mitoferrin-2 partial	custom
NA	2086	Galactokinase partial	custom

Supplemental Table 3 – continued from previous page

Arthropod Core ID	Spider Core ID	Putative Ortholog Description	Target Database
89977	2088	Pyridoxal-dependent decarboxylase domain-containing protein 1 partial	custom
NA	2089	Protein AHNAK2 partial	custom
89771	2090	Splicing factor 3A subunit 3 partial	custom
NA	2092	Phenylalanine–tRNA ligase alpha subunit B partial	custom
NA	2096	Calcyphosin-like protein partial	custom
NA	2097	U1 small nuclear ribonucleoprotein C partial	custom
NA	2099	Coiled-coil domain-containing protein 22-like protein partial	custom
NA	2100	Transmembrane protein 214-B partial	custom
NA	2101	Eukaryotic elongation factor 2 kinase partial	custom
NA	2102	ATP-dependent RNA helicase SUV3-like protein mitochondrial partial	custom
NA	2103	Metallophosphoesterase 1 partial	custom
NA	2104	Carbonyl reductase [NADPH] 1 partial	custom
NA	2108	Abhydrolase domain-containing protein 4 partial	custom
NA	2109	Protein maelstrom-like protein partial	custom
NA	2110	U3 small nucleolar RNA-associated protein 15-like protein partial	custom
NA	2113	Transmembrane 9 superfamily member 2 partial	custom
NA	2114	hypothetical protein X975 09833 partial	custom

Supplemental Table 3 – continued from previous page

Arthropod Core ID	Spider Core ID	Putative Ortholog Description	Target Database
NA	2117	DNA mismatch repair protein Msh2 partial	custom
90172	2118	Kinesin-associated protein 3 partial	custom
NA	2119	hypothetical protein X975 25921 partial	custom
NA	2120	Bicaudal D-related protein-like protein partial	custom
NA	2121	Transmembrane protein partial	custom
NA	2122	Mitochondrial-processing peptidase subunit alpha partial	custom
NA	2123	Betabeta-carotene 1515'-monooxygenase partial	custom
NA	2125	putative ribosomal RNA methyltransferase NOP2 partial	custom
NA	2126	hypothetical protein X975 09051 partial	custom
NA	2127	Protein slowmo partial	custom
NA	2129	Group XV phospholipase A2 partial	custom
NA	2130	FAD-dependent oxidoreductase domain-containing protein 1 partial	custom
NA	2132	Dual specificity protein phosphatase 8 partial	custom
NA	2134	Ras-related protein Rab-10 partial	custom
NA	2135	FAS-associated factor 1 partial	custom
NA	2136	Acyl-CoA synthetase family member 3 mitochondrial partial	custom
90006	2139	Sorting nexin-27 partial	custom
NA	2142	hypothetical protein X975 05027 partial	custom
NA	2146	hypothetical protein X975 01440 partial	custom

Supplemental Table 3 – continued from previous page

Arthropod Core ID	Spider Core ID	Putative Ortholog Description	Target Database
90544	2149	Piwi-like protein 1 partial	custom
89840	2151	Splicing factor 3B subunit 2 partial	custom
NA	2153	Cysteine/serine-rich nuclear protein 3 partial	custom
90452	2154	Parafibromin partial	custom
NA	2157	Protein lap4 partial	custom
NA	2163	Regulator of G-protein signaling 12 partial	custom
NA	2164	TBC1 domain family member 14 partial	custom
89872	2165	Leucine-rich repeat protein soc-2-like protein partial	custom
NA	2166	tRNA wybutosine-synthesizing protein 1-like protein partial	custom
NA	2167	hypothetical protein X975 00044 partial	custom
NA	2178	Cytochrome P450 4V2 partial	custom
NA	2183	Zinc finger protein 622 partial	custom
NA	2184	Serine hydroxymethyltransferase mitochondrial partial	custom
NA	2185	ABC transporter G family member 23 partial	custom
NA	2187	Acidic leucine-rich nuclear phosphoprotein 32 family member A partial	custom
90325	2188	Inositol monophosphatase 3 partial	custom
90196	2189	26S proteasome non-ATPase regulatory subunit 4 partial	custom
NA	2190	Signal transducing adapter molecule 1 partial	custom
NA	2191	hypothetical protein X975 19620 partial	custom
NA	2193	ADP/ATP translocase 1 partial	custom

Supplemental Table 3 – continued from previous page

Arthropod Core ID	Spider Core ID	Putative Ortholog Description	Target Database
90184	2194	Trifunctional enzyme subunit beta mitochondrial partial	custom
NA	2195	Prolactin regulatory element-binding protein partial	custom
NA	2196	putative signal peptidase complex subunit 2 partial	custom
NA	2197	Nucleoporin p58/p45 partial	custom
NA	2198	26S proteasome non-ATPase regulatory subunit 6 partial	custom
NA	2201	Eukaryotic translation initiation factor 3 subunit M partial	custom
NA	2202	Dihydrolipoyllysine-residue acetyltransferase component of pyruvate dehydrogenase complex mitochondrial partial	custom
NA	2203	Suppressor of SWI4 1-like protein partial	custom
NA	2204	Integral membrane protein 2B partial	custom
NA	2205	Rho GTPase-activating protein 100F partial	custom
89971	2207	hypothetical protein X975 05150 partial	custom
NA	2209	Nitrogen permease regulator 3-like protein partial	custom
NA	2210	ADP-ribosylation factor GTPase-activating protein 1 partial	custom
NA	2213	3-hydroxybutyryl-CoA dehydrogenase partial	custom
NA	2214	Palmitoyltransferase ZDHHC2 partial	custom
NA	2215	3-hydroxyisobutyrate dehydrogenase mitochondrial partial	custom
NA	2216	hypothetical protein X975 05086 partial	custom

Supplemental Table 3 – continued from previous page

Arthropod Core ID	Spider Core ID	Putative Ortholog Description	Target Database
NA	2217	WD repeat domain phosphoinositide-interacting protein 2 partial	custom
90208	2219	hypothetical protein X975 16574 partial	custom
NA	2222	Protein SMG9 partial	custom
90194	2223	26S protease regulatory subunit 8 partial	custom
NA	2225	BAG domain-containing protein Samui partial	custom
NA	2227	Cytosolic Fe-S cluster assembly factor nubp1 partial	custom
NA	2228	hypothetical protein X975 05242 partial	custom
90560	2229	Peroxiredoxin 1 partial	custom
NA	2230	HEAT repeat-containing protein 3 partial	custom
NA	2233	Small glutamine-rich tetratricopeptide repeat-containing protein alpha partial	custom
NA	2234	Arginine–tRNA ligase cytoplasmic partial	custom
89874	2235	Transcription elongation factor S-II partial	custom
NA	2236	Suppressor of fused-like protein partial	custom
89873	2239	Thioredoxin-like protein 1 partial	custom
NA	2240	Replication protein A DNA-binding subunit partial	custom
90513	2241	NA	nr
NA	2243	Protein disulfide-isomerase A5 partial	custom
NA	2244	Paramyosin partial	custom
NA	2245	G-protein coupled receptor 126 partial	custom
NA	2246	DDB1- and CUL4-associated factor 8 partial	custom

Supplemental Table 3 – continued from previous page

Arthropod Core ID	Spider Core ID	Putative Ortholog Description	Target Database
NA	2247	BRCA1-associated protein partial	custom
NA	2248	Histone-binding protein RBBP4 partial	custom
90485	2253	NADH dehydrogenase [ubiquinone] 1 alpha subcomplex subunit 9 mitochondrial partial	custom
89788	2254	hypothetical protein X975 02868 partial	custom
NA	2255	Transcription initiation factor IIA subunit 1 partial	custom
90123	2256	Ornithine aminotransferase mitochondrial partial	custom
90243	2258	Cysteine–tRNA ligase cytoplasmic partial	custom
NA	2259	FAS-associated factor 2-B partial	custom
NA	2262	Bifunctional purine biosynthesis protein PURH partial	custom
NA	2263	Interferon regulatory factor 2-binding protein 2-B partial	custom
89833	2266	Catalase partial	custom
NA	2267	Nuclear factor interleukin-3-regulated protein partial	custom
NA	2268	NTF2-related export protein 2 partial	custom
NA	2269	S-phase kinase-associated protein 1 partial	custom
NA	2270	Dihydrolipoyl dehydrogenase mitochondrial partial	custom
90253	2271	Cdc42-like protein partial	custom
90088	2275	Signal recognition particle protein partial	custom
NA	2276	putative aminopeptidase partial	custom

Supplemental Table 3 – continued from previous page

Arthropod Core ID	Spider Core ID	Putative Ortholog Description	Target Database
NA	2277	Y+L amino acid transporter 2 partial	custom
NA	2279	Sushi von Willebrand factor type A EGF and pentraxin domain-containing protein 1 partial	custom
NA	2281	GRIP and coiled-coil domain-containing protein 1 partial	custom
NA	2283	Neutral ceramidase partial	custom
NA	2285	Vesicle transport through interaction with t-SNAREs-like protein 1B partial	custom
NA	2286	E3 ubiquitin-protein ligase MARCH5 partial	custom
NA	2287	Acidic fibroblast growth factor intracellular-binding protein partial	custom
NA	2289	Ran GTPase-activating protein 1 partial	custom
NA	2290	Methylosome subunit pICln partial	custom
NA	2291	Transcription initiation factor TFIID subunit 12 partial	custom
NA	2292	Zinc finger protein 407 partial	custom
NA	2293	Regulator of nonsense transcripts 3A partial	custom
NA	2294	RNA polymerase II-associated factor 1-like protein partial	custom
90395	2297	SPRY domain-containing protein 7 partial	custom
NA	2298	Stathmin-1-A partial	custom
NA	2299	U4/U6.U5 tri-snRNP-associated protein 2 partial	custom
NA	2303	hypothetical protein X975 16956 partial	custom

Supplemental Table 3 – continued from previous page

Arthropod Core ID	Spider Core ID	Putative Ortholog Description	Target Database
NA	2304	Aprataxin partial	custom
NA	2306	CWF19-like protein 2 partial	custom
NA	2307	BTB/POZ domain-containing protein 9 partial	custom
90567	2308	Transaldolase partial	custom
NA	2309	Josephin-1 partial	custom
NA	2310	CAAX prenyl protease 1-like protein partial	custom
NA	2312	Signal peptide peptidase-like 2B partial	custom
NA	2315	Cell division cycle protein 16-like protein partial	custom
90440	2317	Ras-related protein Rab-35 partial	custom
89848	2318	UBX domain-containing protein 6 partial	custom
NA	2319	Synapse-associated protein 1 partial	custom
NA	2322	RalA-binding protein 1 partial	custom
NA	2324	Golgi apparatus protein 1 partial	custom
NA	2325	Nitrilase and fragile histidine triad fusion protein NitFhit partial	custom
90587	2331	60S ribosomal protein L27a partial	custom
NA	2332	Suppressor of tumorigenicity 7 protein-like protein partial	custom
90508	2334	Peroxiredoxin-6 partial	custom
NA	2337	hypothetical protein X975 26413 partial	custom
NA	2341	Solute carrier family 35 member F5 partial	custom
NA	2343	putative deoxyhypusine synthase partial	custom
NA	2346	Intraflagellar transport protein 80-like protein partial	custom

Supplemental Table 3 – continued from previous page

Arthropod Core ID	Spider Core ID	Putative Ortholog Description	Target Database
89727	2347	Transcription initiation factor IIB partial	custom
90621	2350	Oxysterol-binding protein-related protein 1 partial	custom
NA	2354	Cytoskeleton-associated protein 5 partial	custom
NA	2360	hypothetical protein X975 00287 partial	custom
NA	2362	Neuroblastoma-amplified sequence partial	custom
NA	2364	hypothetical protein X975 00641 partial	custom
NA	2366	Ras-related protein ced-10 partial	custom
NA	2367	Lipoamide acyltransferase component of branched-chain alpha-keto acid dehydrogenase complex mitochondrial partial	custom
NA	2369	SCO-spondin partial	custom
89708	2370	39S ribosomal protein L48 mitochondrial partial	custom
NA	2371	Lysosomal protein NCU-G1 partial	custom
NA	2373	Rab5 GDP/GTP exchange factor partial	custom
NA	2374	Serine/threonine-protein kinase PAK 1 partial	custom
NA	2376	Serine/threonine-protein kinase N2 partial	custom
NA	2378	hypothetical protein X975 25159 partial	custom
NA	2386	Cytochrome b5 reductase 4 partial	custom
NA	2389	Growth hormone-regulated TBC protein 1-A partial	custom
NA	2396	SAM domain and HD domain-containing protein 1 partial	custom

Supplemental Table 3 – continued from previous page

Arthropod Core ID	Spider Core ID	Putative Ortholog Description	Target Database
NA	2402	hypothetical protein X975 04812 partial	custom
90637	2403	3-phosphoinositide-dependent protein kinase 1 partial	custom
NA	2410	Ubiquitin-protein ligase E3C partial	custom
NA	2418	Signal-induced proliferation-associated 1-like protein 2 partial	custom
NA	2421	Geranylgeranyl pyrophosphate synthase partial	custom
NA	2427	hypothetical protein X975 18306 partial	custom
NA	2441	G kinase-anchoring protein 1 partial	custom
NA	2442	Tyrosine-protein phosphatase non-receptor type 4 partial	custom
NA	2443	hypothetical protein X975 07353 partial	custom
NA	2444	Sorting and assembly machinery component 50-like protein partial	custom
NA	2445	Zinc phosphodiesterase ELAC protein 2 partial	custom
NA	2446	Glycylpeptide N-tetradecanoyltransferase 1 partial	custom
NA	2447	hypothetical protein X975 14365 partial	custom
NA	2448	NADP-dependent malic enzyme partial	custom
NA	2449	ADP-sugar pyrophosphatase partial	custom
89970	2450	Phosphoserine aminotransferase partial	custom
NA	2451	28S ribosomal protein S35 mitochondrial partial	custom
NA	2452	Autism susceptibility 2 protein partial	custom

Supplemental Table 3 – continued from previous page

Arthropod Core ID	Spider Core ID	Putative Ortholog Description	Target Database
NA	2453	Protein FAM63B partial	custom
NA	2454	hypothetical protein X975 07416 partial	custom
NA	2455	MKI67 FHA domain-interacting nucleolar phosphoprotein-like protein partial	custom
NA	2456	Glucose-6-phosphate isomerase partial	custom
NA	2458	Neuroendocrine convertase 1 partial	custom
90652	2460	PREDICTED: PRKCA-binding protein-like	nr
NA	2461	hypothetical protein X975 01092 partial	custom
NA	2462	hypothetical protein X975 17265 partial	custom
NA	2463	TAR DNA-binding protein 43 partial	custom
NA	2465	Isovaleryl-CoA dehydrogenase mitochondrial partial	custom
90203	2466	DnaJ-like protein subfamily B member 9 partial	custom
NA	2468	ZZ-type zinc finger-containing protein 3 partial	custom
NA	2469	Leucine-rich repeat-containing protein 49 partial	custom
NA	2470	hypothetical protein X975 02761 partial	custom
NA	2471	Spliceosome RNA helicase DDX39B partial	custom
NA	2472	hypothetical protein X975 08449 partial	custom
NA	2473	NudC domain-containing protein 1 partial	custom
NA	2474	PRELI domain-containing protein 1 mitochondrial partial	custom

Supplemental Table 3 – continued from previous page

Arthropod Core ID	Spider Core ID	Putative Ortholog Description	Target Database
NA	2475	PREDICTED: centrosomal protein of 19 kDa-like	nr
NA	2476	Lambda-crystallin-like protein partial	custom
NA	2477	Elongator complex protein 2 partial	custom
NA	2479	Serum amyloid A-2 protein partial	custom
NA	2480	Ribonuclease 3 partial	custom
90442	2482	Guanine nucleotide-binding protein-like 3-like protein partial	custom
NA	2483	Mediator of RNA polymerase II transcription subunit 11 partial	custom
NA	2484	Transducin beta-like protein 2 partial	custom
NA	2485	putative E3 ubiquitin-protein ligase UBR7 partial	custom
NA	2487	Mitochondrial import receptor subunit TOM22-like protein partial	custom
90211	2488	60S acidic ribosomal protein P0 partial	custom
NA	2489	Peroxisomal acyl-coenzyme A oxidase 3 partial	custom
NA	2490	26S protease regulatory subunit 6B partial	custom
NA	2491	Metaxin-1 partial	custom
NA	2492	E3 SUMO-protein ligase RanBP2 partial	custom
NA	2493	Myeloid leukemia factor 1 partial	custom
90438	2494	Synaptosomal-associated protein 25 partial	custom
90522	2495	Profilin-5 partial	custom
89882	2498	Transcription initiation factor TFIID subunit 5 partial	custom

Supplemental Table 3 – continued from previous page

Arthropod Core ID	Spider Core ID	Putative Ortholog Description	Target Database
NA	2501	Glycerol-3-phosphate dehydrogenase 1-like protein partial	custom
89688	2503	Peptide methionine sulfoxide reductase partial	custom
NA	2504	Gamma-aminobutyric acid receptor-associated protein partial	custom
89822	2505	Serine/arginine-rich splicing factor 4 partial	custom
90301	2507	Hydroxysteroid dehydrogenase-like protein 2 partial	custom
NA	2508	Translation initiation factor IF-2 mitochondrial partial	custom
NA	2509	hypothetical protein X975 05876 partial	custom
NA	2511	Conserved oligomeric Golgi complex subunit 7 partial	custom
NA	2512	Protein phosphatase 1B partial	custom
90605	2513	Nuclear cap-binding protein subunit 2 partial	custom
NA	2515	Dixin partial	custom
90618	2516	Proliferation-associated protein 2G4 partial	custom
NA	2518	4-hydroxybutyrate coenzyme A transferase partial	custom
NA	2519	Medium-chain specific acyl-CoA dehydrogenase mitochondrial partial	custom
89990	2522	Rab GDP dissociation inhibitor beta partial	custom
NA	2524	Coiled-coil domain-containing protein 43 partial	custom
90107	2526	Calreticulin partial	custom

Supplemental Table 3 – continued from previous page

Arthropod Core ID	Spider Core ID	Putative Ortholog Description	Target Database
NA	2528	hypothetical protein X975 16227 partial	custom
NA	2529	Protein lifeguard 2 partial	custom
NA	2530	Eukaryotic translation initiation factor 4B partial	custom
89707	2532	Acetyl-CoA acetyltransferase A mitochondrial partial	custom
90099	2533	Dolichyl-diphosphooligosaccharide–protein glycosyltransferase subunit 1 partial	custom
NA	2534	Squamous cell carcinoma antigen recognized by T-cells 3 partial	custom
90343	2535	putative isocitrate dehydrogenase [NAD] subunit beta mitochondrial partial	custom
NA	2537	Vascular endothelial growth factor A partial	custom
NA	2538	Krueppel-like factor 15 partial	custom
NA	2539	NA	nr
90426	2540	Polyadenylate-binding protein 1 partial	custom
NA	2541	Superoxide dismutase [Cu-Zn] partial	custom
NA	2545	Eukaryotic translation initiation factor 3 subunit L partial	custom
NA	2547	DNA-directed RNA polymerase III subunit RPC5 partial	custom
NA	2548	calcium-independent phospholipase A2 partial	custom
NA	2549	T-complex protein 1 subunit zeta partial	custom
NA	2550	Histone acetyltransferase KAT8 partial	custom

Arthropod Core ID	Spider Core ID	Putative Ortholog Description	Target Database
NA	2551	NA	nr
89657	2556	UDP-glucose 6-dehydrogenase partial	custom
NA	2557	Transmembrane protein 68 partial	custom
NA	2558	Vacuolar fusion protein MON1-like protein partial	custom
NA	2559	Heparan-alpha-glucosaminide N-acetyltransferase partial	custom
NA	2560	Nucleoporin Nup43 partial	custom
90542	2561	Eukaryotic translation initiation factor 2D partial	custom
90272	2562	Beta-glucuronidase partial	custom
NA	2563	CDK5 regulatory subunit-associated protein 1 partial	custom
NA	2565	Dehydrogenase/reductase SDR family protein 7-like protein partial	custom
NA	2568	Coiled-coil domain-containing protein 50 partial	custom
NA	2569	Ribokinase partial	custom
NA	2570	Geranylgeranyl transferase type-2 subunit alpha partial	custom
NA	2571	Dimethyladenosine transferase 1 mitochondrial partial	custom
NA	2573	PREDICTED: COMM domain-containing protein 5-like	nr
NA	2575	Peroxisomal carnitine O-octanoyltransferase partial	custom
NA	2576	Programmed cell death protein 6 partial	custom

Supplemental Table 3 – continued from previous page

Arthropod Core ID	Spider Core ID	Putative Ortholog Description	Target Database
NA	2578	Calcium and integrin-binding protein 1 partial	custom
NA	2580	Rhombotin-1 partial	custom
NA	2581	Protein phosphatase 1K mitochondrial partial	custom
NA	2582	Protein GPR107 partial	custom
90479	2583	RNA-binding protein pno1 partial	custom
NA	2585	hypothetical protein X975 03921 partial	custom
NA	2586	Ras-related protein Rab-7a partial	custom
NA	2588	NGFI-A-binding protein-like protein partial	custom
NA	2590	Transcriptional enhancer factor TEF-1 partial	custom
NA	2591	Leukotriene A-4 hydrolase partial	custom
NA	2593	Nicolin-1 partial	custom
NA	2594	Charged multivesicular body protein 1b partial	custom
NA	2597	hypothetical protein X975 02228 partial	custom
NA	2598	PKHD domain-containing transmembrane protein partial	custom
90315	2599	NEDD8-activating enzyme E1 regulatory subunit partial	custom
NA	2601	Hydroxyacid-oxoacid transhydrogenase mitochondrial partial	custom
NA	2602	Gamma-aminobutyric acid type B receptor subunit 1 partial	custom
NA	2605	Neural cell adhesion molecule 1 partial	custom

Supplemental Table 3 – continued from previous page

Arthropod Core ID	Spider Core ID	Putative Ortholog Description	Target Database
NA	2607	Anaphase-promoting complex subunit 5 partial	custom
NA	2611	Apoptosis-inducing factor 1 mitochondrial partial	custom
NA	2615	Methionine–tRNA ligase mitochondrial partial	custom
NA	2616	ATP-dependent RNA helicase DDX24 partial	custom
NA	2618	putative serine hydrolase partial	custom
NA	2619	Connectin partial	custom
90639	2620	3-hydroxyacyl-CoA dehydrogenase type-2 partial	custom
NA	2621	Centrosomal protein partial	custom
90079	2624	tRNA (cytosine(34)-C(5))-methyltransferase partial	custom
NA	2626	Neuronal migration protein doublecortin partial	custom
NA	2627	Mitochondrial ornithine transporter 1 partial	custom
90223	2628	E3 UFM1-protein ligase 1 partial	custom
NA	2629	Golgin subfamily A member 5 partial	custom
NA	2631	hypothetical protein X975 04394 partial	custom
NA	2632	Mitochondrial ribonuclease P protein 3 partial	custom
NA	2633	hypothetical protein X975 10778 partial	custom
89774	2634	Signal recognition particle protein partial	custom
NA	2635	hypothetical protein X975 17473 partial	custom

Supplemental Table 3 – continued from previous page

Arthropod Core ID	Spider Core ID	Putative Ortholog Description	Target Database
90008	2637	Beta-ureidopropionase partial	custom
NA	2638	TIP41-like protein partial	custom
NA	2644	hypothetical protein X975 14917 partial	custom
90571	2646	Exocyst complex component 8 partial	custom
NA	2649	Hydroxyacyl-coenzyme A dehydrogenase mitochondrial partial	custom
NA	2652	WD repeat-containing protein 91 partial	custom
NA	2653	putative palmitoyltransferase ZDHHC14 partial	custom
89816	2654	ATP-dependent RNA helicase DDX51 partial	custom
NA	2655	hypothetical protein X975 07487 partial	custom
NA	2657	Trafficking protein particle complex subunit 8 partial	custom
NA	2660	DCN1-like protein 1 partial	custom
NA	2661	Endoribonuclease Dcr-1 partial	custom
NA	2662	Dynactin subunit 4 partial	custom
89917	2663	Phosphatidylinositol-45-bisphosphate 3-kinase catalytic subunit beta isoform partial	custom
NA	2665	Phosphatidylinositol-345-trisphosphate 3-phosphatase TPTE2 partial	custom
NA	2666	hypothetical protein X975 22847 partial	custom
NA	2668	4-aminobutyrate aminotransferase mitochondrial partial	custom

Supplemental Table 3 – continued from previous page

Arthropod Core ID	Spider Core ID	Putative Ortholog Description	Target Database
NA	2675	Cleavage stimulation factor subunit 3 partial	custom
NA	2678	FUN14 domain-containing protein 1 partial	custom
NA	2683	Replication factor C subunit 1 partial	custom
NA	2690	Phosphoribosylformylglycinamidine synthase partial	custom
NA	2694	Junctophilin-1 partial	custom
NA	2695	Zinc finger protein 592 partial	custom
NA	2696	putative RNA-binding protein 19 partial	custom
NA	2697	4-hydroxybenzoate polyprenyltransferase mitochondrial partial	custom
NA	2698	Sentrin-specific protease 1 partial	custom
NA	2700	putative ATP-dependent RNA helicase DHX37 partial	custom
90127	2701	hypothetical protein X975 17968 partial	custom
90472	2702	Heterogeneous nuclear ribonucleoprotein D-like protein partial	custom
NA	2704	putative E3 ubiquitin-protein ligase MGRN1 partial	custom
NA	2705	GTP-binding protein Rheb-like protein partial	custom
NA	2706	Protein DEK partial	custom
NA	2707	Cyclin-dependent kinase 8 partial	custom
NA	2708	Flavin reductase (NADPH) partial	custom
NA	2709	Glutathione S-transferase Mu 1 partial	custom
NA	2710	hypothetical protein X975 10107 partial	custom

Supplemental Table 3 – continued from previous page

Arthropod Core ID	Spider Core ID	Putative Ortholog Description	Target Database
90481	2711	NADH dehydrogenase [ubiquinone] flavoprotein 1 mitochondrial partial	custom
89765	2712	Protein OS-9 partial	custom
NA	2713	Eukaryotic translation initiation factor 2 subunit 1 partial	custom
NA	2715	HIG1 domain family member 2A partial	custom
90145	2717	40S ribosomal protein S30 partial	custom
NA	2718	Mitochondrial-processing peptidase subunit beta partial	custom
NA	2720	Pseudouridine-5'-monophosphatase partial	custom
90564	2721	ATP-binding cassette sub-family F member 1 partial	custom
90141	2722	C-terminal-binding protein partial	custom
90638	2723	Charged multivesicular body protein 4b partial	custom
NA	2724	Glucose-6-phosphate translocase partial	custom
NA	2725	Basigin partial	custom
NA	2726	Ankyrin repeat domain-containing protein 13C partial	custom
NA	2729	Muscle-specific protein 20 partial	custom
NA	2730	Myotubularin-related protein 2 partial	custom
NA	2731	Alcohol dehydrogenase class-3 chain L partial	custom
90601	2733	Conserved oligomeric Golgi complex subunit 4 partial	custom
NA	2735	COP9 signalosome complex subunit 3 partial	custom
NA	2737	Nucleolar transcription factor 1-B partial	custom

Supplemental Table 3 – continued from previous page

Arthropod Core ID	Spider Core ID	Putative Ortholog Description	Target Database
NA	2738	Leucine-rich repeat-containing protein 47 partial	custom
NA	2739	Multidrug resistance-associated protein 1 partial	custom
NA	2740	DNA-directed RNA polymerase II subunit RPB4 partial	custom
90022	2741	Lipoyl synthase mitochondrial partial	custom
NA	2743	28S ribosomal protein S22 mitochondrial partial	custom
89651	2744	Beta-14-mannosyltransferase egh partial	custom
NA	2746	Protein O-mannosyl-transferase 2 partial	custom
89802	2748	Pre-mRNA-splicing factor syf2 partial	custom
NA	2751	UBX domain-containing protein 1-A partial	custom
NA	2752	CKLF-like MARVEL transmembrane domain-containing protein 4 partial	custom
NA	2753	Tubulin polyglutamylase TTLL4 partial	custom
NA	2755	Cytochrome c oxidase assembly protein COX11 mitochondrial partial	custom
NA	2756	Protein MEF2BNB-like protein partial	custom
NA	2757	Peptidyl-prolyl cis-trans isomerase FKBP2 partial	custom
NA	2758	Carboxypeptidase A2 partial	custom
NA	2759	hypothetical protein X975 13772 partial	custom
NA	2761	hypothetical protein X975 05727 partial	custom
NA	2762	Death-associated protein 1 partial	custom

Supplemental Table 3 – continued from previous page

Arthropod Core ID	Spider Core ID	Putative Ortholog Description	Target Database
NA	2764	hypothetical protein X975 22094 partial	custom
NA	2766	Developmentally-regulated GTP-binding protein 2 partial	custom
NA	2768	hypothetical protein X975 03910 partial	custom
90178	2769	Glyoxalase domain-containing protein 4 partial	custom
NA	2770	Methyltransferase-like protein 14 partial	custom
90054	2771	Proliferating cell nuclear antigen partial	custom
NA	2773	Protein bunched class 2/F isoform partial	custom
NA	2774	Voltage-dependent anion-selective channel protein 2 partial	custom
NA	2775	Alcohol dehydrogenase class-3 partial	custom
NA	2776	[3-methyl-2-oxobutanoate dehydrogenase [lipoamide]] kinase mitochondrial partial	custom
89696	2777	Mitochondrial-processing peptidase subunit beta partial	custom
NA	2778	NA	nr
NA	2779	Hydroxysteroid dehydrogenase-like protein 1 partial	custom
NA	2780	Ubiquitin-like protein ATG12 partial	custom
89717	2781	SUMO-activating enzyme subunit 1 partial	custom
NA	2783	Mitochondrial import inner membrane translocase subunit TIM44 partial	custom
NA	2784	hypothetical protein X975 19443 partial	custom

Supplemental Table 3 – continued from previous page

Arthropod Core ID	Spider Core ID	Putative Ortholog Description	Target Database
NA	2785	Protein deadpan partial	custom
NA	2786	Calponin-3 partial	custom
NA	2787	Protein VPRBP partial	custom
NA	2788	Ero1-like protein partial	custom
89956	2792	T-complex protein 1 subunit gamma partial	custom
NA	2793	Ubiquitin-conjugating enzyme E2 R2 partial	custom
NA	2794	Calcyclin-binding protein partial	custom
NA	2795	RWD domain-containing protein 4 partial	custom
NA	2796	Zinc transporter ZIP1 partial	custom
NA	2797	Hydroxyacid oxidase 1 partial	custom
NA	2798	Serine-threonine kinase receptor-associated protein partial	custom
NA	2800	hypothetical protein X975 19628 partial	custom
NA	2801	Mesencephalic astrocyte-derived neurotrophic factor-like protein partial	custom
NA	2802	Cytosolic non-specific dipeptidase partial	custom
NA	2803	hypothetical protein X975 25821 partial	custom
90646	2804	Cystathionine gamma-lyase partial	custom
NA	2806	Peptidyl-prolyl cis-trans isomerase B partial	custom
NA	2808	Selenoprotein S partial	custom

Supplemental Table 3 – continued from previous page

Arthropod Core ID	Spider Core ID	Putative Ortholog Description	Target Database
NA	2810	NADH dehydrogenase [ubiquinone] 1 alpha subcomplex subunit 10 mitochondrial partial	custom
NA	2811	Glutaryl-CoA dehydrogenase mitochondrial partial	custom
NA	2812	hypothetical protein X975 12951 partial	custom
90506	2813	Translocation protein SEC62 partial	custom
NA	2814	Alpha-centractin partial	custom
NA	2815	Dipeptidyl peptidase 1 partial	custom
NA	2816	DnaJ-like protein subfamily A member 2 partial	custom
NA	2817	Y+L amino acid transporter 2 partial	custom
NA	2818	2-oxoisovalerate dehydrogenase subunit alpha mitochondrial partial	custom
90111	2819	26S protease regulatory subunit 6A partial	custom
NA	2820	Splicing factor 3B subunit 4 partial	custom
NA	2821	Secernin-2 partial	custom
NA	2822	hypothetical protein X975 00118 partial	custom
NA	2823	Bleomycin hydrolase partial	custom
NA	2824	T-complex protein 1 subunit alpha partial	custom
NA	2825	Xaa-Pro dipeptidase partial	custom
NA	2828	Tribbles-like protein partial	custom
NA	2829	Carboxypeptidase E partial	custom
NA	2830	Short-chain specific acyl-CoA dehydrogenase mitochondrial partial	custom

Supplemental Table 3 – continued from previous page

Arthropod Core ID	Spider Core ID	Putative Ortholog Description	Target Database
NA	2831	Leucine-rich repeat-containing protein 15 partial	custom
NA	2833	hypothetical protein X975 02652 partial	custom
90182	2837	Periodic tryptophan protein 1-like protein partial	custom
NA	2838	hypothetical protein X975 00948 partial	custom
NA	2840	hypothetical protein X975 15601 partial	custom
NA	2841	hypothetical protein X975 25326 partial	custom
NA	2842	Ubiquitin carboxyl-terminal hydrolase 16 partial	custom
NA	2843	Serine/threonine-protein kinase PLK1 partial	custom
NA	2844	Polycomb protein Suz12 partial	custom
NA	2847	hypothetical protein X975 16220 partial	custom
NA	2848	Ubiquitin-conjugating enzyme E2 W partial	custom
NA	2850	Down syndrome critical region protein 3-like protein partial	custom
90627	2851	Short coiled-coil protein partial	custom
NA	2852	Transmembrane channel-like protein 7 partial	custom
NA	2853	Zinc finger CCHC domain-containing protein 4 partial	custom
NA	2855	RNA-binding protein NOB1 partial	custom
90372	2857	Ubiquitin carboxyl-terminal hydrolase BAP1 partial	custom
NA	2858	Acetyl-coenzyme A transporter 1 partial	custom
NA	2859	Pyroglutamyl-peptidase 1 partial	custom

Supplemental Table 3 – continued from previous page

Arthropod Core ID	Spider Core ID	Putative Ortholog Description	Target Database
NA	2861	Sec1 family domain-containing protein 1 partial	custom
90514	2862	NADH dehydrogenase [ubiquinone] iron-sulfur protein 4 mitochondrial partial	custom
NA	2863	Arginyl-tRNA–protein transferase 1 partial	custom
NA	2864	Stress-induced-phosphoprotein 1 partial	custom
NA	2865	OCIA domain-containing protein 1 partial	custom
NA	2866	Carbohydrate sulfotransferase 6 partial	custom
NA	2868	Sestrin-3 partial	custom
NA	2869	26S proteasome non-ATPase regulatory subunit 3 partial	custom
NA	2870	Bis(5'-nucleosyl)-tetraphosphatase symmetrical partial	custom
90197	2871	Golgi phosphoprotein 3 partial	custom
NA	2876	Acyl-CoA dehydrogenase family member 9 mitochondrial partial	custom
90104	2878	DNA-directed RNA polymerase III subunit RPC3 partial	custom
NA	2879	Negative elongation factor B partial	custom
NA	2880	putative tRNA threonylcarbamoyladenosine biosynthesis protein osgep partial	custom
NA	2881	ADP-dependent glucokinase partial	custom
NA	2882	Muskelin partial	custom
NA	2885	Guanine nucleotide-binding protein G(o) subunit alpha partial	custom
NA	2891	Ribulose-phosphate 3-epimerase partial	custom

Supplemental Table 3 – continued from previous page

Arthropod Core ID	Spider Core ID	Putative Ortholog Description	Target Database
NA	2894	E3 ubiquitin-protein ligase Hakai partial	custom
NA	2895	Protein zyg-11-like protein partial	custom
NA	2897	Nicotinamide mononucleotide adenylyltransferase 1 partial	custom
NA	2898	Diphthamide biosynthesis protein 2 partial	custom
NA	2899	hypothetical protein X975 23643 partial	custom
NA	2900	DNA polymerase alpha subunit B partial	custom
NA	2901	Sarcosine dehydrogenase mitochondrial partial	custom
NA	2902	N-alpha-acetyltransferase 15 NatA auxiliary subunit partial	custom
NA	2903	Transcription factor 20 partial	custom
NA	2904	Conserved oligomeric Golgi complex subunit 2 partial	custom
NA	2906	Xylulose kinase partial	custom
NA	2908	hypothetical protein X975 01820 partial	custom
NA	2909	Protein salvador-like protein partial	custom
NA	2910	Protein ced-11 partial	custom
NA	2914	hypothetical protein X975 20345 partial	custom
NA	2915	Acylamino-acid-releasing enzyme partial	custom
NA	2916	UDP-glucuronic acid decarboxylase 1 partial	custom
NA	2919	hypothetical protein X975 04267 partial	custom
NA	2920	ATP-dependent RNA helicase DDX54 partial	custom

Supplemental Table 3 – continued from previous page

Arthropod Core ID	Spider Core ID	Putative Ortholog Description	Target Database
NA	2922	Glutamate-gated chloride channel partial	custom
NA	2923	Magnesium transporter NIPA2 partial	custom
NA	2925	DAZ-associated protein 2 partial	custom
NA	2926	Mitochondrial ribonuclease P protein 1-like protein partial	custom
NA	2927	Copper chaperone for superoxide dismutase partial	custom
NA	2928	RING finger and transmembrane domain-containing protein 2 partial	custom
90198	2929	WD repeat-containing protein 36 partial	custom
NA	2930	Low density lipoprotein receptor adapter protein 1-B partial	custom
NA	2933	Remodeling and spacing factor 1 partial	custom
NA	2934	Lys-63-specific deubiquitinase partial	custom
NA	2936	hypothetical protein X975 05388 partial	custom
NA	2937	Integrator complex subunit 11 partial	custom
NA	2938	WD repeat-containing protein 61 partial	custom
NA	2939	Ubiquitin-associated protein 1 partial	custom
NA	2940	PREDICTED: ubiquitin-conjugating enzyme E2-24 kDa-like	nr
NA	2941	PDZ domain-containing protein GIPC1 partial	custom
NA	2942	Coiled-coil domain-containing protein 93 partial	custom

Supplemental Table 3 – continued from previous page

Arthropod Core ID	Spider Core ID	Putative Ortholog Description	Target Database
NA	2943	putative sodium-coupled neutral amino acid transporter 7 partial	custom
NA	2944	Tetratricopeptide repeat protein 5 partial	custom
NA	2945	COP9 signalosome complex subunit 7a partial	custom
NA	2946	Vacuolar fusion protein CCZ1-like protein partial	custom
NA	2947	hypothetical protein X975 17695 partial	custom
NA	2948	Platelet-activating factor acetylhydrolase partial	custom
NA	2949	Epsin-2 partial	custom
NA	2951	BRO1 domain-containing protein BROX partial	custom
NA	2953	Exportin-T partial	custom
NA	2955	Ubiquitin carboxyl-terminal hydrolase 20 partial	custom
NA	2956	Vacuolar protein sorting-associated protein 33A partial	custom
NA	2957	CDK5 regulatory subunit-associated protein 1-like 1 partial	custom
NA	2959	Coiled-coil domain-containing protein 12 partial	custom
NA	2961	F-box/LRR-repeat protein 4 partial	custom
90169	2963	Cytosolic Fe-S cluster assembly factor NARFL partial	custom
89841	2968	X-linked retinitis pigmentosa GTPase regulator-like protein partial	custom
NA	2970	hypothetical protein X975 02246 partial	custom
NA	2972	Dysbindin partial	custom

Supplemental Table 3 – continued from previous page

Arthropod Core ID	Spider Core ID	Putative Ortholog Description	Target Database
NA	2973	Zinc finger MYM-type protein 4 partial	custom
NA	2974	hypothetical protein X975 11700 partial	custom
NA	2975	Nuclear migration protein nudC partial	custom
NA	2977	Ras-related protein Rab-1D partial	custom
NA	2978	Protein SAAL1 partial	custom
NA	2980	Ubiquinone biosynthesis monooxygenase COQ6 partial	custom
NA	2981	Beta-catenin-like protein 1 partial	custom
90166	2984	Beta-mannosidase partial	custom
NA	2986	Peroxisomal membrane protein PMP34 partial	custom
NA	2988	Frizzled-4 partial	custom
90038	2989	TBC1 domain family member 22B partial	custom
90449	2993	hypothetical protein X975 08773 partial	custom
NA	2994	hypothetical protein X975 19804 partial	custom
NA	2996	Decaprenyl-diphosphate synthase subunit 1 partial	custom
NA	3000	Negative elongation factor C/D partial	custom
89900	3007	Nuclear pore complex protein Nup93 partial	custom
NA	3013	hypothetical protein X975 21297 partial	custom
NA	3026	Alpha-N-acetylglucosaminidase partial	custom
NA	3028	Ornithine decarboxylase partial	custom
NA	3035	hypothetical protein X975 24787 partial	custom
NA	3037	Methyltransferase-like protein 17 mitochondrial partial	custom

Supplemental Table 3 – continued from previous page

Arthropod Core ID	Spider Core ID	Putative Ortholog Description	Target Database
NA	3038	Single-stranded DNA-binding protein mitochondrial partial	custom
NA	3039	Cleavage and polyadenylation specificity factor subunit 3 partial	custom
NA	3041	WD repeat-containing protein 74 partial	custom
90385	3044	39S ribosomal protein L40 mitochondrial partial	custom
NA	3045	28S ribosomal protein S15 mitochondrial partial	custom
NA	3047	Transmembrane protein 183 partial	custom
NA	3049	Charged multivesicular body protein 2a partial	custom
90090	3052	Inositol-3-phosphate synthase 1-A partial	custom
NA	3053	Poly(ADP-ribose) glycohydrolase partial	custom
NA	3054	Nesprin-2 partial	custom
NA	3055	Methionine–tRNA ligase cytoplasmic partial	custom
90528	3056	Protein arginine N-methyltransferase 5 partial	custom
90644	3058	Paired amphipathic helix protein Sin3b partial	custom
NA	3059	UPF0661 TPR repeat-containing protein partial	custom
90538	3060	Soluble calcium-activated nucleotidase 1 partial	custom
NA	3061	Cytochrome b-c1 complex subunit 2 mitochondrial partial	custom
NA	3062	heat- and acid-stable phosphoprotein partial	custom

Supplemental Table 3 – continued from previous page

Arthropod Core ID	Spider Core ID	Putative Ortholog Description	Target Database
NA	3063	Vesicle-associated membrane protein/synaptobrevin-binding protein partial	custom
NA	3064	NADH-cytochrome b5 reductase 2 partial	custom
NA	3066	Peptidase M20 domain-containing protein 2 partial	custom
NA	3067	Ankyrin repeat domain-containing protein 13B partial	custom
NA	3068	Ras-related protein Ral-a partial	custom
NA	3069	Phenylalanine–tRNA ligase beta subunit partial	custom
NA	3071	Peptidyl-tRNA hydrolase 2 mitochondrial partial	custom
90574	3072	Cleavage stimulation factor subunit 2 partial	custom
89677	3073	Actin-like protein 6A partial	custom
NA	3075	Transcription initiation factor TFIID subunit 10 partial	custom
90515	3076	60S ribosomal protein L35 partial	custom
NA	3077	Guanine nucleotide-binding protein subunit beta-2-like 1 partial	custom
NA	3078	Carbonic anhydrase 1 partial	custom
NA	3079	U5 small nuclear ribonucleoprotein partial	custom
NA	3080	Thioredoxin-related transmembrane protein 1 partial	custom
NA	3083	Importin subunit alpha-7 partial	custom
89913	3084	Membrane-associated progesterone receptor component 2 partial	custom

Supplemental Table 3 – continued from previous page

Arthropod Core ID	Spider Core ID	Putative Ortholog Description	Target Database
NA	3085	DNA-directed RNA polymerase III subunit RPC7 partial	custom
NA	3086	Tropomyosin partial	custom
NA	3087	WASH complex subunit FAM21 partial	custom
NA	3090	hypothetical protein X975 08459 partial	custom
90299	3092	Actin-related protein 6 partial	custom
90645	3093	Multiple coagulation factor deficiency protein 2-like protein partial	custom
NA	3094	Pterin-4-alpha-carbinolamine dehydratase partial	custom
NA	3095	Prefoldin subunit 3 partial	custom
NA	3096	LDLR chaperone boca partial	custom
NA	3097	Alpha-13-mannosyl-glycoprotein 2-beta-N-acetylglucosaminyltransferase partial	custom
NA	3098	Translocon-associated protein subunit delta partial	custom
NA	3099	Protein TMED8 partial	custom
NA	3100	Ankyrin repeat family A protein 2 partial	custom
NA	3102	WD40 repeat-containing protein SMU1 partial	custom
NA	3103	Leukotriene A-4 hydrolase partial	custom
89786	3104	Adenylate kinase 2 mitochondrial partial	custom
NA	3106	hypothetical protein X975 03439 partial	custom
NA	3107	Endoribonuclease Dcr-1 partial	custom
NA	3108	Pleiotropic regulator 1 partial	custom

Supplemental Table 3 – continued from previous page

Arthropod Core ID	Spider Core ID	Putative Ortholog Description	Target Database
NA	3110	Actin-related protein 2/3 complex subunit 3 partial	custom
NA	3111	Triosephosphate isomerase B partial	custom
NA	3112	Carbonic anhydrase 7 partial	custom
NA	3114	Alpha-aspartyl dipeptidase partial	custom
89695	3115	Polyadenylate-binding protein 2 partial	custom
90321	3117	NADH dehydrogenase [ubiquinone] iron-sulfur protein 8 mitochondrial partial	custom
NA	3118	Charged multivesicular body protein 3 partial	custom
NA	3120	Eukaryotic translation initiation factor 4H partial	custom
NA	3121	Alpha-endosulfine partial	custom
NA	3122	17-beta-hydroxysteroid dehydrogenase type 6 partial	custom
NA	3123	ATP synthase subunit b mitochondrial partial	custom
NA	3124	Protein arginine N-methyltransferase 7 partial	custom
NA	3125	Translocon-associated protein subunit alpha partial	custom
NA	3126	General transcription factor IIF subunit 1 partial	custom
90589	3127	Cytochrome c-type heme lyase partial	custom
90015	3128	NADH dehydrogenase [ubiquinone] iron-sulfur protein 2 mitochondrial partial	custom
NA	3129	Secreted frizzled-related protein 5 partial	custom

Supplemental Table 3 – continued from previous page

Arthropod Core ID	Spider Core ID	Putative Ortholog Description	Target Database
NA	3130	Protein YIPF1 partial	custom
NA	3133	beta-galactoside-binding lectin partial	custom
NA	3135	UDP-glucose 4-epimerase partial	custom
NA	3136	Protein Skeletor isoforms B/C partial	custom
89880	3137	Tumor suppressor candidate 3 partial	custom
NA	3140	Translin partial	custom
NA	3141	Aspartate aminotransferase cytoplasmic partial	custom
NA	3142	DNA replication licensing factor mcm2 partial	custom
90273	3144	Zygotic DNA replication licensing factor mcm3 partial	custom
NA	3145	Inhibitor of Bruton tyrosine kinase partial	custom
89908	3146	Phosphoglycerate kinase 1 partial	custom
NA	3147	hypothetical protein X975 19489 partial	custom
NA	3148	Transferrin partial	custom
NA	3149	putative 39S ribosomal protein L24 mitochondrial partial	custom
90226	3150	Interleukin enhancer-binding factor 2-like protein partial	custom
NA	3151	Retinol dehydrogenase 12 partial	custom
89792	3152	FACT complex subunit SSRP1 partial	custom
89890	3155	hypothetical protein X975 26470 partial	custom
NA	3157	Ras-related protein Rab-5C partial	custom
NA	3158	Flagellar radial spoke protein 3 partial	custom
NA	3159	Neuferricin partial	custom
NA	3160	COMM domain-containing protein 8 partial	custom

Supplemental Table 3 – continued from previous page

Arthropod Core ID	Spider Core ID	Putative Ortholog Description	Target Database
NA	3161	Myeloid differentiation primary response protein MyD88 partial	custom
NA	3163	hypothetical protein X975 04393 partial	custom
89976	3164	WD repeat-containing protein 82 partial	custom
NA	3166	Protein-L-isoaspartate O-methyltransferase domain-containing protein 2 partial	custom
NA	3167	Abhydrolase domain-containing protein 16A partial	custom
NA	3168	Krueppel-like factor 11 partial	custom
NA	3171	Methylmalonyl-CoA epimerase mitochondrial partial	custom
NA	3172	hypothetical protein X975 19022 partial	custom
NA	3173	ATP-binding domain-containing protein 4 partial	custom
NA	3174	hypothetical protein X975 10848 partial	custom
90557	3176	Angiogenic factor with G patch and FHA domains 1 partial	custom
NA	3177	Protein BCCIP-like protein partial	custom
NA	3178	SPARC-related modular calcium-binding protein 1 partial	custom
NA	3181	Coiled-coil domain-containing protein 132 partial	custom
NA	3182	Interferon-related developmental regulator 1 partial	custom
NA	3183	Mortality factor 4-like protein 1 partial	custom
90569	3184	Mitotic checkpoint protein BUB3 partial	custom

Supplemental Table 3 – continued from previous page

Arthropod Core ID	Spider Core ID	Putative Ortholog Description	Target Database
NA	3185	hypothetical protein X975 21997 partial	custom
NA	3186	NA	nr
NA	3187	hypothetical protein X975 10342 partial	custom
NA	3188	Tubulin-specific chaperone C partial	custom
NA	3190	Tyrosine–tRNA ligase cytoplasmic partial	custom
NA	3191	Microtubule-associated proteins 1A/1B light chain 3C partial	custom
NA	3192	Nuclear pore glycoprotein p62 partial	custom
NA	3196	PREDICTED: uncharacterized protein C14orf119 homolog	nr
NA	3197	Kazal-type serine protease inhibitor domain-containing protein 1 partial	custom
NA	3198	General transcription factor IIE subunit 1 partial	custom
NA	3201	GPN-loop GTPase 1 partial	custom
NA	3202	EF-hand calcium-binding domain-containing protein 7 partial	custom
NA	3204	hypothetical protein X975 20481 partial	custom
NA	3205	putative glucose-6-phosphate 1-epimerase partial	custom
NA	3206	Protein TSSC1 partial	custom
NA	3207	Chaperone activity of bc1 complex-like mitochondrial partial	custom
NA	3208	Transmembrane protein 164 partial	custom
NA	3209	DNA-directed RNA polymerase I subunit RPA2 partial	custom

Supplemental Table 3 – continued from previous page

Arthropod Core ID	Spider Core ID	Putative Ortholog Description	Target Database
NA	3211	hypothetical protein X975 11667 partial	custom
NA	3212	hypothetical protein X975 08788 partial	custom
NA	3213	Cyclin-C partial	custom
NA	3214	PREDICTED: histone acetyltransferase KAT7-like	nr
NA	3215	Tricarboxylate transport protein mitochondrial partial	custom
89945	3216	NA	nr
NA	3220	putative 39S ribosomal protein L45 mitochondrial partial	custom
NA	3221	Protein RRNAD1 partial	custom
90310	3222	UDP-glucose 4-epimerase partial	custom
NA	3223	Motile sperm domain-containing protein 2 partial	custom
90261	3225	Guanylate kinase partial	custom
NA	3226	MFS-type transporter partial	custom
NA	3233	Abhydrolase domain-containing protein 14A partial	custom
NA	3234	Solute carrier family 35 member G1 partial	custom
NA	3235	Target of rapamycin complex subunit lst8 partial	custom
NA	3237	Abl interactor 1 partial	custom
NA	3239	Methionine synthase reductase partial	custom
NA	3240	DNA methyltransferase 1-associated protein 1 partial	custom
NA	3241	Protein bunched class 2/F isoform partial	custom
NA	3244	Mediator of RNA polymerase II transcription subunit 27-B partial	custom

Supplemental Table 3 – continued from previous page

Arthropod Core ID	Spider Core ID	Putative Ortholog Description	Target Database
NA	3245	Gamma-soluble NSF attachment protein partial	custom
89724	3246	Dual specificity mitogen-activated protein kinase kinase 4 partial	custom
89730	3247	CDK5 regulatory subunit-associated protein 3 partial	custom
NA	3248	Hemicentin-2 partial	custom
NA	3249	Thioredoxin-dependent peroxide reductase mitochondrial partial	custom
NA	3250	WD repeat-containing protein 48 partial	custom
NA	3251	Ubiquitin-conjugating enzyme E2 1 partial	custom
NA	3254	hypothetical protein X975 11864 partial	custom
NA	3255	putative GTP-binding protein 6 partial	custom
NA	3258	SWI/SNF-related matrix-associated actin-dependent regulator of chromatin subfamily A containing DEAD/H box 1 partial	custom
NA	3259	Glutamyl-tRNA(Gln) amidotransferase subunit B mitochondrial partial	custom
89837	3260	putative ATP-dependent RNA helicase DDX56 partial	custom
89697	3261	DNA excision repair protein haywire partial	custom
NA	3262	Nuclear receptor coactivator 5 partial	custom
NA	3263	Retinitis pigmentosa 9 protein partial	custom

Supplemental Table 3 – continued from previous page

Arthropod Core ID	Spider Core ID	Putative Ortholog Description	Target Database
NA	3264	U2 small nuclear ribonucleoprotein A partial	custom
NA	3265	Tetratricopeptide repeat protein 8 partial	custom
NA	3266	Serine/threonine-protein kinase 16 partial	custom
90611	3267	rRNA 2'-O-methyltransferase fibrillarin partial	custom
NA	3269	AP-3 complex subunit mu-1 partial	custom
89924	3270	ADP-ribosylation factor-related protein 1 partial	custom
NA	3271	hypothetical protein X975 19481 partial	custom
NA	3272	Coiled-coil domain-containing protein 134 partial	custom
NA	3273	UBX domain-containing protein 7 partial	custom
NA	3274	Casein kinase II subunit beta partial	custom
NA	3275	Hepatocyte nuclear factor 4-beta partial	custom
NA	3276	Nuclear pore complex protein Nup54 partial	custom
NA	3277	Trafficking protein particle complex subunit 10 partial	custom
NA	3279	Protein SMG5 partial	custom
90525	3280	Disks large-like protein partial	custom
NA	3281	Stomatin-like protein 2 partial	custom
NA	3282	Tumor protein p63-regulated 1-like protein partial	custom
NA	3285	Histone-lysine N-methyltransferase MLL3 partial	custom
NA	3286	Surfeit locus protein 1 partial	custom

Supplemental Table 3 – continued from previous page

Arthropod Core ID	Spider Core ID	Putative Ortholog Description	Target Database
NA	3289	Cysteine and histidine-rich domain-containing protein 1 partial	custom
NA	3290	TPPP family protein partial	custom
NA	3291	Geranylgeranyl transferase type-2 subunit beta partial	custom
NA	3292	putative phenylalanine–tRNA ligase mitochondrial partial	custom
NA	3293	Methyl-CpG-binding domain protein 2 partial	custom
NA	3294	Dwarfin sma-2 partial	custom
NA	3297	Apoptosis inhibitor 5 partial	custom
NA	3299	TBC1 domain family member 5 partial	custom
NA	3301	Zinc finger protein 474 partial	custom
NA	3303	Mitogen-activated protein kinase kinase kinase 7 partial	custom
NA	3304	Adipocyte plasma membrane-associated protein partial	custom
90367	3307	ATP-binding cassette sub-family D member 3 partial	custom
NA	3309	DNA excision repair protein ERCC-6-like protein partial	custom
NA	3314	DnaJ-like protein subfamily C member 10 partial	custom
NA	3315	Protein tumorous imaginal disc mitochondrial partial	custom
90414	3316	V-type proton ATPase proteolipid subunit partial	custom
NA	3317	Nucleolar complex protein 3-like protein partial	custom

Supplemental Table 3 – continued from previous page

Arthropod Core ID	Spider Core ID	Putative Ortholog Description	Target Database
NA	3318	Integrator complex subunit 7 partial	custom
NA	3320	Thioredoxin partial	custom
NA	3321	Tyrosine-protein phosphatase non-receptor type 23 partial	custom
NA	3322	E3 ubiquitin-protein ligase TRIM37 partial	custom
NA	3324	Tuberin partial	custom
NA	3326	Ubiquitin carboxyl-terminal hydrolase 34 partial	custom
NA	3330	hypothetical protein X975 25927 partial	custom
NA	3331	putative ATP-dependent RNA helicase DDX52 partial	custom
NA	3344	5-formyltetrahydrofolate cyclo-ligase partial	custom
NA	3349	Zinc finger protein GLI3 partial	custom
90524	3359	Histone H4 partial	custom
NA	3361	Cytochrome P450 3A24 partial	custom
NA	3367	Low-density lipoprotein receptor-related protein 2 partial	custom
90554	3370	Adenylosuccinate lyase partial	custom
NA	3371	Peptidyl-prolyl cis-trans isomerase B partial	custom
89955	3372	Histone H2A partial	custom
NA	3373	Nuclear valosin-containing protein-like protein partial	custom
90609	3374	elongation factor-1 gamma	nr
NA	3375	Epididymal secretory protein E1 partial	custom
NA	3377	P2X purinoceptor 4 partial	custom
NA	3378	Galectin-4 partial	custom

Supplemental Table 3 – continued from previous page

Arthropod Core ID	Spider Core ID	Putative Ortholog Description	Target Database
NA	3381	Vesicle-associated membrane protein 7 partial	custom
NA	3382	Aftiphilin partial	custom
89770	3383	Fructose-bisphosphate aldolase partial	custom
90620	3385	Nascent polypeptide-associated complex subunit alpha partial	custom
NA	3386	hypothetical protein X975 17660 partial	custom
NA	3387	hypothetical protein X975 04935 partial	custom
NA	3388	Ubiquitin-like protein 4A partial	custom
NA	3390	hypothetical protein X975 23193 partial	custom
NA	3391	CCA tRNA nucleotidyltransferase 1 mitochondrial partial	custom
90366	3392	39S ribosomal protein L12 mitochondrial partial	custom
90117	3393	Mitochondrial folate transporter/carrier partial	custom
NA	3395	DDB1- and CUL4-associated factor 11 partial	custom
NA	3396	Guanine nucleotide exchange factor MSS4 partial	custom
90108	3397	Phosphoglucomutase partial	custom
NA	3398	putative RISC-loading complex subunit partial	custom
NA	3399	hypothetical protein X975 20588 partial	custom
NA	3400	Protein FAM91A1 partial	custom
NA	3401	7SK snRNA methylphosphate capping enzyme partial	custom

Supplemental Table 3 – continued from previous page

Arthropod Core ID	Spider Core ID	Putative Ortholog Description	Target Database
89798	3402	Adaptin ear-binding coat-associated protein 1 partial	custom
NA	3404	PREDICTED: EF-hand domain-containing protein D2-like	nr
89805	3405	Actin-related protein 3 partial	custom
NA	3407	Kunitz-type protease inhibitor 1 partial	custom
NA	3409	hypothetical protein X975 22960 partial	custom
NA	3410	Nuclear pore complex protein Nup155 partial	custom
NA	3411	Protein asunder-like protein partial	custom
NA	3412	hypothetical protein X975 15341 partial	custom
NA	3413	Coiled-coil domain-containing protein 25 partial	custom
NA	3414	Protein FAM100B partial	custom
NA	3415	E3 ubiquitin-protein ligase TRIM33 partial	custom
NA	3417	WD repeat-containing protein 55 partial	custom
NA	3418	PREDICTED: cAMP-responsive element-binding protein-like 2	nr
90451	3419	Proteasomal ubiquitin receptor ADRM1 partial	custom
NA	3421	Trafficking protein particle complex subunit 4 partial	custom
NA	3422	Enoyl-CoA hydratase mitochondrial partial	custom
90134	3423	Pre-mRNA-processing factor 19 partial	custom
NA	3424	Prefoldin subunit 1 partial	custom

Supplemental Table 3 – continued from previous page

Arthropod Core ID	Spider Core ID	Putative Ortholog Description	Target Database
NA	3425	putative deoxyribose-phosphate aldolase partial	custom
NA	3427	hypothetical protein X975 17773 partial	custom
89820	3429	40S ribosomal protein S6 partial	custom
NA	3430	Zinc finger matrin-type protein 2 partial	custom
NA	3431	Polyglutamine-binding protein 1 partial	custom
NA	3432	Protein dpy-30-like protein partial	custom
NA	3433	Transcription initiation factor TFIID subunit 8 partial	custom
NA	3434	hypothetical protein X975 24418 partial	custom
90486	3435	Twinfilin-1 partial	custom
NA	3436	IST1-like protein partial	custom
90133	3437	AP-1 complex subunit sigma-2 partial	custom
NA	3439	Trafficking protein particle complex subunit 3 partial	custom
NA	3440	Perilipin-5 partial	custom
NA	3443	Mediator of RNA polymerase II transcription subunit 19 partial	custom
NA	3444	Transcription elongation factor B polypeptide 2 partial	custom
NA	3445	3-ketodihydrosphingosine reductase partial	custom
NA	3446	Magnesium-dependent phosphatase 1 partial	custom
89997	3447	NADH dehydrogenase [ubiquinone] 1 alpha subcomplex subunit 8 partial	custom
NA	3448	Density-regulated protein partial	custom

Supplemental Table 3 – continued from previous page

Arthropod Core ID	Spider Core ID	Putative Ortholog Description	Target Database
89796	3449	60S ribosomal protein L5-A partial	custom
NA	3451	Mediator of RNA polymerase II transcription subunit 29 partial	custom
NA	3452	Small nuclear ribonucleoprotein Sm D3 partial	custom
NA	3455	hypothetical protein X975 17159 partial	custom
NA	3456	mRNA turnover protein 4-like protein partial	custom
NA	3457	Solute carrier family 25 member 38 partial	custom
NA	3458	Heat shock protein beta-1 partial	custom
90407	3459	Chloride intracellular channel exc-4 partial	custom
NA	3460	Mucin-5AC partial	custom
NA	3461	Myosin regulatory light chain sqh partial	custom
NA	3462	Cytochrome c oxidase assembly protein COX15-like protein partial	custom
NA	3463	Tetratricopeptide repeat protein 35 partial	custom
NA	3464	Phosphate carrier protein mitochondrial partial	custom
NA	3465	hypothetical protein X975 12422 partial	custom
NA	3467	Na(+)/H(+) exchange regulatory cofactor NHE-RF2 partial	custom
90146	3468	Alpha-enolase partial	custom
89862	3469	hypothetical protein X975 24441 partial	custom
NA	3470	Sodium/potassium-transporting ATPase subunit beta-2 partial	custom
NA	3473	28S ribosomal protein S14 mitochondrial partial	custom

Supplemental Table 3 – continued from previous page

Arthropod Core ID	Spider Core ID	Putative Ortholog Description	Target Database
NA	3474	Activating transcription factor of chaperone partial	custom
NA	3475	Synaptosomal-associated protein 29 partial	custom
NA	3476	Charged multivesicular body protein 1a partial	custom
90577	3477	Signal recognition particle receptor subunit alpha partial	custom
NA	3478	Glycine–tRNA ligase partial	custom
90160	3479	DNA-directed RNA polymerases I II and III subunit RPABC1 partial	custom
89664	3480	Protein SEC13-like protein partial	custom
NA	3481	Cytosol aminopeptidase partial	custom
NA	3483	Zinc-binding alcohol dehydrogenase domain-containing protein 2 partial	custom
90271	3485	Trans-23-enoyl-CoA reductase partial	custom
NA	3487	Sorting nexin-6 partial	custom
NA	3488	Protein Tob2 partial	custom
NA	3490	Lysozyme 1 partial	custom
NA	3491	N(G)N(G)-dimethylarginine dimethylaminohydrolase 1 partial	custom
90180	3492	Hypothetical protein X975 00033 partial	custom
NA	3493	hypothetical protein X975 02279 partial	custom
NA	3494	hypothetical protein X975 00701 partial	custom
NA	3495	Hemicentin-1 partial	custom
NA	3496	Elongation of very long chain fatty acids protein partial	custom

Supplemental Table 3 – continued from previous page

Arthropod Core ID	Spider Core ID	Putative Ortholog Description	Target Database
NA	3497	SAGA-associated factor 29-like protein partial	custom
NA	3498	Protein midA-like protein mitochondrial partial	custom
NA	3500	Adenosine kinase partial	custom
NA	3502	Elongation factor G mitochondrial partial	custom
NA	3504	GTP-binding protein partial	custom
NA	3505	Transformer-2 protein-like protein beta partial	custom
NA	3507	Glycoprotein 3-alpha-L-fucosyltransferase A partial	custom
NA	3508	Inhibin beta E chain partial	custom
NA	3510	Ufm1-specific protease 2 partial	custom
90175	3512	Protein disulfide-isomerase A3 partial	custom
NA	3513	N-acetyl-D-glucosamine kinase partial	custom
90413	3514	Fructose-16-bisphosphatase 1 partial	custom
NA	3516	hypothetical protein X975 14350 partial	custom
NA	3517	hypothetical protein X975 04514 partial	custom
NA	3518	hypothetical protein X975 07313 partial	custom
NA	3519	Coiled-coil domain-containing protein 148 partial	custom
NA	3521	Mitotic-spindle organizing protein 2A partial	custom
NA	3522	Major facilitator superfamily domain-containing protein 4 partial	custom

Supplemental Table 3 – continued from previous page

Arthropod Core ID	Spider Core ID	Putative Ortholog Description	Target Database
89782	3523	General transcription factor 3C polypeptide 5 partial	custom
NA	3524	Cytidine deaminase partial	custom
NA	3527	E3 ubiquitin-protein ligase RFWD3 partial	custom
NA	3530	Ribosomal biogenesis protein LAS1L partial	custom
NA	3531	C-1-tetrahydrofolate synthase cytoplasmic partial	custom
NA	3533	Protein phosphatase 1 regulatory subunit 3C-B partial	custom
NA	3534	Protein TBRG4 partial	custom
NA	3535	Mitochondrial ubiquitin ligase activator of nfkb 1 partial	custom
NA	3540	Maspardin partial	custom
NA	3541	LanC-like protein 3-like protein partial	custom
NA	3542	Pseudouridylate synthase 7-like protein partial	custom
NA	3543	Protein NDRG3 partial	custom
NA	3544	28S ribosomal protein S27 mitochondrial partial	custom
NA	3545	Isopentenyl-diphosphate Delta-isomerase 1 partial	custom
NA	3546	WD repeat domain phosphoinositide-interacting protein 4 partial	custom
NA	3548	Hydroxyacylglutathione hydrolase mitochondrial partial	custom
NA	3552	U4/U6.U5 tri-snRNP-associated protein 1 partial	custom
NA	3553	Regulator of G-protein signaling 20 partial	custom

Supplemental Table 3 – continued from previous page

Arthropod Core ID	Spider Core ID	Putative Ortholog Description	Target Database
NA	3554	Protein PTCD3-like protein mitochondrial partial	custom
NA	3555	hypothetical protein X975 04622 partial	custom
NA	3556	Protein angel-like protein partial	custom
89777	3560	V-type proton AT-Pase subunit C partial	custom
NA	3561	Agmatinase mito-chondrial partial	custom
90060	3563	Conserved oligomeric Golgi complex subunit 3 partial	custom
NA	3564	Adenine phosphori-bosyltransferase par-tial	custom
NA	3565	hypothetical protein X975 23003 partial	custom
NA	3567	Target of Myb protein 1 partial	custom
NA	3568	DIS3-like exonucle-ase 1 partial	custom
NA	3572	Transcriptional adapter 3 partial	custom
NA	3573	Serine/threonine-protein kinase pelle partial	custom
NA	3574	Protein shuttle craft partial	custom
NA	3575	Baculoviral IAP repeat-containing protein 2 partial	custom
NA	3577	Mimitin mitochon-drial partial	custom
NA	3578	hypothetical protein X975 04962 partial	custom
NA	3579	putative OPA3-like protein partial	custom
NA	3580	Retinoid-inducible serine carboxypepti-dase partial	custom
NA	3581	Folylpolyglutamate synthase mitochon-drial partial	custom

Supplemental Table 3 – continued from previous page

Arthropod Core ID	Spider Core ID	Putative Ortholog Description	Target Database
89895	3582	Histone acetyltransferase type B catalytic subunit partial	custom
NA	3583	Peroxisomal trans-2-enoyl-CoA reductase partial	custom
NA	3584	hypothetical protein X975 19829 partial	custom
NA	3586	D-glucuronyl C5-epimerase partial	custom
NA	3587	Ubiquitin-conjugating enzyme E2 C partial	custom
NA	3589	Regulator of microtubule dynamics protein 1 partial	custom
NA	3590	Prenylcysteine oxidase partial	custom
NA	3591	Transmembrane protein 177 partial	custom
NA	3593	Coiled-coil domain-containing protein 91 partial	custom
NA	3594	Zinc transporter 7 partial	custom
NA	3597	Spermine synthase partial	custom
NA	3598	Mediator of RNA polymerase II transcription subunit 30 partial	custom
NA	3599	Protein PTHB1 partial	custom
NA	3600	Zinc finger HIT domain-containing protein 1 partial	custom
NA	3601	Dymeclin partial	custom
NA	3602	hypothetical protein X975 11969 partial	custom
NA	3603	S-formylglutathione hydrolase partial	custom
NA	3604	hypothetical protein X975 21314 partial	custom
NA	3606	Alpha- and gamma-adaptin-binding protein p34 partial	custom

Supplemental Table 3 – continued from previous page

Arthropod Core ID	Spider Core ID	Putative Ortholog Description	Target Database
90520	3607	Calcium-binding mitochondrial carrier protein SCaMC-2-A partial	custom
NA	3608	Glyoxylate reductase/hydroxypyruvate reductase partial	custom
NA	3610	Leucine-rich repeat neuronal protein 2 partial	custom
NA	3611	Stomatin-like protein 1 partial	custom
NA	3612	Lipoma-preferred partner partial	custom
NA	3613	Kinesin-like protein KIF20A partial	custom
90199	3614	Pre-mRNA-processing factor 17 partial	custom
NA	3615	Protein odr-4-like protein partial	custom
NA	3625	Transmembrane protein 39A partial	custom
90594	3627	Transcriptional regulator ATRX partial	custom
NA	3628	Carboxypeptidase N subunit 2 partial	custom
NA	3630	Autophagy-related protein 101 partial	custom
NA	3632	GRIP and coiled-coil domain-containing protein 2 partial	custom
NA	3633	hypothetical protein X975 01679 partial	custom
NA	3637	Nuclear distribution protein nudE-like protein partial	custom
NA	3640	5' exonuclease Apollo partial	custom
89667	3642	Serine/arginine-rich splicing factor 1 partial	custom
NA	3643	hypothetical protein X975 00727 partial	custom
NA	3644	putative 4-hydroxy-2-oxoglutarate aldolase mitochondrial partial	custom

Supplemental Table 3 – continued from previous page

Arthropod Core ID	Spider Core ID	Putative Ortholog Description	Target Database
NA	3645	decarboxylase, putative	nr
NA	3646	Protein kinase domain-containing protein cytoplasmic partial	custom
NA	3647	Small nuclear ribonucleoprotein-associated protein B partial	custom
89741	3648	Polyribonucleotide 5'-hydroxyl-kinase Clp1 partial	custom
NA	3649	Kelch domain-containing protein 2 partial	custom
NA	3650	39S ribosomal protein L3 mitochondrial partial	custom
90327	3652	Protein yippee-like 1 partial	custom
NA	3653	Gamma-glutamyl hydrolase partial	custom
NA	3656	mTERF domain-containing protein 1 mitochondrial partial	custom
NA	3657	DNA mismatch repair protein Mlh1 partial	custom
90531	3658	Suppressor of G2 allele of SKP1-like protein partial	custom
NA	3659	Zinc finger CCCH domain-containing protein 15-like protein partial	custom
NA	3660	hypothetical protein X975 24750 partial	custom
NA	3662	Structural maintenance of chromosomes protein 3 partial	custom
NA	3665	DNA polymerase kappa partial	custom
NA	3666	Clotting factor B partial	custom

Supplemental Table 3 – continued from previous page

Arthropod Core ID	Spider Core ID	Putative Ortholog Description	Target Database
NA	3667	DNA-directed RNA polymerases I II and III subunit RPABC3 partial	custom
NA	3668	E3 ubiquitin-protein ligase Topor partial	custom
NA	3670	Enhancer of mRNA-decapping protein 3 partial	custom
NA	3672	Thymidylate kinase partial	custom
NA	3673	60S ribosomal export protein NMD3 partial	custom
NA	3675	Tetratricopeptide repeat protein 21B partial	custom
NA	3677	Tudor domain-containing protein 3 partial	custom
NA	3678	Tetratricopeptide repeat protein 19 mitochondrial partial	custom
NA	3679	Transmembrane protein 165 partial	custom
NA	3681	Calcipressin-3 partial	custom
NA	3684	WD repeat-containing protein 70 partial	custom
NA	3685	Transcription initiation factor TFIID subunit 6 partial	custom
NA	3688	Serine/threonine-protein kinase 11-interacting protein partial	custom
NA	3691	Pre-mRNA-splicing factor 18 partial	custom
NA	3692	THO complex subunit 5-like protein partial	custom
90143	3695	Zinc finger protein 598 partial	custom
NA	3698	hypothetical protein X975 12731 partial	custom
NA	3700	putative E3 ubiquitin-protein ligase RNF144A partial	custom

Supplemental Table 3 – continued from previous page

Arthropod Core ID	Spider Core ID	Putative Ortholog Description	Target Database
NA	3701	GTPase Era mitochondrial partial	custom
NA	3703	hypothetical protein X975 04328 partial	custom
NA	3704	Beclin 1-associated autophagy-related key regulator partial	custom
NA	3706	PREDICTED: ataxin-7-like protein 3	nr
90349	3709	Nucleolar GTP-binding protein 1 partial	custom
NA	3710	L-gulonolactone oxidase partial	custom
90140	3712	U2 small nuclear ribonucleoprotein auxiliary factor subunit-related protein 1 partial	custom
90543	3713	Charged multivesicular body protein 6 partial	custom
90049	3715	NEDD8-activating enzyme E1 catalytic subunit partial	custom
90298	3716	Leucine carboxyl methyltransferase 1 partial	custom
NA	3717	hypothetical protein X975 12954 partial	custom
NA	3718	Inositol monophosphatase 3 partial	custom
NA	3719	Max-like protein X partial	custom
NA	3722	Protein Mo25 partial	custom
NA	3723	hypothetical protein X975 16963 partial	custom
NA	3724	Protein phosphatase methylesterase 1 partial	custom
NA	3725	Vacuolar protein sorting-associated protein 28-like protein partial	custom
NA	3726	Zinc finger protein-like 1 partial	custom
NA	3727	hypothetical protein X975 09423 partial	custom

Supplemental Table 3 – continued from previous page

Arthropod Core ID	Spider Core ID	Putative Ortholog Description	Target Database
NA	3729	ER degradation-enhancing alpha-mannosidase-like 1 partial	custom
NA	3730	Programmed cell death protein 10 partial	custom
NA	3731	Protein fem-1-like protein partial	custom
NA	3732	Zinc transporter ZIP4 partial	custom
NA	3733	Hippocampus abundant transcript 1 protein partial	custom
89942	3735	ADP-ribosylation factor-like protein 8B-A partial	custom
89766	3737	WD repeat domain phosphoinositide-interacting protein 3 partial	custom
NA	3739	Hypothetical protein arginine N-methyltransferase 10 partial	custom
NA	3740	Protein FAM164C partial	custom
NA	3744	hypothetical protein X975 13169 partial	custom
NA	3745	Sec1 family domain-containing protein 2 partial	custom
90334	3746	C2 domain-containing protein 2 partial	custom
NA	3747	Translin-associated protein X partial	custom
NA	3749	Protein ELYS partial	custom
NA	3751	Periodic tryptophan protein 2-like protein partial	custom
NA	3753	DnaJ-like protein subfamily C member 22 partial	custom
NA	3755	Calcineurin subunit B type 2 partial	custom

Supplemental Table 3 – continued from previous page

Arthropod Core ID	Spider Core ID	Putative Ortholog Description	Target Database
90555	3756	DnaJ-like protein subfamily B member 14 partial	custom
NA	3757	Fizzy-related protein-like protein partial	custom
NA	3759	E3 ubiquitin-protein ligase RBBP6 partial	custom
NA	3760	NA	nr
NA	3771	Regulation of nuclear pre-mRNA domain-containing protein 1A partial	custom
NA	3776	Acylglycerol kinase mitochondrial partial	custom
90066	3785	GDP-fucose protein O-fucosyltransferase 1 partial	custom
NA	3795	Exosome complex component RRP4 partial	custom
NA	3796	DNA ligase 1 partial	custom
NA	3801	Zinc finger protein 207 partial	custom
90153	3803	RNA-binding protein 5 partial	custom
NA	3805	hypothetical protein X975 08217 partial	custom
NA	3806	Voltage-dependent L-type calcium channel subunit beta-2 partial	custom
90316	3807	Mediator of RNA polymerase II transcription subunit 18 partial	custom
NA	3808	Short-chain dehydrogenase/reductase family 42E member 1 partial	custom
NA	3809	Coiled-coil domain-containing protein 97 partial	custom
NA	3810	HD domain-containing protein 2 partial	custom
NA	3811	Protein regulator of cytokinesis 1 partial	custom
NA	3812	Calcium-binding protein E63-1 partial	custom

Supplemental Table 3 – continued from previous page

Arthropod Core ID	Spider Core ID	Putative Ortholog Description	Target Database
NA	3813	Eukaryotic translation initiation factor 3 subunit J partial	custom
NA	3814	Cyclin-dependent kinase 17 partial	custom
NA	3816	WD and tetratricopeptide repeats protein 1 partial	custom
89700	3818	V-type proton ATPase subunit E partial	custom
NA	3819	Peripheral plasma membrane protein CASK partial	custom
NA	3820	Natterin-4 partial	custom
NA	3821	Deformed epidermal autoregulatory factor 1-like protein partial	custom
NA	3823	putative cytochrome P450 49a1 partial	custom
NA	3825	E3 ubiquitin-protein ligase RNF115 partial	custom
90374	3826	Heterogeneous nuclear ribonucleoprotein 87F partial	custom
NA	3828	Protein max partial	custom
90404	3829	hypothetical protein X975 03204 partial	custom
NA	3830	Peptidyl-tRNA hydrolase ICT1 mitochondrial partial	custom
NA	3831	Adipocyte plasma membrane-associated protein partial	custom
NA	3832	Ubiquitin carboxyl-terminal hydrolase isozyme L3 partial	custom
90415	3833	40S ribosomal protein S12 partial	custom
NA	3834	Microtubule-associated protein Jupiter partial	custom
NA	3835	Poly(A) RNA polymerase gld-2-like protein partial	custom

Supplemental Table 3 – continued from previous page

Arthropod Core ID	Spider Core ID	Putative Ortholog Description	Target Database
89973	3837	Transmembrane emp24 domain-containing protein bai partial	custom
NA	3838	Endoplasmic reticulum-Golgi intermediate compartment protein 2 partial	custom
NA	3839	Dipeptidyl peptidase 2 partial	custom
NA	3840	Serine/threonine-protein phosphatase 4 catalytic subunit partial	custom
NA	3841	N-acetylneuraminate lyase	nr
NA	3842	Superoxide dismutase [Cu-Zn] partial	custom
NA	3843	cAMP-regulated phosphoprotein 21 partial	custom
NA	3844	putative leucine–tRNA ligase mitochondrial partial	custom
NA	3845	Translocator protein partial	custom
NA	3846	Inosine-uridine preferring nucleoside hydrolase partial	custom
NA	3847	hypothetical protein X975 22772 partial	custom
NA	3848	Set1/Ash2 histone methyltransferase complex subunit ASH2 partial	custom
NA	3849	Ring canal kelch-like protein partial	custom
NA	3851	NF-kappa-B inhibitor-interacting Ras-like protein 2 partial	custom
NA	3852	Protein amnionless partial	custom
NA	3853	High mobility group protein 20A partial	custom
NA	3854	Thioredoxin mitochondrial partial	custom

Supplemental Table 3 – continued from previous page

Arthropod Core ID	Spider Core ID	Putative Ortholog Description	Target Database
NA	3856	Dihydrofolate reductase partial	custom
NA	3857	AN1-type zinc finger protein 2B partial	custom
NA	3858	Glutaredoxin-3 partial	custom
NA	3859	Flap endonuclease 1 partial	custom
NA	3863	Transcription factor IIIA partial	custom
90441	3865	40S ribosomal protein S3a partial	custom
NA	3867	Zinc finger FYVE domain-containing protein 19 partial	custom
NA	3869	Ras-related and estrogen-regulated growth inhibitor-like protein partial	custom
NA	3870	tRNA-splicing endonuclease subunit Sen34 partial	custom
NA	3871	Soma ferritin partial	custom
NA	3873	U7 snRNA-associated Sm-like protein LSm10 partial	custom
NA	3874	Isochorismatase domain-containing protein 2 mitochondrial partial	custom
NA	3875	Transcription initiation factor TFIID subunit 9B partial	custom
NA	3876	Synaptophysin-like protein 2 partial	custom
NA	3878	Transmembrane protein 130 partial	custom
NA	3879	Signal peptidase complex subunit 3 partial	custom
NA	3880	Poly(A)-specific ribonuclease PARN partial	custom
NA	3881	Proteasome subunit alpha type-4 partial	custom
NA	3882	Ras-related C3 botulinum toxin substrate 1 partial	custom

Supplemental Table 3 – continued from previous page

Arthropod Core ID	Spider Core ID	Putative Ortholog Description	Target Database
NA	3883	Protein twisted gastrulation partial	custom
NA	3886	Purine nucleoside phosphorylase partial	custom
NA	3887	Eukaryotic translation initiation factor 3 subunit K partial	custom
NA	3888	hypothetical protein X975 04362 partial	custom
NA	3889	hypothetical protein X975 23931 partial	custom
90379	3890	hypothetical protein X975 20185 partial	custom
NA	3891	Myosin regulatory light chain 2 partial	custom
NA	3892	Reticulocalbin-2 partial	custom
90371	3893	Glucosamine-6-phosphate isomerase 2 partial	custom
NA	3894	Ubiquitin-conjugating enzyme E2 Z partial	custom
NA	3895	Mitochondrial import inner membrane translocase subunit Tim23 partial	custom
NA	3896	Alkyldihydroxyacetonephosphate synthase peroxisomal partial	custom
NA	3897	Laminin subunit alpha-2 partial	custom
NA	3898	Lissencephaly-1-like protein partial	custom
NA	3901	Coiled-coil-helix-coiled-coil-helix domain-containing protein 3 mitochondrial partial	custom
89703	3902	40S ribosomal protein S13 partial	custom
89702	3903	NADH dehydrogenase [ubiquinone] iron-sulfur protein 7 mitochondrial partial	custom
90340	3904	60S ribosomal protein L4 partial	custom

Supplemental Table 3 – continued from previous page

Arthropod Core ID	Spider Core ID	Putative Ortholog Description	Target Database
NA	3905	PREDICTED: dna J homolog subfamily C member 24	nr
NA	3906	Potassium channel subfamily K member 18 partial	custom
90381	3907	Myotrophin partial	custom
89984	3908	DnaJ-like protein subfamily C member 9 partial	custom
NA	3909	Surfeit locus protein 6-like protein partial	custom
NA	3910	CD63 antigen partial	custom
NA	3911	Zinc finger protein 99 partial	custom
NA	3912	High mobility group protein DSP1 partial	custom
NA	3913	Glutaminyl-peptide cyclotransferase partial	custom
90328	3914	GrpE-like protein mitochondrial partial	custom
NA	3917	Cyclin-D1-binding protein 1-like protein partial	custom
90312	3918	heat shock protein mitochondrial partial	custom
NA	3919	hypothetical protein X975 06671 partial	custom
NA	3920	Cytochrome c1 heme protein mitochondrial partial	custom
NA	3922	Aminoacyl tRNA synthase complex-interacting multifunctional protein 1 partial	custom
NA	3923	Beta-sarcoglycan partial	custom
89969	3924	ATP synthase subunit alpha mitochondrial partial	custom
90454	3925	Protein CWC15-like protein partial	custom
90314	3926	Proteasome subunit alpha type-1 partial	custom
NA	3928	hypothetical protein X975 09269 partial	custom

Supplemental Table 3 – continued from previous page

Arthropod Core ID	Spider Core ID	Putative Ortholog Description	Target Database
NA	3929	KH domain-containing RNA-binding signal transduction-associated protein 3 partial	custom
NA	3930	Peroxiredoxin-4 partial	custom
NA	3931	hypothetical protein X975 10684 partial	custom
90262	3932	Dynactin subunit 2-A partial	custom
NA	3933	Estradiol 17-beta-dehydrogenase 12 partial	custom
NA	3935	Serine/arginine-rich splicing factor 4 partial	custom
89768	3936	Coiled-coil domain-containing protein 47 partial	custom
90351	3939	Ubiquitin carboxyl-terminal hydrolase 14 partial	custom
89691	3940	hypothetical protein X975 22147 partial	custom
NA	3941	Delta(35)-Delta(24)-dienoyl-CoA isomerase mitochondrial partial	custom
90437	3942	Clathrin light chain A partial	custom
90570	3946	Fumarylacetoacetase partial	custom
NA	3947	Protein charybde partial	custom
NA	3948	Chromobox protein-like protein partial	custom
90233	3949	ATP synthase subunit beta mitochondrial partial	custom
NA	3950	Glutathione S-transferase Mu 1 partial	custom
NA	3951	Ubiquinone biosynthesis protein COQ4-like protein mitochondrial partial	custom

Supplemental Table 3 – continued from previous page

Arthropod Core ID	Spider Core ID	Putative Ortholog Description	Target Database
NA	3952	Calbindin-32 partial	custom
NA	3954	28S ribosomal protein S5 mitochondrial partial	custom
90401	3955	S-methyl-5'-thioadenosine phosphorylase partial	custom
NA	3957	hypothetical protein X975 14961 partial	custom
NA	3958	Alanine–glyoxylate aminotransferase 2-like 1 partial	custom
NA	3959	Syndecan partial	custom
90568	3963	Fumarate hydratase mitochondrial partial	custom
NA	3964	putative ankyrin repeat protein partial	custom
89871	3965	Ribonucleoside-diphosphate reductase subunit M2 partial	custom
NA	3966	NFU1 iron-sulfur cluster scaffold-like protein mitochondrial partial	custom
NA	3967	F-box only protein 9 partial	custom
NA	3968	Factor VIII intron 22 protein partial	custom
90083	3969	Electron transfer flavoprotein subunit beta partial	custom
89996	3970	Translocating chain-associated membrane protein 1 partial	custom
NA	3971	hypothetical protein X975 04856 partial	custom
89679	3974	Nuclear pore membrane glycoprotein 210 partial	custom
90069	3977	Succinyl-CoA ligase [ADP/GDP-forming] subunit alpha mitochondrial partial	custom
NA	3981	26S proteasome non-ATPase regulatory subunit 13 partial	custom

Supplemental Table 3 – continued from previous page

Arthropod Core ID	Spider Core ID	Putative Ortholog Description	Target Database
NA	3987	Trafficking protein particle complex subunit 2-like protein partial	custom
NA	3988	60S ribosomal protein L23a partial	custom
NA	3989	Counting factor associated protein D partial	custom
NA	3992	Ras-related protein Rab-22A partial	custom
NA	3993	Exosome component 10 partial	custom
90539	3994	hypothetical protein X975 22281 partial	custom
90035	3995	26S proteasome non-ATPase regulatory subunit 9 partial	custom
NA	3996	Synergin gamma partial	custom
NA	3998	hypothetical protein X975 25529 partial	custom
NA	3999	Synaptotagmin-like protein 5 partial	custom
NA	4000	Solute carrier family 25 member 39 partial	custom
NA	4001	Prenylated Rab acceptor protein 1 partial	custom
NA	4003	hypothetical protein X975 15992 partial	custom
NA	4004	hypothetical protein X975 21126 partial	custom
NA	4005	60S ribosomal protein L10a-2 partial	custom
NA	4006	Inhibitor of growth protein 3 partial	custom
90077	4008	Galactosylgalactosylxylosylprotein 3-beta-glucuronosyltransferase 3 partial	custom
NA	4009	Protein prenyltransferase alpha subunit repeat-containing protein 1-B partial	custom
NA	4010	hypothetical protein X975 00334 partial	custom

Supplemental Table 3 – continued from previous page

Arthropod Core ID	Spider Core ID	Putative Ortholog Description	Target Database
89684	4012	NAD kinase domain-containing protein 1 partial	custom
NA	4013	Ribosomal RNA-processing protein 7-like protein partial	custom
NA	4015	Cyclin-H partial	custom
NA	4017	Solute carrier family 25 member 36 partial	custom
NA	4018	Heat shock protein beta-1 partial	custom
NA	4019	Pentatricopeptide repeat-containing protein 2 partial	custom
NA	4020	Chloride intracellular channel exl-1 partial	custom
NA	4021	LETM1 domain-containing protein 1 partial	custom
NA	4022	Exosome complex component RRP42 partial	custom
89736	4023	BTB/POZ domain-containing protein 3 partial	custom
NA	4024	Prostaglandin E synthase 2 partial	custom
NA	4026	Male-specific lethal 3-like protein partial	custom
NA	4027	hypothetical protein X975 23947 partial	custom
NA	4028	Sesquipedalian-1 partial	custom
NA	4029	Caveolin-1 partial	custom
NA	4030	Syntaxin-16 partial	custom
NA	4031	39S ribosomal protein L44 mitochondrial partial	custom
NA	4032	hypothetical protein X975 10850 partial	custom
90491	4033	Macrophage erythroblast attacher partial	custom
NA	4034	Dual specificity mitogen-activated protein kinase kinase 6 partial	custom

Supplemental Table 3 – continued from previous page

Arthropod Core ID	Spider Core ID	Putative Ortholog Description	Target Database
NA	4036	RILP-like protein-like protein partial	custom
NA	4039	Bone morphogenetic protein 3B partial	custom
NA	4040	DnaJ-like protein subfamily B member 9 partial	custom
NA	4041	ATP-binding cassette sub-family G member 5 partial	custom
NA	4042	N-alpha-acetyltransferase 30 partial	custom
90047	4044	Transmembrane emp24 domain-containing protein 2 partial	custom
NA	4046	Translationally-controlled tumor protein homolog, TCTP	nr
NA	4048	hypothetical protein X975 08140 partial	custom
NA	4049	Eukaryotic translation elongation factor 1 epsilon-1 partial	custom
NA	4050	Importin-13 partial	custom
NA	4052	Transcription elongation factor B polypeptide 1 partial	custom
NA	4053	Actin-binding Rho-activating protein partial	custom
NA	4054	Tudor domain-containing protein 7 partial	custom
NA	4057	Protein N-terminal asparagine amidohydrolase partial	custom
NA	4058	Ragulator complex protein LAMTOR2 partial	custom
90195	4059	39S ribosomal protein L50 mitochondrial partial	custom
NA	4060	Ras-like GTP-binding protein rhoA partial	custom

Supplemental Table 3 – continued from previous page

Arthropod Core ID	Spider Core ID	Putative Ortholog Description	Target Database
NA	4061	39S ribosomal protein L1 mitochondrial partial	custom
NA	4062	hypothetical protein X975 20218 partial	custom
89938	4063	Golgi to ER traffic protein 4-like protein partial	custom
89647	4065	Eukaryotic translation initiation factor 2 subunit 2 partial	custom
NA	4066	E3 ubiquitin-protein ligase TTC3 partial	custom
NA	4067	Gamma-glutamylcyclotransferase partial	custom
NA	4068	hypothetical protein X975 01638 partial	custom
90635	4069	Maleylacetoacetate isomerase partial	custom
NA	4070	Protein SERAC1 partial	custom
NA	4071	hypothetical protein X975 12365 partial	custom
NA	4072	hypothetical protein X975 02718 partial	custom
NA	4073	U1 small nuclear ribonucleoprotein A partial	custom
NA	4074	Translation initiation factor eIF-2B subunit beta partial	custom
NA	4075	COMM domain-containing protein 7 partial	custom
NA	4076	Mitochondrial fission 1 protein partial	custom
NA	4082	Short transient receptor hypothetical channel 5 partial	custom
NA	4083	Exosome complex component RRP45 partial	custom
NA	4084	COP9 signalosome complex subunit 6 partial	custom
NA	4085	Protein GTLF3B partial	custom

Supplemental Table 3 – continued from previous page

Arthropod Core ID	Spider Core ID	Putative Ortholog Description	Target Database
NA	4087	LanC-like protein 2 partial	custom
NA	4088	Nucleoporin NDC1 partial	custom
90259	4089	Spermatogenesis-associated protein 20 partial	custom
90307	4091	U3 small nucleolar RNA-interacting protein 2 partial	custom
NA	4095	WD repeat-containing protein 13 partial	custom
NA	4096	Cyclin-dependent kinase 20 partial	custom
NA	4097	Translation initiation factor eIF-2B subunit alpha partial	custom
NA	4098	hypothetical protein X975 22635 partial	custom
NA	4100	Cyclin-K partial	custom
NA	4101	Enoyl-CoA hydratase domain-containing protein 3 mitochondrial partial	custom
NA	4103	hypothetical protein X975 15478 partial	custom
NA	4104	SUMO-activating enzyme subunit 2 partial	custom
NA	4105	PREDICTED: protein UXT-like	nr
NA	4106	Mitochondrial inner membrane protein OXA1L partial	custom
90339	4107	Phosphorylase b kinase gamma catalytic chain testis/liver isoform partial	custom
NA	4108	Mismatch repair endonuclease PMS2 partial	custom
NA	4109	Rho guanine nucleotide exchange factor 26 partial	custom
NA	4112	ATP-dependent DNA helicase Q1 partial	custom
NA	4114	Smoothelin partial	custom

Arthropod Core ID	Spider Core ID	Putative Ortholog Description	Target Database
NA	4115	Protein Muted-like protein partial	custom
NA	4121	Leucine-rich repeat neuronal protein 2 partial	custom
NA	4123	hypothetical protein X975 23168 partial	custom
NA	4126	tRNA-specific adenosine deaminase 2 partial	custom
NA	4131	Leucine-rich PPR motif-containing protein mitochondrial partial	custom
NA	4132	Band 4.1-like protein 3 partial	custom
NA	4133	Nuclear pore complex protein Nup153 partial	custom
NA	4135	Acyloxyacyl hydrolase partial	custom
NA	4136	putative E3 ubiquitin-protein ligase MYCBP2 partial	custom
90003	4138	Ribose-5-phosphate isomerase partial	custom
90474	4139	Calcium-binding protein p22 partial	custom
NA	4142	Protein RRP5-like protein partial	custom
NA	4143	putative sodium/potassium-transporting ATPase subunit beta-3 partial	custom
NA	4145	putative tRNA pseudouridine synthase 2 partial	custom
NA	4146	Ras-related protein Rab-40C partial	custom
NA	4147	39S ribosomal protein L32 mitochondrial partial	custom
NA	4148	GPI transamidase component PIG-S partial	custom
NA	4149	Pinin partial	custom
NA	4150	Palmitoyl-protein thioesterase 1 partial	custom

Supplemental Table 3 – continued from previous page

Arthropod Core ID	Spider Core ID	Putative Ortholog Description	Target Database
NA	4151	Elongator complex protein 4 partial	custom
90229	4152	PCI domain-containing protein 2 partial	custom
NA	4153	Cyclin-dependent kinase 9 partial	custom
90598	4155	N-acetylgalactosamine kinase partial	custom
NA	4156	Glucosylceramidase partial	custom
NA	4157	p21-activated protein kinase-interacting protein 1-like protein partial	custom
89875	4158	hypothetical protein X975 11894 partial	custom
NA	4159	putative imidazolone-propionase partial	custom
NA	4161	Adenosine deaminase-like protein partial	custom
90163	4163	Bifunctional polynucleotide phosphatase/kinase partial	custom
90010	4164	Protein l(2)37Cc partial	custom
NA	4165	Wee1-like protein kinase partial	custom
NA	4166	Actin-related protein 8 partial	custom
NA	4167	RING finger protein 170 partial	custom
NA	4168	hypothetical protein X975 10461 partial	custom
NA	4169	1-acyl-sn-glycerol-3-phosphate acyltransferase delta partial	custom
NA	4171	hypothetical protein X975 15720 partial	custom
NA	4172	Solute carrier family 35 member E2 partial	custom
89781	4174	Proline synthase co-transcribed bacterial-like protein partial	custom

Supplemental Table 3 – continued from previous page

Arthropod Core ID	Spider Core ID	Putative Ortholog Description	Target Database
NA	4175	Histidine protein methyltransferase 1-like protein partial	custom
NA	4176	Protein DDI1-like protein partial	custom
NA	4177	Protein CNPPD1 partial	custom
NA	4178	Protein IMPACT-A partial	custom
NA	4179	Protein RMD5-like protein partial	custom
NA	4181	putative homeodomain transcription factor partial	custom
90369	4183	DNA-directed RNA polymerases I II and III subunit RPABC2 partial	custom
NA	4184	Sedoheptulokinase partial	custom
NA	4185	Adenylyl cyclase-associated protein 2 partial	custom
NA	4187	60S ribosomal protein L13 partial	custom
90519	4188	40S ribosomal protein S5 partial	custom
NA	4189	28S ribosomal protein S18c mitochondrial partial	custom
NA	4190	Centromere/kinetochore protein zw10-like protein partial	custom
NA	4191	RNA-binding protein 8A partial	custom
NA	4192	Large subunit GTPase 1-like protein partial	custom
NA	4193	G patch domain and KOW motifs-containing protein partial	custom
90581	4195	Mitochondrial intermediate peptidase partial	custom
NA	4196	Suppressor of cytokine signaling 6 partial	custom

Supplemental Table 3 – continued from previous page

Arthropod Core ID	Spider Core ID	Putative Ortholog Description	Target Database
90222	4197	Protein l(2)37Cc partial	custom
NA	4198	Protein arginine N-methyltransferase 6 partial	custom
NA	4201	Ankyrin repeat domain-containing protein 27 partial	custom
NA	4202	CCAAT/enhancer-binding protein alpha partial	custom
NA	4203	Nucleolar MIF4G domain-containing protein 1 partial	custom
NA	4204	39S ribosomal protein L19 mitochondrial partial	custom
NA	4207	hypothetical protein X975 02247 partial	custom
90323	4209	SUMO-conjugating enzyme UBC9 partial	custom
NA	4212	UDP-N-acetylglucosamine transporter partial	custom
NA	4213	Digestive organ expansion factor-like protein partial	custom
NA	4216	Mitochondrial import inner membrane translocase subunit Tim21 partial	custom
NA	4217	General transcription factor IIE subunit 2 partial	custom
NA	4218	ADP-ribosylation factor-binding protein GGA1 partial	custom
NA	4220	Leucine-rich repeat-containing protein 8A partial	custom
NA	4221	Uracil phosphoribosyltransferase-like protein partial	custom
NA	4226	Harmonin partial	custom
NA	4227	Estradiol 17-beta-dehydrogenase 8 partial	custom

Supplemental Table 3 – continued from previous page

Arthropod Core ID	Spider Core ID	Putative Ortholog Description	Target Database
NA	4228	hypothetical protein X975 11319 partial	custom
NA	4229	Ras-related protein Rab-8A partial	custom
NA	4231	Presenilins-associated rhomboid-like protein mitochondrial partial	custom
NA	4232	Retinol dehydrogenase 14 partial	custom
NA	4233	Heat shock factor protein 1 partial	custom
NA	4234	Ceramide kinase partial	custom
NA	4236	Fringe glycosyltransferase partial	custom
NA	4237	Vacuole membrane protein 1 partial	custom
NA	4238	Glutamate–cysteine ligase regulatory subunit partial	custom
NA	4246	Serine/threonine-protein kinase LMTK1 partial	custom
NA	4248	Transcriptional repressor p66 alpha partial	custom
NA	4249	putative nuclease HARBI1 partial	custom
NA	4257	Short spindle protein 4 partial	custom
NA	4258	Growth arrest and DNA damage-inducible proteins-interacting protein 1 partial	custom
NA	4259	Vacuolar protein sorting-associated protein 37B partial	custom
NA	4260	Paramyosin partial	custom
NA	4261	TWiK family of potassium channels protein 18 partial	custom
NA	4263	Transmembrane and TPR repeat-containing protein partial	custom

Supplemental Table 3 – continued from previous page

Arthropod Core ID	Spider Core ID	Putative Ortholog Description	Target Database
NA	4268	Homeobox protein Nkx-2.3 partial	custom
NA	4270	Ras-related GTP-binding protein A partial	custom
NA	4273	hypothetical protein X975 10671 partial	custom
NA	4275	Integrator complex subunit 9 partial	custom
NA	4277	hypothetical protein X975 12010 partial	custom
NA	4279	Epidermal retinol dehydrogenase 2 partial	custom
NA	4285	Conserved oligomeric Golgi complex subunit 8 partial	custom
NA	4290	Guanine nucleotide-binding protein-like 1 partial	custom
89669	4295	Nitric oxide synthase-interacting protein partial	custom
NA	4299	Peptidyl-prolyl cis-trans isomerase FKBP1A partial	custom
NA	4312	Telomere length regulation protein TEL2-like protein partial	custom
90224	4313	Nuclear pore complex protein Nup133 partial	custom
NA	4314	hypothetical protein X975 06017 partial	custom
NA	4315	Retinoblastoma-like protein 1 partial	custom
NA	4316	Inhibitor of growth protein 4 partial	custom
NA	4317	Bardet-Biedl syndrome 1 protein partial	custom
NA	4320	Eukaryotic translation initiation factor 3 subunit I partial	custom
NA	4321	17-beta-hydroxysteroid dehydrogenase type 6 partial	custom

Supplemental Table 3 – continued from previous page

Arthropod Core ID	Spider Core ID	Putative Ortholog Description	Target Database
NA	4322	Nuclear inhibitor of protein phosphatase 1 partial	custom
90556	4323	Leucine-rich repeat-containing protein 58 partial	custom
NA	4324	immunoglobulin-binding protein 1	nr
NA	4325	ubiquinone biosynthesis O-methyltransferase, mitochondrial-like	nr
NA	4328	Importin subunit alpha-3 partial	custom
NA	4329	Ubiquitin domain-containing protein UBFD1 partial	custom
NA	4331	hypothetical protein X975 26638 partial	custom
NA	4334	Mediator of RNA polymerase II transcription subunit 22 partial	custom
NA	4335	Ataxin-3 partial	custom
NA	4337	hypothetical protein X975 04970 partial	custom
89935	4339	putative malonyl-CoA-acyl carrier protein transacylase mitochondrial partial	custom
NA	4342	rRNA-processing protein UTP23-like protein partial	custom
NA	4343	M-phase phosphoprotein 6 partial	custom
89887	4346	GDP-mannose 46 dehydratase partial	custom
NA	4348	Methylosome protein 50 partial	custom
NA	4349	Neuronal acetylcholine receptor subunit alpha-2 partial	custom
NA	4352	COMM domain-containing protein 9 partial	custom

Supplemental Table 3 – continued from previous page

Arthropod Core ID	Spider Core ID	Putative Ortholog Description	Target Database
NA	4354	Uridine-cytidine kinase 2-B partial	custom
90306	4355	ATP synthase subunit O mitochondrial partial	custom
NA	4356	Heterogeneous nuclear ribonucleoprotein 27C partial	custom
NA	4357	hypothetical protein X975 12086 partial	custom
NA	4361	60S ribosomal protein L22 partial	custom
NA	4362	Protein disulfide-isomerase partial	custom
NA	4363	antigen AG231 partial	custom
NA	4364	NADH dehydrogenase [ubiquinone] 1 alpha subcomplex assembly factor 3 partial	custom
NA	4365	Centrin-1 partial	custom
NA	4366	Protein KTI12-like protein partial	custom
NA	4368	Protein YIPF6 partial	custom
NA	4370	39S ribosomal protein L16 mitochondrial partial	custom
90648	4371	RWD domain-containing protein 1 partial	custom
NA	4374	PDZ and LIM domain protein 7 partial	custom
90419	4375	60S ribosomal protein L23 partial	custom
NA	4376	Proteasome maturation protein partial	custom
NA	4377	28S ribosomal protein S30 mitochondrial partial	custom
NA	4378	28S ribosomal protein S11 mitochondrial partial	custom
NA	4379	Peptidyl-glycine alpha-amidating monooxygenase partial	custom

Supplemental Table 3 – continued from previous page

Arthropod Core ID	Spider Core ID	Putative Ortholog Description	Target Database
NA	4380	Macrophage migration inhibitory factor partial	custom
NA	4381	NADPH-dependent diflavin oxidoreductase 1 partial	custom
89911	4382	hypothetical protein X975 07425 partial	custom
NA	4383	Ubiquitin carboxyl-terminal hydrolase 30 partial	custom
NA	4384	RanBP-type and C3HC4-type zinc finger-containing protein 1 partial	custom
NA	4385	hypothetical protein X975 25380 partial	custom
NA	4387	Superoxide dismutase [Mn] 1 partial	custom
90136	4388	Mitogen-activated protein kinase 14 partial	custom
NA	4389	FERM domain-containing protein 8 partial	custom
90001	4390	Bystin partial	custom
90061	4391	39S ribosomal protein L2 mitochondrial partial	custom
NA	4392	Phosducin-like protein partial	custom
NA	4393	hypothetical protein X975 07699 partial	custom
NA	4395	hypothetical protein X975 10147 partial	custom
NA	4396	THO complex subunit 7-like protein partial	custom
NA	4397	Coiled-coil-helix-coiled-coil-helix domain-containing protein 2 mitochondrial partial	custom
NA	4398	hypothetical protein X975 01115 partial	custom
NA	4399	Ubiquitin-conjugating enzyme E2 H partial	custom

Supplemental Table 3 – continued from previous page

Arthropod Core ID	Spider Core ID	Putative Ortholog Description	Target Database
NA	4401	Autophagy-related protein 16-1 partial	custom
NA	4404	Pre-mRNA-splicing factor SPF27 partial	custom
NA	4405	NEDD8-conjugating enzyme UBE2F partial	custom
NA	4408	PREDICTED: protein FAM177A1-like	nr
90147	4409	Peptidyl-prolyl cis-trans isomerase-like 3 partial	custom
NA	4410	Headcase protein-like protein partial	custom
NA	4411	Zinc finger CCHC domain-containing protein 9 partial	custom
NA	4412	Testis-expressed sequence 264 protein partial	custom
NA	4414	Coenzyme Q-binding protein COQ10-like protein mitochondrial partial	custom
90254	4415	putative U3 small nucleolar RNA-associated protein 11 partial	custom
90593	4416	40S ribosomal protein S2 partial	custom
90592	4417	Prefoldin subunit 4 partial	custom
NA	4418	60S ribosomal protein L35a partial	custom
NA	4421	Protein LLP-like protein partial	custom
89654	4423	phosphohistidine phosphatase partial	custom
NA	4424	Myosin regulatory light chain sqh partial	custom
NA	4425	Deoxyribodipyrimidine photo-lyase partial	custom
NA	4426	Quinone oxidoreductase-like protein 2 partial	custom

Supplemental Table 3 – continued from previous page

Arthropod Core ID	Spider Core ID	Putative Ortholog Description	Target Database
NA	4427	putative methylthioribulose-1-phosphate dehydratase partial	custom
NA	4429	hypothetical protein X975 10007 partial	custom
NA	4432	PREDICTED: ovarian cancer-associated gene 2 protein homolog	nr
NA	4433	Leucine-rich repeat-containing protein 57 partial	custom
90225	4434	60S ribosomal protein L24 partial	custom
NA	4435	Proteasome subunit beta type-4 partial	custom
89725	4436	Uridine 5'-monophosphate synthase partial	custom
90230	4437	DnaJ-like protein subfamily B member 11 partial	custom
NA	4438	Phosducin-like protein 3 partial	custom
NA	4439	Ribosome maturation protein SBDS partial	custom
NA	4440	hypothetical protein X975 09445 partial	custom
NA	4441	MOB kinase activator 1B partial	custom
NA	4443	Prefoldin subunit 6 partial	custom
NA	4444	39S ribosomal protein L15 mitochondrial partial	custom
NA	4445	Peptidyl-prolyl cis-trans isomerase partial	custom
90071	4446	Malate dehydrogenase cytoplasmic partial	custom
89843	4448	DNA replication licensing factor mcm5-A partial	custom
NA	4449	Proteasome subunit alpha type-3 partial	custom

Supplemental Table 3 – continued from previous page

Arthropod Core ID	Spider Core ID	Putative Ortholog Description	Target Database
NA	4450	putative ribosome biogenesis protein RLP24 partial	custom
89804	4451	putative pyruvate dehydrogenase E1 component subunit alpha mitochondrial partial	custom
90248	4452	Pantothenate kinase 1 partial	custom
90603	4454	60S ribosomal protein L8 partial	custom
NA	4456	hypothetical protein X975 07045 partial	custom
NA	4458	Vesicular integral-membrane protein partial	custom
NA	4459	NADH dehydrogenase [ubiquinone] 1 alpha subcomplex subunit 7 partial	custom
NA	4463	Leucine zipper transcription factor-like protein 1 partial	custom
90578	4464	Protein DJ-1 partial	custom
NA	4465	Cytochrome b5 partial	custom
NA	4466	Dual specificity protein phosphatase 19 partial	custom
NA	4467	PREDICTED: rho GDP-dissociation inhibitor 1-like	nr
NA	4470	Dynactin subunit 6 partial	custom
NA	4471	Ras-related protein Rab-10 partial	custom
89665	4472	V-type proton ATPase subunit F 1 partial	custom
NA	4473	Vacuolar protein sorting-associated protein 29 partial	custom
NA	4474	Syntaxin-8 partial	custom
NA	4479	Sarcoplasmic calcium-binding proteins I III and IV partial	custom

Supplemental Table 3 – continued from previous page

Arthropod Core ID	Spider Core ID	Putative Ortholog Description	Target Database
NA	4480	Charged multivesicular body protein 2b partial	custom
90653	4481	Proteasome subunit alpha type-7-like protein partial	custom
NA	4483	hypothetical protein X975 07392 partial	custom
NA	4484	Complement factor B partial	custom
NA	4485	28S ribosomal protein S7 mitochondrial partial	custom
NA	4487	Saccharopine dehydrogenase-like oxidoreductase partial	custom
89704	4488	Protein YIF1B-A partial	custom
NA	4489	Serine/threonine-protein phosphatase 4 catalytic subunit partial	custom
NA	4490	Ras-related protein partial	custom
NA	4491	Protein LSM12-like protein partial	custom
90202	4492	Coatomer subunit epsilon partial	custom
90350	4493	Growth hormone-inducible transmembrane protein partial	custom
NA	4495	Succinate-semialdehyde dehydrogenase mitochondrial partial	custom
NA	4496	hypothetical protein X975 05777 partial	custom
89878	4497	Ubiquitin domain-containing protein 1 partial	custom
NA	4499	DNA replication licensing factor mcm4-A partial	custom
NA	4500	DNA polymerase epsilon subunit 3 partial	custom

Supplemental Table 3 – continued from previous page

Arthropod Core ID	Spider Core ID	Putative Ortholog Description	Target Database
NA	4501	Isoamyl acetate-hydrolyzing esterase 1-like protein partial	custom
NA	4503	MACRO domain-containing protein 2 partial	custom
NA	4504	Plasma kallikrein partial	custom
NA	4505	Fibrillin-1 partial	custom
NA	4510	Caprin-1 partial	custom
NA	4514	Transmembrane protease serine 9 partial	custom
NA	4515	Phosphomannomutase 2 partial	custom
89779	4517	Neuroguidin-A partial	custom
NA	4519	putative glucosamine 6-phosphate N-acetyltransferase partial	custom
NA	4521	28S ribosomal protein S9 mitochondrial partial	custom
NA	4522	60S ribosomal protein L14 partial	custom
90600	4524	Iron-sulfur cluster assembly enzyme ISCU mitochondrial partial	custom
90174	4525	Signal recognition particle receptor subunit beta partial	custom
NA	4526	Peptidyl-prolyl cis-trans isomerase FKBP12 partial	custom
NA	4528	Something about silencing protein 10 partial	custom
NA	4529	putative protein-like protein partial	custom
NA	4530	Pre-rRNA-processing protein esf2 partial	custom
90322	4531	Peptidyl-prolyl cis-trans isomerase-like 2 partial	custom
NA	4532	putative 28S ribosomal protein S26 mitochondrial partial	custom

Supplemental Table 3 – continued from previous page

Arthropod Core ID	Spider Core ID	Putative Ortholog Description	Target Database
NA	4533	Troponin I partial	custom
NA	4534	PREDICTED: mediator of RNA polymerase II transcription subunit 21-like	nr
90286	4535	Ribosome production factor 1 partial	custom
NA	4537	Transmembrane protein 53 partial	custom
NA	4538	Zinc finger FYVE domain-containing protein 21 partial	custom
NA	4539	putative aarF domain-containing protein kinase 5 partial	custom
89863	4540	COP9 signalosome complex subunit 4 partial	custom
NA	4541	Replication factor C subunit 3 partial	custom
NA	4542	3-hydroxyacyl-CoA dehydratase partial	custom
NA	4544	Unconventional prefoldin RPB5 interactor 1 partial	custom
NA	4545	U4/U6 small nuclear ribonucleoprotein Prp4 partial	custom
NA	4546	hypothetical protein X975 16775 partial	custom
NA	4547	Ubiquitin-like protein 3 partial	custom
NA	4548	Protein wntless partial	custom
NA	4549	Transmembrane protein 55B partial	custom
NA	4550	COMM domain-containing protein 10 partial	custom
NA	4551	LSM domain-containing protein 1 partial	custom
90076	4553	Thioredoxin-related transmembrane protein 2-like protein partial	custom
NA	4554	Protein BANP partial	custom

Supplemental Table 3 – continued from previous page

Arthropod Core ID	Spider Core ID	Putative Ortholog Description	Target Database
NA	4555	Diphthamide biosynthesis protein 1 partial	custom
NA	4559	GPN-loop GTPase 2 partial	custom
NA	4561	Cleavage and polyadenylation specificity factor subunit 5 partial	custom
NA	4562	Translation initiation factor eIF-2B subunit gamma partial	custom
NA	4563	Serum response factor partial	custom
NA	4564	Zinc finger protein Noc partial	custom
NA	4565	Succinate dehydrogenase [ubiquinone] iron-sulfur subunit mitochondrial partial	custom
NA	4566	NEDD8-conjugating enzyme Ubc12 partial	custom
89723	4568	hypothetical protein X975 00032 partial	custom
NA	4571	Cytochrome c partial	custom
NA	4572	hypothetical protein X975 22559 partial	custom
NA	4576	Protein CDV3-like protein partial	custom
NA	4577	NudC domain-containing protein 3 partial	custom
NA	4579	hypothetical protein X975 05206 partial	custom
89780	4580	Pyridoxine-5'-phosphate oxidase partial	custom
NA	4582	E3 ubiquitin-protein ligase NRDP1 partial	custom
NA	4583	ATPase ASNA1-like protein partial	custom
NA	4584	F-box/WD repeat-containing protein 9 partial	custom
NA	4586	39S ribosomal protein L30 mitochondrial partial	custom

Supplemental Table 3 – continued from previous page

Arthropod Core ID	Spider Core ID	Putative Ortholog Description	Target Database
NA	4587	Histone deacetylase 11 partial	custom
NA	4588	TBC1 domain family member 20 partial	custom
NA	4589	Acyl carrier protein mitochondrial partial	custom
NA	4590	NAD-dependent lysine demalonylase and desuccinylase sirtuin-5 mitochondrial partial	custom
90329	4591	Partner of Y14 and mago partial	custom
NA	4592	Copper homeostasis protein CutC partial	custom
NA	4593	Transmembrane protein 198-B partial	custom
NA	4594	Abhydrolase domain-containing protein 11 partial	custom
NA	4595	Transcription factor BTF3-like protein partial	custom
NA	4596	PREDICTED: protein SCO1 homolog, mitochondrial-like isoform	nr
NA	4597	Mitochondrial carrier-like protein partial	custom
NA	4598	Septin-1 partial	custom
NA	4599	hypothetical protein X975 05218 partial	custom
NA	4604	Phosphomevalonate kinase partial	custom
NA	4606	hypothetical protein X975 21756 partial	custom
NA	4607	Chitinase domain-containing protein 1 partial	custom
NA	4608	putative tRNA threonylcarbamoyladenosine biosynthesis protein Osgepl1 partial	custom
NA	4609	Tetraspanin-11 partial	custom

Supplemental Table 3 – continued from previous page

Arthropod Core ID	Spider Core ID	Putative Ortholog Description	Target Database
NA	4614	RNA polymerase II-associated protein 3 partial	custom
89865	4616	Vacuolar protein sorting-associated protein 72-like protein partial	custom
89716	4617	ATP-dependent Clp protease ATP-binding subunit clpX-like mitochondrial partial	custom
NA	4620	Thioredoxin domain-containing protein 15 partial	custom
NA	4622	FERM and PDZ domain-containing protein 4 partial	custom
NA	4624	hypothetical protein X975 00426 partial	custom
NA	4625	Protein SHQ1-like protein partial	custom
90132	4626	hypothetical protein X975 14088 partial	custom
NA	4627	hypothetical protein X975 15044 partial	custom
89962	4631	DnaJ-like protein subfamily C member 21 partial	custom
NA	4633	hypothetical protein X975 26412 partial	custom
NA	4637	Peptide-N(4)-(N-acetyl-beta-glucosaminyl)asparagine amidase partial	custom
NA	4638	Neurochondrin-like protein partial	custom
NA	4641	Tetratricopeptide repeat protein 17 partial	custom
NA	4648	hypothetical protein X975 18490 partial	custom
NA	4653	F-box/LRR-repeat protein 14 partial	custom
NA	4657	BRISC and BRCA1-A complex member 1 partial	custom
NA	4659	Ankyrin repeat and SOCS box protein 3 partial	custom

Supplemental Table 3 – continued from previous page

Arthropod Core ID	Spider Core ID	Putative Ortholog Description	Target Database
NA	4660	BolA-like protein 1 partial	custom
89963	4661	KRR1 small subunit processome component-like protein partial	custom
NA	4662	Mediator of RNA polymerase II transcription subunit 26 partial	custom
NA	4663	AN1-type zinc finger protein 6 partial	custom
NA	4665	Midnolin partial	custom
NA	4666	Type II inositol-145-trisphosphate 5-phosphatase partial	custom
NA	4668	hypothetical protein X975 26482 partial	custom
89826	4670	Phospholipid scramblase 2 partial	custom
89932	4671	Polyphosphoinositide phosphatase partial	custom
NA	4672	Gephyrin partial	custom
NA	4673	hypothetical protein X975 14777 partial	custom
90565	4674	Autophagy protein 5 partial	custom
NA	4675	Zinc transporter ZIP3 partial	custom
NA	4677	Basement membrane-specific heparan sulfate proteoglycan core protein partial	custom
NA	4679	Ras-related protein Rab-1A partial	custom
NA	4680	Proteasome activator complex subunit 3 partial	custom
NA	4682	Histidine triad nucleotide-binding protein 1 partial	custom
NA	4683	Protein LZIC partial	custom
NA	4684	PREDICTED: uncharacterized protein C7orf50 homolog isoform	nr
NA	4685	hypothetical protein X975 01775 partial	custom

Supplemental Table 3 – continued from previous page

Arthropod Core ID	Spider Core ID	Putative Ortholog Description	Target Database
NA	4686	Transposable element Tcb1 transposase partial	custom
NA	4687	Histone H2B partial	custom
NA	4688	hypothetical protein X975 06560 partial	custom
NA	4691	WD repeat-containing protein 46 partial	custom
89859	4693	WD repeat-containing protein 18 partial	custom
NA	4694	Diphthine synthase partial	custom
NA	4695	Anaphase-promoting complex subunit 7 partial	custom
NA	4696	hypothetical protein X975 09931 partial	custom
NA	4698	hypothetical protein X975 04396 partial	custom
90295	4699	Methyltransferase-like protein 13 partial	custom
90002	4700	Peptidylprolyl isomerase domain and WD repeat-containing protein 1 partial	custom
NA	4701	PREDICTED: nucleolar protein 9-like	nr
90398	4703	Elongation factor 1-beta partial	custom
NA	4705	putative actin-related protein 2/3 complex subunit 2 partial	custom
NA	4706	mRNA cap guanine-N7 methyltransferase partial	custom
NA	4707	CCR4-NOT transcription complex subunit 3 partial	custom
NA	4708	Ras guanine nucleotide exchange factor F partial	custom
NA	4709	GDP-L-fucose synthase partial	custom

Supplemental Table 3 – continued from previous page

Arthropod Core ID	Spider Core ID	Putative Ortholog Description	Target Database
NA	4710	39S ribosomal protein L27 mitochondrial partial	custom
NA	4711	MHC class II regulatory factor RFX1 partial	custom
90255	4712	putative ATP-dependent RNA helicase DDX10 partial	custom
NA	4714	60S ribosomal protein L7a partial	custom
NA	4715	Mitochondrial GTPase 1 partial	custom
NA	4718	Protein FAM8A1 partial	custom
NA	4719	hypothetical protein X975 16784 partial	custom
NA	4721	CUB and sushi domain-containing protein 3 partial	custom
NA	4722	Serine/threonine-protein kinase SMG1 partial	custom
NA	4723	PITH domain-containing protein partial	custom
NA	4724	calcium-binding protein partial	custom
NA	4727	hypothetical protein X975 23666 partial	custom
NA	4728	Pallidin partial	custom
89838	4729	Cell division cycle 7-related protein kinase partial	custom
NA	4730	Protein downstream neighbor of son-like protein partial	custom
NA	4731	Peptidyl-prolyl cis-trans isomerase H partial	custom
NA	4732	Pentatricopeptide repeat-containing protein 1 partial	custom
NA	4733	hypothetical protein X975 23126 partial	custom
NA	4734	Actin-related protein 10 partial	custom

Supplemental Table 3 – continued from previous page

Arthropod Core ID	Spider Core ID	Putative Ortholog Description	Target Database
NA	4735	RING finger protein 121 partial	custom
NA	4739	Pre-mRNA-splicing factor ATP-dependent RNA helicase PRP16 partial	custom
NA	4742	Complement component 1 Q subcomponent-binding protein mitochondrial partial	custom
NA	4744	PREDICTED: prefoldin subunit 2	nr
90459	4745	hypothetical protein X975 21674 partial	custom
NA	4746	Brefeldin A-inhibited guanine nucleotide-exchange protein 2 partial	custom
NA	4747	SURP and G-patch domain-containing protein 1 partial	custom
NA	4748	E3 ubiquitin-protein ligase RNF146 partial	custom
NA	4749	Pre-mRNA 3'-end-processing factor FIP1 partial	custom
89784	4751	Ras suppressor protein 1 partial	custom
NA	4754	hypothetical protein X975 01094 partial	custom
NA	4757	Serpin B8 partial	custom
NA	4759	LON peptidase N-terminal domain and RING finger protein 3 partial	custom
NA	4763	Kelch domain-containing protein 10 partial	custom
NA	4764	NA	nr
NA	4769	Serine/threonine-protein phosphatase 2A regulatory subunit B" subunit gamma partial	custom
89666	4779	Proteasome subunit beta type-3 partial	custom

Supplemental Table 3 – continued from previous page

Arthropod Core ID	Spider Core ID	Putative Ortholog Description	Target Database
NA	4780	Proteasome inhibitor PI31 subunit partial	custom
NA	4781	Acetylcholinesterase partial	custom
NA	4786	Hyccin partial	custom
90097	4787	Elongation factor Tu mitochondrial partial	custom
NA	4791	putative ATP-dependent RNA helicase DDX49 partial	custom
NA	4794	Proteasome subunit beta type-5 partial	custom
NA	4795	Elongation factor Tu GTP-binding domain-containing protein 1 partial	custom
NA	4796	Aquaporin-9 partial	custom
NA	4797	hypothetical protein BRAFLDRAFT 260972	nr
NA	4800	G patch domain-containing protein 1 partial	custom
NA	4801	Serine/threonine-protein kinase B-raf partial	custom
NA	4802	Rab-like protein 3 partial	custom
NA	4804	Lysophospholipid acyltransferase 5 partial	custom
89928	4806	DnaJ-like protein subfamily C member 17 partial	custom
NA	4809	Protein FAM45A partial	custom
NA	4810	BTB/POZ domain-containing protein 2 partial	custom
NA	4812	Ankyrin repeat and IBR domain-containing protein 1 partial	custom
NA	4815	Sialin partial	custom
NA	4823	PREDICTED: uncharacterized protein LOC106475977	nr

Supplemental Table 3 – continued from previous page

Arthropod Core ID	Spider Core ID	Putative Ortholog Description	Target Database
NA	4824	Exostosin-2 partial	custom
90217	4830	V-type proton ATPase subunit G partial	custom
NA	4832	hypothetical protein X975 21262 partial	custom
NA	4834	39S ribosomal protein L46 mitochondrial partial	custom
NA	4835	N6-adenosine-methyltransferase subunit partial	custom
NA	4842	Protein SMG8 partial	custom
NA	4843	putative cytosolic iron-sulfur protein assembly protein CIAO1 partial	custom
NA	4844	AP-4 complex subunit mu-1 partial	custom
NA	4846	Exosome complex component RRP43 partial	custom
NA	4847	Coatomer subunit zeta-1 partial	custom
NA	4853	Apoptosis regulator BAX partial	custom
NA	4865	Lariat debranching enzyme partial	custom
NA	4866	PAB-dependent poly(A)-specific ribonuclease subunit 2 partial	custom
90185	4869	TFIIH basal transcription factor complex helicase XPD subunit partial	custom
NA	4876	Translocase of inner mitochondrial membrane domain-containing protein 1 partial	custom
NA	4879	F-box only protein 25 partial	custom
90193	4882	Ubiquitin-conjugating enzyme E2 partial	custom
NA	4884	Protein FAM195A partial	custom

Supplemental Table 3 – continued from previous page

Arthropod Core ID	Spider Core ID	Putative Ortholog Description	Target Database
90650	4888	DNA-directed RNA polymerase III subunit RPC2 partial	custom
90412	4891	Malate dehydrogenase mitochondrial partial	custom
NA	4895	putative oxidoreductase partial	custom
89814	4897	Transmembrane emp24 domain-containing protein 7 partial	custom
89799	4898	Phosphatidylethanolamine-binding protein 1 partial	custom
NA	4899	Phenazine biosynthesis-like domain-containing protein 1 partial	custom
NA	4900	PREDICTED: citrate lyase subunit beta-like protein, mitochondrial	nr
NA	4902	Ribosome biogenesis regulatory protein-like protein partial	custom
NA	4903	hypothetical protein X975 07982 partial	custom
NA	4904	PREDICTED: multiple myeloma tumor-associated protein 2 homolog	nr
NA	4905	putative ATP-dependent RNA helicase DDX5 partial	custom
NA	4906	39S ribosomal protein L54 mitochondrial partial	custom
90257	4908	ATP synthase subunit d mitochondrial partial	custom
90604	4909	hypothetical protein X975 22133 partial	custom
NA	4910	Polymerase delta-interacting protein 2 partial	custom

Supplemental Table 3 – continued from previous page

Arthropod Core ID	Spider Core ID	Putative Ortholog Description	Target Database
NA	4911	39S ribosomal protein L49 mitochondrial partial	custom
89950	4912	60S ribosomal protein L28 partial	custom
90098	4913	PREDICTED: ATP synthase subunit g, mitochondrial-like	nr
NA	4914	N-acetylserotonin O-methyltransferase-like protein partial	custom
NA	4915	Transmembrane protein 59-like protein partial	custom
90244	4916	Translocon-associated protein subunit beta partial	custom
NA	4917	NADH dehydrogenase [ubiquinone] 1 beta subcomplex subunit 8 mitochondrial partial	custom
NA	4918	Vesicle-associated membrane protein-associated protein A partial	custom
89931	4919	hypothetical protein X975 09949 partial	custom
NA	4922	Beclin-1 partial	custom
90612	4923	Nicotinate phosphoribosyltransferase partial	custom
89926	4924	Mitochondrial import inner membrane translocase subunit tim16-A partial	custom
NA	4925	Breast carcinoma-amplified sequence 3-like protein partial	custom
NA	4929	Protein boule partial	custom
NA	4930	YEATS domain-containing protein 2 partial	custom
NA	4931	PREDICTED: tRNA (adenine(58)-N(1))-methyltransferase non-catalytic subunit TRM6-like	nr

Supplemental Table 3 – continued from previous page

Arthropod Core ID	Spider Core ID	Putative Ortholog Description	Target Database
NA	4932	hypothetical protein X975 16313 partial	custom
NA	4933	Limkain-b1 partial	custom
NA	4934	Fukutin partial	custom
90462	4935	Repressor of RNA polymerase III transcription MAF1-like protein partial	custom
NA	4936	DnaJ-like protein subfamily C member 8 partial	custom
90029	4937	60S ribosomal protein L13a partial	custom
NA	4938	putative peptidyl-tRNA hydrolase 2 partial	custom
NA	4939	Huntingtin-interacting protein K partial	custom
90613	4940	Histone-lysine N-methyltransferase SETMAR partial	custom
NA	4942	Lysozyme partial	custom
89854	4943	60S ribosomal protein L32 partial	custom
NA	4944	40S ribosomal protein SA partial	custom
NA	4945	Pre-mRNA branch site p14-like protein partial	custom
89668	4947	U3 small nucleolar ribonucleoprotein IMP3 partial	custom
NA	4949	Ras-related protein Rab-4B partial	custom
NA	4950	Synaptotagmin-9 partial	custom
90492	4951	60S ribosomal protein L10 partial	custom
NA	4953	Proteasome subunit alpha type-6 partial	custom
NA	4954	39S ribosomal protein L47 mitochondrial partial	custom
NA	4955	General transcription factor IIF subunit 2 partial	custom

Supplemental Table 3 – continued from previous page

Arthropod Core ID	Spider Core ID	Putative Ortholog Description	Target Database
NA	4957	Mitochondrial import inner membrane translocase subunit Tim22 partial	custom
NA	4958	UDP-N-acetylglucosamine transferase subunit ALG13-like protein partial	custom
NA	4959	40S ribosomal protein S25 partial	custom
NA	4960	U6 snRNA-associated Sm-like protein LSm4 partial	custom
NA	4961	Sorting nexin-12 partial	custom
NA	4962	CD2 antigen cytoplasmic tail-binding protein 2 partial	custom
89719	4963	PREDICTED: NADH dehydrogenase [ubiquinone] 1 alpha subcomplex subunit 13-like	nr
NA	4964	RNA-binding protein 4.1 partial	custom
NA	4965	MOB-like protein phocein partial	custom
90075	4966	RING-box protein 1 partial	custom
NA	4969	Cytochrome c oxidase subunit 4 isoform 2 mitochondrial partial	custom
NA	4970	WW domain-binding protein 2 partial	custom
NA	4971	Histone deacetylase complex subunit SAP18 partial	custom
NA	4972	Ubiquitin carboxyl-terminal hydrolase isozyme L3 partial	custom
90446	4974	Protein MAK16-like protein partial	custom
NA	4975	Pancreatic alpha-amylase partial	custom
NA	4976	hypothetical protein X975 08942 partial	custom

Supplemental Table 3 – continued from previous page

Arthropod Core ID	Spider Core ID	Putative Ortholog Description	Target Database
89698	4978	NADH dehydrogenase [ubiquinone] flavoprotein 2 mitochondrial partial	custom
NA	4979	Elongation factor Ts mitochondrial partial	custom
NA	4980	C-factor partial	custom
NA	4981	Pituitary tumor-transforming 1 protein-interacting protein partial	custom
NA	4982	Sulfotransferase 1C2 partial	custom
89842	4983	PREDICTED: protein FRG1 homolog	nr
NA	4984	Myophilin partial	custom
NA	4985	F-actin-capping protein subunit beta partial	custom
NA	4986	Cofilin/actin-depolymerizing factor-like protein partial	custom
NA	4987	PREDICTED: cytochrome c oxidase subunit 4 isoform 1, mitochondrial-like isoform	nr
NA	4988	hypothetical protein X975 05975 partial	custom
NA	4989	Dihydropteridine reductase partial	custom
NA	4992	Pleckstrin domain-containing family F member 2 partial	custom
NA	4993	Peptidyl-prolyl cis-trans isomerase partial	custom
NA	4994	Serine/threonine-protein phosphatase 2A activator partial	custom
89740	4995	hypothetical protein X975 11002 partial	custom
NA	4997	3-hydroxyisobutyryl-CoA hydrolase mitochondrial partial	custom
NA	4998	Negative elongation factor A partial	custom

Supplemental Table 3 – continued from previous page

Arthropod Core ID	Spider Core ID	Putative Ortholog Description	Target Database
NA	4999	hypothetical protein X975 03197 partial	custom
90546	5001	Thioredoxin domain-containing protein 9 partial	custom
NA	5004	hypothetical protein X975 00239 partial	custom
NA	5005	hypothetical protein X975 06519 partial	custom
NA	5006	hypothetical protein X975 26509 partial	custom
NA	5008	ATP synthase subunit gamma mitochondrial partial	custom
NA	5010	Serine/threonine-protein phosphatase PGAM5 mitochondrial partial	custom
NA	5015	Prothoracicostatic peptide partial	custom
89930	5017	26S protease regulatory subunit 4 partial	custom
89650	5022	LETM1 and EF-hand domain-containing protein 1 mitochondrial partial	custom
NA	5024	Techylectin-5A partial	custom
NA	5025	hypothetical protein X975 14858 partial	custom
NA	5026	CCAAT/enhancer-binding protein alpha partial	custom
NA	5028	hypothetical protein X975 16815 partial	custom
NA	5030	RNA 3'-terminal phosphate cyclase partial	custom
NA	5039	DNA repair protein RAD50 partial	custom
NA	5041	Clotting factor B partial	custom
NA	5043	DNA polymerase epsilon subunit 3 partial	custom
90495	5048	tRNA (guanine(26)-N(2))-dimethyltransferase partial	custom

Supplemental Table 3 – continued from previous page

Arthropod Core ID	Spider Core ID	Putative Ortholog Description	Target Database
NA	5049	39S ribosomal protein L9 mitochondrial partial	custom
NA	5051	XK-related protein 6 partial	custom
NA	5052	Translational activator of cytochrome c oxidase 1 partial	custom
NA	5053	Cysteine protease ATG4B partial	custom
NA	5054	Endonuclease G mitochondrial partial	custom
NA	5057	Ubiquitin-conjugating enzyme E2 G1 partial	custom
NA	5059	Histone acetyltransferase KAT6B partial	custom
NA	5061	CCR4-NOT transcription complex subunit 3 partial	custom
NA	5062	Eukaryotic translation initiation factor 3 subunit H partial	custom
NA	5064	hypothetical protein X975 20518 partial	custom
NA	5065	hypothetical protein X975 12238 partial	custom
NA	5069	dCTP pyrophosphatase 1 partial	custom
90148	5070	L-xylulose reductase partial	custom
NA	5072	L-threonine 3-dehydrogenase partial	custom
NA	5074	Peroxisomal membrane protein PEX16 partial	custom
NA	5076	hypothetical protein X975 16156 partial	custom
90285	5077	V-type proton ATPase subunit D partial	custom
NA	5078	39S ribosomal protein L18 mitochondrial partial	custom
NA	5079	39S ribosomal protein L28 mitochondrial partial	custom

Supplemental Table 3 – continued from previous page

Arthropod Core ID	Spider Core ID	Putative Ortholog Description	Target Database
NA	5082	LIM domain-containing protein 1 partial	custom
NA	5085	Ankyrin repeat domain-containing protein 40 partial	custom
89673	5086	tRNA methyltransferase 112-like protein partial	custom
NA	5087	Nuclear transcription factor Y subunit gamma partial	custom
NA	5091	U11/U12 small nuclear ribonucleoprotein partial	custom
NA	5092	NADH dehydrogenase [ubiquinone] 1 beta subcomplex subunit 7 partial	custom
NA	5093	Akirin partial	custom
NA	5094	B-cell receptor-associated protein 31 partial	custom
90119	5096	Deoxyhypusine hydroxylase partial	custom
89790	5099	Transmembrane protein 104 partial	custom
NA	5103	RNA exonuclease 1-like protein partial	custom
NA	5107	Ninjurin-1 partial	custom
NA	5109	Alpha-parvin partial	custom
NA	5110	putative lysosomal cobalamin transporter partial	custom
NA	5111	Cell division cycle protein 123-like protein partial	custom
NA	5112	putative Dol-P-Man:Man(7)GlcNAc(2)-PP-Dol alpha-16-mannosyltransferase partial	custom
NA	5116	TATA box-binding protein-like protein 1 partial	custom
NA	5119	Cell growth-regulating nucleolar protein partial	custom

Supplemental Table 3 – continued from previous page

Arthropod Core ID	Spider Core ID	Putative Ortholog Description	Target Database
89914	5120	Enhancer of rudimentary-like protein partial	custom
NA	5121	hypothetical protein X975 23691 partial	custom
90128	5123	RING finger protein 113A partial	custom
NA	5124	putative NADH dehydrogenase [ubiquinone] 1 alpha subcomplex subunit 12 partial	custom
NA	5125	Nuclear nucleic acid-binding protein C1D partial	custom
NA	5127	E2F-associated phosphoprotein partial	custom
NA	5128	28S ribosomal protein S2 mitochondrial partial	custom
NA	5129	Proteasome subunit beta type-2 partial	custom
NA	5130	DCN1-like protein 3 partial	custom
NA	5133	TGF-beta-activated kinase 1 and MAP3K7-binding protein 3 partial	custom
90158	5137	Beta-lactamase-like protein 2 partial	custom
NA	5138	hypothetical protein X975 10248 partial	custom
NA	5140	Serine/arginine-rich splicing factor 12 partial	custom
NA	5141	Glucosidase 2 subunit beta partial	custom
NA	5142	Transcriptional adapter 2-alpha partial	custom
NA	5147	Dehydrogenase/reductase SDR family member 7 partial	custom
NA	5149	Pre-rRNA-processing protein TSR2-like protein partial	custom

Supplemental Table 3 – continued from previous page

Arthropod Core ID	Spider Core ID	Putative Ortholog Description	Target Database
NA	5152	Zinc finger protein 330-like protein partial	custom
NA	5154	RNA pseudouridylate synthase domain-containing protein 3 partial	custom
NA	5156	hypothetical protein X975 22124 partial	custom
NA	5157	Epsilon-sarcoglycan partial	custom
NA	5158	Cytochrome b-c1 complex subunit Rieske mitochondrial partial	custom
NA	5159	tRNA dimethylallyl-transferase mitochondrial partial	custom
NA	5161	7SK snRNA methylphosphate capping enzyme partial	custom
NA	5162	GA-binding protein subunit beta-1 partial	custom
NA	5163	putative proline–tRNA ligase mitochondrial partial	custom
NA	5166	putative ATP-dependent RNA helicase DDX31 partial	custom
NA	5167	PREDICTED: glutamyl-tRNA(Gln) amidotransferase subunit C, mitochondrial	nr
NA	5168	Protein ECT2 partial	custom
NA	5169	EP300-interacting inhibitor of differentiation 3 partial	custom
NA	5176	Zinc finger BED domain-containing protein 5 partial	custom
NA	5180	Ceramide synthase 6 partial	custom
NA	5181	H/ACA ribonucleoprotein complex non-core subunit NAF1 partial	custom

Supplemental Table 3 – continued from previous page

Arthropod Core ID	Spider Core ID	Putative Ortholog Description	Target Database
NA	5187	Adrenodoxin mitochondrial partial	custom
89972	5188	Ribosome biogenesis protein bop1 partial	custom
NA	5189	Peroxisome biogenesis factor 1 partial	custom
NA	5190	Trans-aconitate 2-methyltransferase partial	custom
NA	5191	Mitogen-activated protein kinase 1 partial	custom
NA	5192	Peptidyl-prolyl cis-trans isomerase FKBP2 partial	custom
90216	5193	Anaphase-promoting complex subunit 10 partial	custom
NA	5195	putative E3 ubiquitin-protein ligase MYCBP2 partial	custom
NA	5198	hypothetical protein X975 21083 partial	custom
NA	5199	Ras-related GTP-binding protein C partial	custom
NA	5200	Pleckstrin domain-containing family B member 2 partial	custom
NA	5206	PREDICTED: Bardet-Biedl syndrome 10 protein isoform	nr
NA	5207	hypothetical protein X975 25400 partial	custom
NA	5209	Geranylgeranyl transferase type-1 subunit beta partial	custom
NA	5210	tRNA wybutosine-synthesizing protein 3-like protein partial	custom
NA	5213	PREDICTED: CBF1-interacting corepressor, putative	nr
NA	5217	Fas-binding factor 1-like protein partial	custom
NA	5218	RPE-spondin partial	custom

Supplemental Table 3 – continued from previous page

Arthropod Core ID	Spider Core ID	Putative Ortholog Description	Target Database
NA	5219	Trimeric intracellular cation channel type B partial	custom
89839	5221	Glutamyl-tRNA(Gln) amidotransferase subunit A mitochondrial partial	custom
NA	5223	putative ribosomal RNA methyltransferase 1 partial	custom
NA	5224	Arginine/serine-rich protein PNISR partial	custom
NA	5227	Protein NipSnap-like protein 3A partial	custom
NA	5228	E3 ubiquitin-protein ligase RNF14 partial	custom
NA	5230	Dual specificity protein phosphatase 14 partial	custom
NA	5231	Programmed cell death protein 2 partial	custom
NA	5233	putative ankyrin repeat protein partial	custom
90086	5234	60S ribosomal protein L18a partial	custom
90336	5236	12-dihydroxy-3-keto-5-methylthiopentene dioxygenase partial	custom
NA	5238	Endoplasmic reticulum resident protein 29 partial	custom
90190	5240	Proteasome subunit alpha type-5 partial	custom
90483	5244	Protein pygopus partial	custom
NA	5247	5'-nucleotidase domain-containing protein 1	nr
90422	5248	Protein unc-119-like protein partial	custom
NA	5250	Transmembrane and coiled-coil domains protein 1 partial	custom
NA	5252	Zinc finger CCCH-type with G patch domain-containing protein partial	custom

Supplemental Table 3 – continued from previous page

Arthropod Core ID	Spider Core ID	Putative Ortholog Description	Target Database
NA	5253	Deubiquitinating protein partial	custom
NA	5255	Zinc finger protein 57 partial	custom
NA	5256	SET domain-containing protein 4 partial	custom
NA	5258	Porphobilinogen deaminase partial	custom
NA	5259	Alkaline ceramidase partial	custom
NA	5260	Protein YIPF5 partial	custom
NA	5262	Protein BUD31-like protein partial	custom
89680	5263	40S ribosomal protein S17 partial	custom
NA	5265	Ubiquitin-conjugating enzyme E2 G2 partial	custom
NA	5268	Ras-related protein Rab-32 partial	custom
89735	5269	hypothetical protein X975 26960 partial	custom
90004	5270	Enoyl-CoA hydratase mitochondrial partial	custom
NA	5271	RNA methyltransferase-like protein 1 partial	custom
NA	5273	Hypermethylated in cancer 2 protein partial	custom
90563	5274	N(6)-adenine-specific DNA methyltransferase 2 partial	custom
NA	5275	KDEL motif-containing protein 1 partial	custom
NA	5276	hypothetical protein X975 23653 partial	custom
89993	5277	Proteasome subunit alpha type-2 partial	custom
NA	5278	Adenylate kinase isoenzyme 1 partial	custom
NA	5279	26S protease regulatory subunit 10B partial	custom

Supplemental Table 3 – continued from previous page

Arthropod Core ID	Spider Core ID	Putative Ortholog Description	Target Database
NA	5280	SH2B adapter protein 2 partial	custom
NA	5282	Tubulin-specific chaperone A partial	custom
NA	5283	Mediator of RNA polymerase II transcription subunit 6 partial	custom
NA	5285	Trophoblast glycoprotein partial	custom
90043	5288	PREDICTED: WASH complex subunit CCDC53-like	nr
NA	5290	PHD finger protein 12 partial	custom
NA	5291	Protein NipSnap partial	custom
NA	5292	Rap1 GTPase-GDP dissociation stimulator 1 partial	custom
NA	5294	PREDICTED: dr1-associated corepressor-like	nr
NA	5301	Breast cancer anti-estrogen resistance protein 1 partial	custom
NA	5302	Centrosomal protein partial	custom
NA	5308	Tubulin beta-1 chain partial	custom
90238	5310	putative methyltransferase WBSCR22 partial	custom
NA	5315	Wiskott-Aldrich syndrome protein partial	custom
NA	5316	Neighbor of COX4 partial	custom
NA	5317	Transmembrane protein 209 partial	custom
NA	5320	E3 ubiquitin-protein ligase MARCH1 partial	custom
NA	5322	40S ribosomal protein S3-A partial	custom
89694	5323	Zinc finger protein ZPR1 partial	custom
NA	5324	Vacuolar-sorting protein SNF8 partial	custom

Supplemental Table 3 – continued from previous page

Arthropod Core ID	Spider Core ID	Putative Ortholog Description	Target Database
NA	5327	Bromodomain-containing protein 7 partial	custom
NA	5332	Mediator of RNA polymerase II transcription subunit 10 partial	custom
NA	5333	Zinc finger protein 252 partial	custom
NA	5335	MAGUK p55 subfamily member 5 partial	custom
NA	5338	CTD small phosphatase-like protein partial	custom
NA	5343	Serine/threonine-protein kinase ULK3 partial	custom
NA	5346	Transmembrane protein adipocyte-associated 1-like protein partial	custom
89689	5347	RNA and export factor-binding protein 2 partial	custom
NA	5349	Transmembrane emp24 domain-containing protein 1 partial	custom
NA	5352	D-aspartate oxidase partial	custom
NA	5354	Transmembrane protein partial	custom
NA	5356	Ectopic P granules protein 5-like protein partial	custom
NA	5359	Mannose-6-phosphate isomerase partial	custom
NA	5360	PP2C-like domain-containing protein partial	custom
NA	5361	GTP-binding protein 128up partial	custom
NA	5365	Glycogenin-1 partial	custom
NA	5366	hypothetical protein X975 05332 partial	custom

Supplemental Table 3 – continued from previous page

Arthropod Core ID	Spider Core ID	Putative Ortholog Description	Target Database
NA	5367	hypothetical protein X975 06366 partial	custom
NA	5374	Charged multivesicular body protein 7 partial	custom
NA	5375	Synaptobrevin partial	custom
NA	5376	Autophagy-related protein 9A partial	custom
NA	5378	Charged multivesicular body protein 5 partial	custom
NA	5380	RING finger protein 17 partial	custom
NA	5381	Protein MEMO1 partial	custom
89944	5382	Inositol oxygenase partial	custom
NA	5384	Kelch-like protein 18 partial	custom
NA	5387	MKL/myocardin-like protein 2 partial	custom
NA	5396	hypothetical protein X975 04874 partial	custom
89965	5397	40S ribosomal protein S9 partial	custom
NA	5400	ATP synthase subunit delta mitochondrial partial	custom
NA	5402	Tubulin-specific chaperone E partial	custom
NA	5407	Nicotinate-nucleotide pyrophosphorylase [carboxylating] partial	custom
NA	5408	Ribosomal RNA small subunit methyltransferase NEP1 partial	custom
NA	5409	Tetratricopeptide repeat protein 1 partial	custom
NA	5410	PREDICTED: tubulin polyglutamylase complex subunit-2like isoform	nr
NA	5411	CCR4-NOT transcription complex subunit 10 partial	custom

Supplemental Table 3 – continued from previous page

Arthropod Core ID	Spider Core ID	Putative Ortholog Description	Target Database
NA	5412	Histone-lysine N-methyltransferase setd3 partial	custom
NA	5413	COMM domain-containing protein 2 partial	custom
NA	5417	Transmembrane emp24 domain-containing protein 6 partial	custom
NA	5418	Protein FAM149B1 partial	custom
NA	5419	Methionine aminopeptidase 1 partial	custom
NA	5423	Transmembrane protein 41B partial	custom
NA	5424	Tetraspanin-7 partial	custom
89920	5426	Adenosylhomocysteinase A partial	custom
NA	5430	UNC93-like protein MFSD11 partial	custom
90550	5453	Ubiquitin-conjugating enzyme E2 variant 2 partial	custom
89836	5459	Alpha-methylacyl-CoA racemase partial	custom
NA	5460	Noelin partial	custom
NA	5461	Peptidyl-prolyl cis-trans isomerase H partial	custom
NA	5463	hypothetical protein X975 12721 partial	custom
NA	5465	SH3 domain-containing kinase-binding protein 1 partial	custom
NA	5468	PAX-interacting protein 1 partial	custom
89819	5476	Selenocysteine methyltransferase partial	custom
NA	5477	Importin subunit alpha-2 partial	custom
NA	5478	U11/U12 small nuclear ribonucleoprotein partial	custom

Supplemental Table 3 – continued from previous page

Arthropod Core ID	Spider Core ID	Putative Ortholog Description	Target Database
NA	5484	U3 small nucleolar RNA-associated protein 6-like protein partial	custom
NA	5486	Methyltransferase-like protein 10 partial	custom
NA	5487	Coiled-coil domain-containing protein 115 partial	custom
NA	5489	Neuralized-like protein 2 partial	custom
NA	5492	Lamin Dm0 partial	custom
NA	5494	PREDICTED: DNA polymerase epsilon subunit 4-like	nr
NA	5495	WD and tetratricopeptide repeats protein 1 partial	custom
NA	5496	PC4 and SFRS1-interacting protein partial	custom
NA	5497	Aprataxin and PNK-like factor partial	custom
90269	5499	hypothetical protein X975 22271 partial	custom
NA	5500	Inhibitor of growth protein 1 partial	custom
NA	5501	hypothetical protein X975 21918 partial	custom
90183	5503	40S ribosomal protein S7 partial	custom
90496	5504	hypothetical protein X975 15266 partial	custom
NA	5506	Glutathione S-transferase 1 isoform C partial	custom
NA	5507	Breast cancer metastasis-suppressor 1-like protein-A partial	custom
NA	5508	CDGSH iron-sulfur domain-containing protein 3 mitochondrial partial	custom
NA	5509	E3 ubiquitin-protein ligase RING2-A partial	custom

Supplemental Table 3 – continued from previous page

Arthropod Core ID	Spider Core ID	Putative Ortholog Description	Target Database
NA	5510	Methionine aminopeptidase 1D mitochondrial partial	custom
NA	5511	Protein FAM32A partial	custom
NA	5512	DNA-directed RNA polymerase III subunit RPC9 partial	custom
90220	5514	hypothetical protein X975 14274 partial	custom
NA	5515	putative ATP-dependent Clp protease proteolytic subunit mitochondrial partial	custom
89982	5516	Troponin C partial	custom
90631	5517	ribosomal protein S26, putative	nr
NA	5518	Calmodulin partial	custom
NA	5519	Monoacylglycerol lipase ABHD12 partial	custom
NA	5520	Tetraspanin-31 partial	custom
NA	5523	Rho guanine nucleotide exchange factor 7 partial	custom
NA	5524	Protein deltex-2 partial	custom
NA	5525	Proteasome assembly chaperone 4	nr
NA	5526	putative proline dehydrogenase 2 partial	custom
NA	5527	DCC-interacting protein 13-alpha partial	custom
NA	5529	Arginine and glutamate-rich protein 1-A partial	custom
NA	5530	Sodium/potassium/calcium exchanger 5 partial	custom
NA	5531	3'-5' exoribonuclease 1 partial	custom
NA	5532	WD repeat-containing protein 89 partial	custom
89810	5536	Sister chromatid cohesion protein DCC1 partial	custom

Supplemental Table 3 – continued from previous page

Arthropod Core ID	Spider Core ID	Putative Ortholog Description	Target Database
NA	5537	hypothetical protein X975 06272 partial	custom
NA	5538	Transforming protein v-Fos/v-Fox partial	custom
NA	5539	Protein dopey-1 partial	custom
NA	5540	GDP-Man:Man(3)GlcNAc(2)-PP-Dol alpha-12-mannosyltransferase partial	custom
NA	5543	hypothetical protein X975 16083 partial	custom
89709	5545	Inosine triphosphate pyrophosphatase partial	custom
90284	5547	40S ribosomal protein S18 partial	custom
NA	5552	Protein lin-52-like protein partial	custom
NA	5553	Acid sphingomyelinase-like phosphodiesterase 3b partial	custom
NA	5554	Ubiquitin carboxyl-terminal hydrolase 40 partial	custom
89705	5555	Replication factor C subunit 4 partial	custom
NA	5557	Muscle-specific protein 20 partial	custom
NA	5558	Mediator of RNA polymerase II transcription subunit 8 partial	custom
90039	5559	60S ribosomal protein L17 partial	custom
NA	5560	Myosin regulatory light polypeptide 9 partial	custom
NA	5562	Leucine-rich repeat-containing protein 20 partial	custom
NA	5563	Protein canopy-like protein partial	custom
NA	5564	DNA-directed RNA polymerase II subunit RPB11 partial	custom

Supplemental Table 3 – continued from previous page

Arthropod Core ID	Spider Core ID	Putative Ortholog Description	Target Database
NA	5565	Synaptic vesicle membrane protein VAT-1-like protein partial	custom
NA	5566	Zinc finger protein DPF3 partial	custom
NA	5568	Phospholipase A2 partial	custom
NA	5569	Baculoviral IAP repeat-containing protein 7-A partial	custom
90630	5570	Glutaredoxin-related protein 5 mitochondrial partial	custom
NA	5572	Transcription factor AP-1 partial	custom
NA	5573	hypothetical protein X975 05022 partial	custom
NA	5574	Krueppel-like factor 5 partial	custom
NA	5577	hypothetical protein X975 21174 partial	custom
NA	5578	NA	nr
NA	5579	U24-ctenitoxin-Pn1a partial	custom
NA	5581	40S ribosomal protein S4 partial	custom
NA	5582	Cysteine dioxygenase type 1 partial	custom
NA	5584	Tumor protein D54 partial	custom
NA	5585	hypothetical protein X975 19303 partial	custom
NA	5586	Transcription factor HES-4-A partial	custom
NA	5587	Ras-related protein Rab-6A partial	custom
NA	5589	Tyrosine-protein phosphatase Lar partial	custom
NA	5590	Troponin C partial	custom
90064	5591	Troponin T partial	custom
NA	5593	Deoxyuridine 5'-triphosphate nucleotidohydrolase mitochondrial partial	custom

Supplemental Table 3 – continued from previous page

Arthropod Core ID	Spider Core ID	Putative Ortholog Description	Target Database
NA	5595	H/ACA ribonucleo-protein complex sub-unit 2-like protein partial	custom
NA	5596	Electron transfer flavoprotein subunit alpha mitochondrial partial	custom
NA	5597	putative Bax inhibitor 1 partial	custom
NA	5599	Structural mainte-nance of chromo-somes protein 2 partial	custom
NA	5600	hypothetical protein X975 22155 partial	custom
NA	5601	THO complex sub-unit 6-like protein partial	custom
89682	5602	Alanine aminotrans-ferase 2 partial	custom
NA	5603	Vacuolar protein sorting-associated protein VTA1-like protein partial	custom
89743	5605	NADH dehydroge-nase [ubiquinone] 1 beta subcomplex sub-unit 5 mitochondrial partial	custom
90149	5607	Peptidyl-prolyl cis-trans isomerase FKBP4 partial	custom
NA	5610	Peritrophin-1 partial	custom
NA	5611	Multiple coagulation factor deficiency pro-tein 2-like protein par-tial	custom
NA	5612	Tyrosine kinase re-ceptor Cad96Ca par-tial	custom
NA	5614	Syntenin-1 partial	custom
90100	5615	Histone H1 partial	custom
NA	5616	Serine/threonine-protein kinase H1-like protein partial	custom

Supplemental Table 3 – continued from previous page

Arthropod Core ID	Spider Core ID	Putative Ortholog Description	Target Database
NA	5619	FGFR1 oncogene partner 2-like protein partial	custom
NA	5620	Replication factor C subunit 2 partial	custom
90473	5629	Nuclease-sensitive element-binding protein 1 partial	custom
NA	5631	TBC1 domain family member 15 partial	custom
NA	5632	hypothetical protein X975 07405 partial	custom
NA	5634	hypothetical protein X975 11098 partial	custom
NA	5636	Histone acetyltransferase KAT6B partial	custom
90651	5637	Mechanosensory protein 2 partial	custom
NA	5641	Histone-lysine N-methyltransferase MLL3 partial	custom
89722	5642	60S ribosomal protein L26 partial	custom
NA	5643	WD repeat-containing protein 3 partial	custom
NA	5644	BRCA1-A complex subunit BRE partial	custom
NA	5648	Ufm1-specific protease 2 partial	custom
NA	5649	G patch domain-containing protein 3 partial	custom
NA	5651	Transmembrane protein 47 partial	custom
NA	5653	Cuticle protein 10.9 partial	custom
NA	5654	CCR4-NOT transcription complex subunit 3 partial	custom
NA	5658	E3 ubiquitin-protein ligase listerin partial	custom
NA	5659	NAD-dependent deacetylase sirtuin-7 partial	custom
NA	5660	39S ribosomal protein L17 mitochondrial partial	custom

Supplemental Table 3 – continued from previous page

Arthropod Core ID	Spider Core ID	Putative Ortholog Description	Target Database
NA	5661	60S ribosomal protein L11 partial	custom
NA	5664	Ubiquitin-conjugating enzyme E2 S partial	custom
NA	5667	STE20-related kinase adapter protein alpha partial	custom
NA	5668	Coronin-6 partial	custom
NA	5671	Phosphatidylinositol-4-phosphate 3-kinase C2 domain-containing subunit alpha partial	custom
NA	5674	RNA polymerase II subunit A C-terminal domain phosphatase SSU72 partial	custom
NA	5675	Ribosome production factor 2-like protein partial	custom
NA	5676	Activating signal cointegrator 1 complex subunit 1 partial	custom
89678	5677	STIP1 and U box-containing protein 1 partial	custom
NA	5680	hypothetical protein X975 02230 partial	custom
NA	5681	Eukaryotic translation initiation factor 4E type 2 partial	custom
NA	5683	WD repeat-containing protein 24 partial	custom
NA	5685	Cysteine and histidine-rich protein 1-B partial	custom
NA	5686	Calcium-independent phospholipase A2-gamma partial	custom
NA	5687	Cirhin-like	nr
NA	5688	Synaptobrevin-like protein YKT6 partial	custom
NA	5689	hypothetical protein X975 17624 partial	custom

Supplemental Table 3 – continued from previous page

Arthropod Core ID	Spider Core ID	Putative Ortholog Description	Target Database
NA	5690	Ubiquitin-conjugating enzyme E2 J2 partial	custom
NA	5694	NADH dehydrogenase [ubiquinone] 1 alpha subcomplex subunit 11 partial	custom
NA	5696	Kanadaptin partial	custom
NA	5698	Hematopoietic prostaglandin D synthase partial	custom
NA	5701	Protein pelota partial	custom
NA	5704	60S ribosomal protein L27 partial	custom
NA	5705	Integrator complex subunit 10 partial	custom
NA	5715	Adrenodoxin mitochondrial partial	custom
89825	5716	Ribosome biogenesis protein NSA2-like protein partial	custom
NA	5717	tRNA wybutosine-synthesizing protein 5 partial	custom
NA	5718	Regucalcin partial	custom
NA	5719	Transmembrane emp24 domain-containing protein eca partial	custom
90276	5720	NADH dehydrogenase [ubiquinone] 1 beta subcomplex subunit 10 partial	custom
89981	5721	U6 snRNA-associated Sm-like protein LSm1 partial	custom
NA	5724	39S ribosomal protein L51 mitochondrial partial	custom
NA	5725	Fanconi anemia-associated protein partial	custom
89649	5726	hypothetical protein X975 20170 partial	custom
NA	5727	hypothetical protein X975 00999 partial	custom

Supplemental Table 3 – continued from previous page

Arthropod Core ID	Spider Core ID	Putative Ortholog Description	Target Database
NA	5728	Iron-sulfur cluster assembly 1-like protein mitochondrial partial	custom
NA	5729	hypothetical protein X975 19674 partial	custom
NA	5732	Alpha-13/16-mannosyltransferase ALG2 partial	custom
NA	5735	hypothetical protein X975 13488 partial	custom
NA	5737	Cytoplasmic tRNA 2-thiolation protein 1 partial	custom
NA	5740	putative proline racemase partial	custom
NA	5741	Omega-amidase NIT2 partial	custom
NA	5742	hypothetical protein X975 10040 partial	custom
NA	5744	UMP-CMP kinase partial	custom
NA	5745	Spartin partial	custom
NA	5746	Oxidoreductase NAD-binding domain-containing protein 1 partial	custom
NA	5747	RNA 3'-terminal phosphate cyclase-like protein partial	custom
NA	5749	Box C/D snoRNA protein 1 partial	custom
90241	5750	ATP-dependent RNA helicase DDX55 partial	custom
NA	5752	PDZ and LIM domain protein Zasp partial	custom
NA	5754	Integrin-alpha FG-GAP repeat-containing protein 2 partial	custom
NA	5755	hypothetical protein X975 11890 partial	custom
NA	5759	hypothetical protein X975 23326 partial	custom
NA	5760	hypothetical protein X975 12087 partial	custom

Supplemental Table 3 – continued from previous page

Arthropod Core ID	Spider Core ID	Putative Ortholog Description	Target Database
NA	5761	Heterogeneous nuclear ribonucleoprotein U-like protein 1 partial	custom
NA	5762	hypothetical protein X975 25792 partial	custom
NA	5763	Phosphoacetylglucosamine mutase partial	custom
NA	5764	hypothetical protein X975 05751 partial	custom
NA	5765	hypothetical protein X975 09706 partial	custom
NA	5766	BCL-6 corepressor partial	custom
NA	5770	Thioredoxin reductase 1 cytoplasmic partial	custom
NA	5771	Programmed cell death protein 6 partial	custom
NA	5774	Fatty acid synthase partial	custom
NA	5776	putative tRNA pseudouridine synthase Pus10 partial	custom
NA	5781	PREDICTED: golgin subfamily A member 7-like	nr
NA	5782	putative RNA polymerase II subunit B1 CTD phosphatase rpap2 partial	custom
NA	5783	hypothetical protein X975 18250 partial	custom
NA	5786	Transmembrane protein 242 partial	custom
NA	5787	Integrator complex subunit 8 partial	custom
NA	5788	Sentrin-specific protease 7 partial	custom
NA	5795	Cell cycle checkpoint control protein RAD9A partial	custom
NA	5796	RILP-like protein-like protein partial	custom
NA	5799	Ribosomal RNA processing protein 36-like protein partial	custom

Supplemental Table 3 – continued from previous page

Arthropod Core ID	Spider Core ID	Putative Ortholog Description	Target Database
NA	5805	hypothetical protein X975 05627 partial	custom
NA	5810	Neuroblastoma-amplified sequence partial	custom
NA	5813	Nischarin partial	custom
NA	5814	hypothetical protein X975 18101 partial	custom
NA	5815	ADP-ribosylation factor-like protein 1 partial	custom
NA	5817	Protein FAM210A partial	custom
NA	5818	putative family 31 glucosidase partial	custom
90266	5819	Receptor expression-enhancing protein 5 partial	custom
NA	5822	Tetratricopeptide repeat protein 32 partial	custom
NA	5823	hypothetical protein X975 10817 partial	custom
NA	5824	Sphingomyelin phosphodiesterase D SpeSicTox-betaIB2a partial	custom
NA	5827	Protein FAM43A partial	custom
NA	5828	Exosome complex component RRP46 partial	custom
NA	5829	39S ribosomal protein L42 mitochondrial partial	custom
NA	5830	Spermatogenesis-defective protein 39-like protein partial	custom
NA	5831	Signal recognition particle protein partial	custom
90089	5833	ATP synthase mitochondrial F1 complex assembly factor 2 partial	custom
NA	5834	hypothetical protein X975 19386 partial	custom
NA	5836	Multifunctional protein ADE2 partial	custom

Supplemental Table 3 – continued from previous page

Arthropod Core ID	Spider Core ID	Putative Ortholog Description	Target Database
90654	5839	Pyrroline-5-carboxylate reductase 2 partial	custom
NA	5841	hypothetical protein X975 26581 partial	custom
NA	5845	Mitochondrial fission factor partial	custom
NA	5846	hypothetical protein X975 12332 partial	custom
NA	5847	Protein FAM50-like protein partial	custom
NA	5848	PREDICTED: enkurin domain-containing protein 1-like	nr
NA	5849	Enoyl-CoA delta isomerase 1 mitochondrial partial	custom
NA	5850	Nuclear pore complex protein Nup88 partial	custom
NA	5851	Ubiquitin carboxyl-terminal hydrolase 22-A partial	custom
NA	5852	Succinyl-CoA ligase [GDP-forming] subunit beta mitochondrial partial	custom
NA	5854	MIT domain-containing protein 1 partial	custom
NA	5855	Tubulin-specific chaperone A partial	custom
NA	5856	Hexosaminidase D partial	custom
NA	5857	Peptidoglycan-recognition protein SC2 partial	custom
NA	5859	Slowpoke-binding protein partial	custom
NA	5860	hypothetical protein X975 04608 partial	custom
90591	5861	tRNA-dihydrouridine(16/17) synthase [NAD(P)(+)]-like protein partial	custom

Supplemental Table 3 – continued from previous page

Arthropod Core ID	Spider Core ID	Putative Ortholog Description	Target Database
89787	5862	Protein transport protein Sec61 subunit alpha isoform 2 partial	custom
NA	5864	Methenyltetrahydrofolate synthase domain-containing protein partial	custom
89715	5865	Translation factor GUF1-like protein mitochondrial partial	custom
NA	5866	tRNA (guanine(10)-N2)-methyltransferase-like protein partial	custom
NA	5868	J domain-containing protein partial	custom
NA	5869	PREDICTED: general transcription factor 3C polypeptide 2-like	nr
NA	5870	Docking protein 6 partial	custom
89974	5871	CTD nuclear envelope phosphatase 1A partial	custom
NA	5872	Thioredoxin domain-containing protein 17 partial	custom
NA	5876	PREDICTED: PRKR-interacting protain 1 homolog	nr
NA	5878	Isocitrate dehydrogenase [NAD] subunit gamma 1 mitochondrial partial	custom
NA	5879	E3 ubiquitin-protein ligase HUWE1 partial	custom
NA	5880	LysM and hypothetical peptidoglycan-binding domain-containing protein 2 partial	custom
NA	5881	Multidrug resistance protein 1B partial	custom
NA	5885	Intraflagellar transport protein 20-like protein partial	custom

Supplemental Table 3 – continued from previous page

Arthropod Core ID	Spider Core ID	Putative Ortholog Description	Target Database
90355	5887	Beta-soluble NSF attachment protein partial	custom
NA	5890	Phosphopantothenoylcysteine decarboxylase partial	custom
NA	5891	PREDICTED: anaphase-promoting complex subunit 15-like	nr
NA	5893	COMM domain-containing protein 3 partial	custom
NA	5894	HEAT repeat-containing protein 6 partial	custom
89818	5895	GPN-loop GTPase 2 partial	custom
90052	5896	39S ribosomal protein L41 mitochondrial partial	custom
NA	5897	Clarin-3 partial	custom
NA	5898	Solute carrier family 25 member 46 partial	custom
NA	5899	Ankyrin repeat and SOCS box protein 13 partial	custom
NA	5903	PREDICTED: DENN domain-containing protein 1A-like	nr
NA	5905	putative complex I intermediate-associated protein 30 mitochondrial partial	custom
90357	5906	Superoxide dismutase [Cu-Zn] partial	custom
NA	5907	AP-2 complex subunit mu partial	custom
NA	5909	hypothetical protein X975 24298 partial	custom
NA	5912	TBC1 domain family member 7 partial	custom
90583	5914	putative 28S ribosomal protein S25 mitochondrial partial	custom
NA	5916	Protein enabled partial	custom

Supplemental Table 3 – continued from previous page

Arthropod Core ID	Spider Core ID	Putative Ortholog Description	Target Database
NA	5917	Ribosome-recycling factor mitochondrial partial	custom
NA	5920	hypothetical protein X975 12776 partial	custom
NA	5925	Sulfotransferase 4A1 partial	custom
NA	5926	CD9 antigen partial	custom
NA	5928	hypothetical protein X975 19454 partial	custom
NA	5931	DnaJ-like protein subfamily C member 1 partial	custom
90423	5932	Ubiquitin fusion degradation protein 1-like protein partial	custom
NA	5936	Mid1-interacting protein 1-B partial	custom
NA	5937	hypothetical protein X975 24825 partial	custom
NA	5938	Low molecular weight phosphotyrosine protein phosphatase partial	custom
90156	5939	hypothetical protein X975 22176 partial	custom
NA	5941	Lambda-crystallin-like protein partial	custom
NA	5944	Neural-cadherin partial	custom
NA	5948	Flotillin-1 partial	custom
NA	5949	NA	nr
89806	5951	Translocon-associated protein subunit gamma partial	custom
NA	5956	Nucleoside diphosphate kinase-like protein partial	custom
NA	5963	hypothetical protein X975 17104 partial	custom
NA	5968	Cell cycle control protein 50A partial	custom
NA	5973	Casein kinase I isoform epsilon partial	custom

Supplemental Table 3 – continued from previous page

Arthropod Core ID	Spider Core ID	Putative Ortholog Description	Target Database
90488	5974	putative ATP-dependent RNA helicase DDX43 partial	custom
NA	5978	hypothetical protein X975 01386 partial	custom
NA	5979	hypothetical protein X975 02502 partial	custom
90509	5980	tRNA (adenine(58)-N(1))-methyltransferase catalytic subunit TRMT61A partial	custom
NA	5986	40S ribosomal protein S19 partial	custom
90283	5987	Dual specificity protein phosphatase 12 partial	custom
NA	5988	Brain-specific angiogenesis inhibitor 1-associated protein 2 partial	custom
NA	5989	Sperm-associated antigen 7-like protein partial	custom
89886	5992	Exosome complex component RRP40 partial	custom
NA	5993	Alpha-12-mannosyltransferase ALG9 partial	custom
NA	5994	ADP-ribosylation factor-like protein 2 partial	custom
NA	5995	DNA-directed RNA polymerase III subunit RPC6 partial	custom
NA	5997	40S ribosomal protein S16 partial	custom
NA	5998	Golgin subfamily A member 3 partial	custom
NA	5999	RNA-binding protein 41 partial	custom
89934	6003	Endothelial differentiation-related factor 1-like protein partial	custom
NA	6006	PAXIP1-associated protein 1 partial	custom

Supplemental Table 3 – continued from previous page

Arthropod Core ID	Spider Core ID	Putative Ortholog Description	Target Database
NA	6008	Cytosolic carboxypeptidase-like protein 5 partial	custom
NA	6011	Synembryn-A partial	custom
90521	6012	Tetratricopeptide repeat protein 26 partial	custom
NA	6016	hypothetical protein X975 13078 partial	custom
NA	6019	hypothetical protein X975 06563 partial	custom
89987	6021	Ubiquitin-like modifier-activating enzyme 5 partial	custom
NA	6023	Dolichyl-phosphate beta-glucosyltransferase partial	custom
NA	6026	Proteasomal ATPase-associated factor 1 partial	custom
NA	6032	putative nuclear transport factor 2 partial	custom
NA	6035	TBC1 domain family member 2B partial	custom
NA	6036	La-related protein 6 partial	custom
90204	6038	Acyl-CoA synthetase family member 2 mitochondrial partial	custom
NA	6045	RNA pseudouridylate synthase domain-containing protein 2 partial	custom
NA	6049	Cyclin-dependent kinase 5 partial	custom
NA	6050	Sushi von Willebrand factor type A EGF and pentraxin domain-containing protein 1 partial	custom
NA	6059	hypothetical protein X975 14693 partial	custom
NA	6066	Lysoplasmalogenase-like protein TMEM86A partial	custom
NA	6070	hypothetical protein X975 08491 partial	custom

Arthropod Core ID	Spider Core ID	Putative Ortholog Description	Target Database
NA	6075	Peptidyl-prolyl cis-trans isomerase FKBP65 partial	custom
NA	6076	Methyltransferase-like protein 5 partial	custom
NA	6077	Poly [ADP-ribose] polymerase 1 partial	custom
NA	6078	MAU2 chromatid cohesion factor-like protein partial	custom
NA	6085	Nuclear transcription factor Y subunit beta partial	custom
NA	6087	WW domain-containing oxidoreductase partial	custom
NA	6091	Phosphatidylinositol transfer protein alpha isoform partial	custom
NA	6092	U3 small nucleolar ribonucleoprotein IMP4 partial	custom
90348	6093	Retinoblastoma-binding protein 5 partial	custom
NA	6094	RING-box protein 2 partial	custom
NA	6095	PREDICTED: transmembrane protein 199-like	nr
90091	6096	Syntaxin-18 partial	custom
NA	6098	Serine proteinase stubble partial	custom
90161	6100	Peptidyl-prolyl cis-trans isomerase CWC27-like protein partial	custom
NA	6102	FGFR1 oncogene partner 2-like protein partial	custom
NA	6103	SNARE-associated protein Snapin partial	custom
NA	6104	26S proteasome non-ATPase regulatory subunit 8 partial	custom
NA	6105	Coiled-coil domain-containing protein 75 partial	custom

Supplemental Table 3 – continued from previous page

Arthropod Core ID	Spider Core ID	Putative Ortholog Description	Target Database
NA	6106	UV radiation resistance-associated protein partial	custom
89739	6107	Dehydrogenase/reductase SDR family member 4 partial	custom
NA	6108	hypothetical protein X975 22088 partial	custom
NA	6109	Gem-associated protein 2 partial	custom
89681	6111	Ribosome biogenesis protein-like protein partial	custom
90187	6112	Transmembrane protein 147 partial	custom
NA	6113	Golgin-45 partial	custom
NA	6120	Serine/threonine-protein kinase Nek7 partial	custom
NA	6125	DNA polymerase eta	nr
NA	6130	Evolutionarily conserved signaling intermediate in Toll pathway mitochondrial partial	custom
NA	6154	NIF3-like protein 1 partial	custom
NA	6155	DNA polymerase delta subunit 2 partial	custom
NA	6158	hypothetical protein X975 14586 partial	custom
NA	6161	Protein patched partial	custom
NA	6162	Embryonic polarity protein dorsal partial	custom
NA	6164	Ubiquitin-conjugating enzyme E2 partial	custom
NA	6165	40S ribosomal protein S15 partial	custom
90408	6167	Ubiquitin-fold modifier-conjugating enzyme 1 partial	custom
NA	6168	Nuclear pore complex protein Nup160 partial	custom

Supplemental Table 3 – continued from previous page

Arthropod Core ID	Spider Core ID	Putative Ortholog Description	Target Database
NA	6169	Serine protease HTRA2 mitochondrial partial	custom
NA	6170	Tyrosine-protein phosphatase corkscrew partial	custom
NA	6171	Exportin-7 partial	custom
NA	6172	putative methyltransferase partial	custom
NA	6173	A-kinase anchor protein 17A partial	custom
NA	6175	Thioredoxin-like protein 4A partial	custom
NA	6176	39S ribosomal protein L11 mitochondrial partial	custom
NA	6181	N-acetyltransferase 6 partial	custom
NA	6190	putative glutamate–tRNA ligase mitochondrial partial	custom
NA	6197	hypothetical protein X975 19445 partial	custom
NA	6199	DNA/RNA-binding protein KIN17 partial	custom
NA	6203	snRNA-activating protein complex subunit 1 partial	custom
NA	6207	Myosin light chain alkali partial	custom
NA	6213	hypothetical protein X975 17964 partial	custom
NA	6215	Farnesyl pyrophosphate synthase partial	custom
NA	6220	Histone deacetylase complex subunit SAP30-like protein partial	custom
NA	6221	hypothetical protein X975 11149 partial	custom
NA	6222	hypothetical protein X975 12775 partial	custom
NA	6223	U3 small nucleolar ribonucleoprotein MPP10 partial	custom
90566	6224	Fatty acid-binding protein partial	custom

Supplemental Table 3 – continued from previous page

Arthropod Core ID	Spider Core ID	Putative Ortholog Description	Target Database
NA	6225	Ribosomal L1 domain-containing protein 1 partial	custom
NA	6226	Rho guanine nucleotide exchange factor 26 partial	custom
NA	6227	Aquaporin-9 partial	custom
NA	6228	hypothetical protein X975 20306 partial	custom
NA	6229	putative succinate dehydrogenase [ubiquinone] cytochrome b small subunit mitochondrial partial	custom
NA	6233	Zinc finger protein 430 partial	custom
NA	6235	hypothetical protein X975 10533 partial	custom
NA	6237	rRNA methyltransferase 1 mitochondrial partial	custom
NA	6238	hypothetical protein X975 02062 partial	custom
NA	6239	hypothetical protein X975 14941 partial	custom
NA	6242	Scavenger mRNA-decapping enzyme DcpS partial	custom
NA	6243	TSC22 domain family protein 1 partial	custom
NA	6244	hypothetical protein X975 21388 partial	custom
NA	6245	RWD domain-containing protein 3 partial	custom
NA	6247	WAS protein family-like protein partial	custom
NA	6248	hypothetical protein X975 04136 partial	custom
NA	6249	Zinc finger C4H2 domain-containing protein partial	custom
90157	6251	60s ribosomal protein L36	nr
NA	6252	Protein FAM89A partial	custom

Supplemental Table 3 – continued from previous page

Arthropod Core ID	Spider Core ID	Putative Ortholog Description	Target Database
NA	6254	40S ribosomal protein S15a partial	custom
NA	6255	Ras GTPase-activating protein SynGAP partial	custom
90450	6260	40S ribosomal protein S23 partial	custom
NA	6261	GTP-binding protein SAR1b partial	custom
NA	6262	40S ribosomal protein S11 partial	custom
NA	6264	Mediator of RNA polymerase II transcription subunit 28 partial	custom
NA	6266	Amiloride-sensitive cation channel 5 partial	custom
NA	6269	PREDICTED: protoporhyrinogen oxidase	nr
NA	6270	hypothetical protein X975 14454 partial	custom
NA	6271	hypothetical protein X975 15250 partial	custom
NA	6272	hypothetical protein X975 13709 partial	custom
NA	6273	Cysteine-rich with EGF-like domain protein 1 partial	custom
NA	6275	U24-ctenitoxin-Pn1a partial	custom
NA	6277	hypothetical protein X975 19973 partial	custom
NA	6278	putative transcription factor SOX-14 partial	custom
NA	6282	Vesicle transport protein USE1 partial	custom
NA	6284	Myosin light chain alkali partial	custom
NA	6286	Protein lethal(2)essential for life partial	custom
NA	6288	Zinc finger protein 385A partial	custom
NA	6290	Platelet glycoprotein Ib alpha chain partial	custom

Supplemental Table 3 – continued from previous page

Arthropod Core ID	Spider Core ID	Putative Ortholog Description	Target Database
89954	6293	Ubiquitin thioesterase OTUB1 partial	custom
NA	6301	WW domain-binding protein 4 partial	custom
NA	6303	Nuclear-interacting partner of ALK partial	custom
NA	6304	Sulfiredoxin-1 partial	custom
NA	6305	Zinc finger CCCH domain-containing protein 3 partial	custom
NA	6309	hypothetical protein X975 05641 partial	custom
NA	6311	hypothetical protein X975 27251 partial	custom
NA	6313	hypothetical protein X975 12832 partial	custom
NA	6327	Importin-5 partial	custom
NA	6334	Zinc finger CCHC-type and RNA-binding motif-containing protein 1 partial	custom
NA	6335	PREDICTED: mediator of RNA polymerase II transcription subunit 31-A-like	nr
NA	6336	Alpha-tocopherol transfer protein-like protein partial	custom
NA	6339	Vacuolar protein sorting-associated protein 13C partial	custom
NA	6341	PREDICTED: RNA-binding protein 7-like	nr
NA	6345	hypothetical protein X975 13533 partial	custom
89764	6348	Vesicle-trafficking protein SEC22b partial	custom
NA	6349	Nucleoporin NUP53 partial	custom
NA	6352	hypothetical protein X975 02024 partial	custom

Supplemental Table 3 – continued from previous page

Arthropod Core ID	Spider Core ID	Putative Ortholog Description	Target Database
NA	6353	putative dolichol-phosphate mannosyltransferase partial	custom
NA	6354	Exosome complex component CSL4 partial	custom
NA	6355	2-methoxy-6-polyprenyl-14-benzoquinol methylase mitochondrial partial	custom
NA	6356	Protein DGCR6 partial	custom
89986	6365	AP-2 complex subunit sigma partial	custom
NA	6367	Mucosa-associated lymphoid tissue lymphoma translocation protein 1-like protein partial	custom
NA	6368	Fibroblast growth factor receptor-like 1 partial	custom
90636	6373	Protein AATF partial	custom
NA	6378	Eukaryotic translation initiation factor 6 partial	custom
NA	6379	GDP-fucose protein O-fucosyltransferase 2 partial	custom
NA	6381	hypothetical protein X975 02449 partial	custom
NA	6395	Dehydrogenase/reductase SDR family member on chromosome X partial	custom
NA	6396	G-protein coupled receptor moody partial	custom
NA	6402	40S ribosomal protein S20 partial	custom
90373	6403	hypothetical protein X975 14484 partial	custom
NA	6404	Glutamate-gated chloride channel partial	custom
NA	6406	NudC domain-containing protein 2 partial	custom

Supplemental Table 3 – continued from previous page

Arthropod Core ID	Spider Core ID	Putative Ortholog Description	Target Database
NA	6407	Motile sperm domain-containing protein 1 partial	custom
NA	6408	Heat shock protein 14 partial	custom
90494	6409	Methyltransferase-like protein 6 partial	custom
NA	6410	PREDICTED: phosphatidylinositol-glycan biosynthesis class W protein-like	nr
NA	6414	DNA primase small subunit partial	custom
NA	6415	tRNA (guanine-N(7)-)-methyltransferase partial	custom
NA	6416	F-box only protein 3 partial	custom
NA	6417	Gamma-tubulin complex component 4 partial	custom
NA	6418	Polyamine-modulated factor 1 partial	custom
NA	6419	Tectonin beta-propeller repeat-containing protein 2 partial	custom
90463	6421	Proteasome subunit beta type-1 partial	custom
NA	6423	Retrograde Golgi transport protein RGP1-like protein partial	custom
NA	6424	Protein Njmu-R1 partial	custom
NA	6425	hypothetical protein X975 26411 partial	custom
NA	6426	DNA damage-regulated autophagy modulator protein 2 partial	custom
NA	6427	Phosphatidylinositol glycan anchor biosynthesis class U protein partial	custom
NA	6428	hypothetical protein X975 14872 partial	custom

Supplemental Table 3 – continued from previous page

Arthropod Core ID	Spider Core ID	Putative Ortholog Description	Target Database
NA	6431	NF-kappa-B-repressing factor partial	custom
NA	6434	hypothetical protein X975 07077 partial	custom
NA	6435	CDGSH iron-sulfur domain-containing protein 3 mitochondrial partial	custom
NA	6437	hypothetical protein X975 10237 partial	custom
NA	6441	NA	nr
NA	6442	28S ribosomal protein S28 mitochondrial partial	custom
NA	6445	Mitochondrial uncoupling protein 4 partial	custom
NA	6447	hypothetical protein X975 00727 partial	custom
NA	6448	Mitochondrial chaperone BCS1 partial	custom
NA	6449	Chondroitin proteoglycan 2 partial	custom
NA	6451	Tat-linked quality control protein TatD partial	custom
NA	6454	hypothetical protein X975 24868 partial	custom
NA	6472	Peptidyl-prolyl cis-trans isomerase-like 4 partial	custom
NA	6473	PREDICTED: serine beta-lactamase-like protain LACTB, mitochondrial	nr
NA	6477	Pyridine nucleotide-disulfide oxidoreductase domain-containing protein 1 partial	custom
NA	6481	hypothetical protein X975 13749 partial	custom
NA	6485	Solute carrier family 10 member 6 partial	custom
NA	6487	Enolase-phosphatase E1 partial	custom
NA	6488	Cyclin-dependent kinase 12 partial	custom

Supplemental Table 3 – continued from previous page

Arthropod Core ID	Spider Core ID	Putative Ortholog Description	Target Database
NA	6491	Torsin-1A-interacting protein 1 partial	custom
NA	6492	Dystrophin-like protein 1 partial	custom
NA	6498	Leucine-rich repeat-containing protein 4B partial	custom
NA	6499	hypothetical protein X975 20564 partial	custom
NA	6503	Ketosamine-3-kinase partial	custom
NA	6504	Nucleoporin GLE1 partial	custom
NA	6505	hypothetical protein X975 25741 partial	custom
90028	6506	putative small nuclear ribonucleoprotein Sm D1 partial	custom
NA	6507	Protein retinal degeneration B partial	custom
90186	6510	Transmembrane protein 208 partial	custom
NA	6512	Protein FAM160B1 partial	custom
NA	6513	SOSS complex subunit B1 partial	custom
NA	6514	Proline-rich protein PRCC partial	custom
90326	6518	Pre-mRNA-splicing factor 38B partial	custom
NA	6519	Mesoderm induction early response protein 3 partial	custom
NA	6523	Protocadherin Fat 1 partial	custom
NA	6524	Phosphatidate phosphatase PPAPDC1B partial	custom
NA	6525	Gamma-secretase subunit PEN-2 partial	custom
NA	6526	ADP-ribosylation factor-like protein 4A partial	custom
NA	6527	GTP-binding protein 5 partial	custom

Supplemental Table 3 – continued from previous page

Arthropod Core ID	Spider Core ID	Putative Ortholog Description	Target Database
89776	6534	PREDICTED: COX assembly mitochondrial protein homolog	nr
90432	6543	Glutamate-rich WD repeat-containing protein 1 partial	custom
NA	6545	Cation transport regulator-like protein 2 partial	custom
NA	6546	hypothetical protein X975 25744 partial	custom
NA	6549	hypothetical protein X975 26200 partial	custom
NA	6551	E3 ubiquitin-protein ligase parkin partial	custom
NA	6553	Beta-14-mannosyl-glycoprotein 4-beta-N-acetylglucosaminyltransferase partial	custom
89795	6554	CAP-Gly domain-containing linker protein 1 partial	custom
NA	6555	Coiled-coil domain-containing protein 130-like protein partial	custom
NA	6556	SPATS2-like protein partial	custom
NA	6558	Transmembrane protein 115 partial	custom
NA	6559	Junction-mediating and -regulatory protein partial	custom
NA	6560	hypothetical protein X975 26046 partial	custom
NA	6561	Dual specificity protein phosphatase 19 partial	custom
90305	6562	Protein mago nashi-like protein partial	custom
NA	6563	Loss of heterozygosity 12 chromosomal region 1 protein-like protein partial	custom
NA	6564	Clotting factor B partial	custom

Supplemental Table 3 – continued from previous page

Arthropod Core ID	Spider Core ID	Putative Ortholog Description	Target Database
NA	6565	Nucleolar protein 14 partial	custom
90114	6566	Histone-binding protein N1/N2 partial	custom
90206	6567	60S ribosomal protein L15 partial	custom
NA	6568	Proteasome assembly chaperone 2 partial	custom
NA	6569	hypothetical protein X975 07522 partial	custom
NA	6570	Unhealthy ribosome biogenesis protein 2-like protein partial	custom
NA	6572	hypothetical protein X975 09911 partial	custom
NA	6575	Condensin-2 complex subunit H2 partial	custom
NA	6576	MIP18 family protein partial	custom
90464	6577	Mitochondrial fission 1 protein partial	custom
NA	6584	3-hydroxyacyl-CoA dehydrogenase type-2 partial	custom
NA	6587	Succinate dehydrogenase cytochrome b560 subunit mitochondrial partial	custom
NA	6588	Malignant T-cell-amplified sequence 1 partial	custom
NA	6589	Coiled-coil domain-containing protein 86 partial	custom
NA	6590	PREDICTED: ATP-dependent (S)-NAD(P)H-hydrate dehydratase-like	nr
NA	6594	NHP2-like protein 1 partial	custom
NA	6596	Transmembrane protein 85 partial	custom
NA	6597	hypothetical protein X975 27140 partial	custom
NA	6603	Signal peptidase complex catalytic subunit SEC11C partial	custom

Supplemental Table 3 – continued from previous page

Arthropod Core ID	Spider Core ID	Putative Ortholog Description	Target Database
89655	6607	28S ribosomal protein S24 mitochondrial partial	custom
NA	6608	hypothetical protein X975 15788 partial	custom
NA	6615	hypothetical protein X975 18876 partial	custom
NA	6616	PREDICTED: ER membrane protein complex subunit 10-like isoform	nr
NA	6617	Eukaryotic translation initiation factor 3 subunit F partial	custom
NA	6623	Manganese-dependent ADP-ribose/CDP-alcohol diphosphatase partial	custom
NA	6624	Chloride channel protein 2 partial	custom
NA	6625	hypothetical protein X975 22119 partial	custom
NA	6630	WD repeat-containing protein 26 partial	custom
NA	6631	Ras guanyl-releasing protein 3 partial	custom
NA	6632	G1/S-specific cyclin-D3 partial	custom
NA	6635	Pyridoxal kinase partial	custom
89881	6636	Phosphoglycolate phosphatase partial	custom
NA	6637	Graves disease carrier protein partial	custom
NA	6642	GTP:AMP phosphotransferase mitochondrial partial	custom
NA	6643	Peptidase inhibitor 16 partial	custom
NA	6644	Ubiquilin-1 partial	custom
NA	6646	Multidrug resistance-associated protein 1 partial	custom
NA	6651	Ras-like protein 2 partial	custom

Supplemental Table 3 – continued from previous page

Arthropod Core ID	Spider Core ID	Putative Ortholog Description	Target Database
89915	6654	Mitochondrial import receptor subunit TOM40-like protein partial	custom
NA	6657	V-type proton ATPase proteolipid subunit partial	custom
NA	6664	Adapter molecule Crk partial	custom
NA	6665	hypothetical protein X975 11149 partial	custom
NA	6666	Filamentous hemagglutinin partial	custom
NA	6667	Mitochondrial import receptor subunit TOM20-like protein partial	custom
NA	6671	Sulfotransferase family cytosolic 1B member 1 partial	custom
NA	6673	Heme-binding protein 1 partial	custom
NA	6674	DNA repair protein complementing XP-C cells-like protein partial	custom
NA	6675	Protein FAM76A partial	custom
NA	6677	Serine/threonine-protein phosphatase 4 regulatory subunit 2 partial	custom
NA	6684	Retinol dehydrogenase 14 partial	custom
NA	6688	Lysosomal-associated transmembrane protein 4A partial	custom
NA	6693	Zinc finger MYND domain-containing protein 19 partial	custom
NA	6698	Activin receptor type-2B partial	custom
NA	6699	Neurogenic locus notch-like protein 2 partial	custom
NA	6700	Adenylate kinase isoenzyme 6 partial	custom

Supplemental Table 3 – continued from previous page

Arthropod Core ID	Spider Core ID	Putative Ortholog Description	Target Database
NA	6701	NADH dehydrogenase [ubiquinone] iron-sulfur protein 3 mitochondrial partial	custom
NA	6703	hypothetical protein X975 06736 partial	custom
NA	6706	Wilms tumor protein 1-interacting protein-like protein partial	custom
NA	6707	Transcription factor MafK partial	custom
NA	6708	NADH dehydrogenase [ubiquinone] 1 beta subcomplex subunit 9 partial	custom
NA	6709	Alpha N-terminal protein methyltransferase 1A-B partial	custom
NA	6712	PREDICTED: vesicle transport protein SEC20-like	nr
NA	6713	Mitochondrial import inner membrane translocase subunit Tim17-B partial	custom
NA	6715	SH3 domain-binding glutamic acid-rich protein-like protein partial	custom
NA	6717	Rac GTPase-activating protein 1 partial	custom
NA	6718	E3 ubiquitin-protein ligase RNF25 partial	custom
NA	6720	putative RNA exonuclease NEF-sp partial	custom
90256	6722	Ras-related protein Rab-11B partial	custom
90490	6727	DNA-directed RNA polymerases I and III subunit RPAC1 partial	custom
NA	6739	Calcitonin receptor	nr
NA	6753	Pre-mRNA-splicing factor 38A partial	custom

Supplemental Table 3 – continued from previous page

Arthropod Core ID	Spider Core ID	Putative Ortholog Description	Target Database
NA	6761	Platelet-activating factor acetylhydrolase IB subunit gamma partial	custom
NA	6764	Survival motor neuron protein partial	custom
90549	6779	PREDICTED: microspherule protein 1-like	nr
NA	6781	Sel1 repeat-containing protein 1-like protein partial	custom
NA	6782	hypothetical protein X975 09721 partial	custom
NA	6786	Kielin/chordin-like protein partial	custom
NA	6788	6-phosphogluconolactonase partial	custom
NA	6792	Abhydrolase domain-containing protein 11 partial	custom
NA	6793	Nuclear pore complex protein Nup85 partial	custom
NA	6800	60S ribosome subunit biogenesis protein NIP7-like protein partial	custom
89763	6804	hypothetical protein X975 18371 partial	custom
NA	6805	Ragulator complex protein LAMTOR1 partial	custom
90232	6806	40S ribosomal protein S24 partial	custom
90013	6807	Cytochrome c oxidase subunit 5A mitochondrial partial	custom
89860	6808	60S ribosomal protein L6 partial	custom
NA	6809	Ras and EF-hand domain-containing protein partial	custom
NA	6810	Actin-related protein 2/3 complex subunit 4 partial	custom

Supplemental Table 3 – continued from previous page

Arthropod Core ID	Spider Core ID	Putative Ortholog Description	Target Database
NA	6811	Decaprenyl-diphosphate synthase subunit 2 partial	custom
NA	6812	Peptidyl-prolyl cis-trans isomerase-like 1 partial	custom
NA	6813	hypothetical protein X975 19754 partial	custom
NA	6816	General transcription factor IIH subunit 3 partial	custom
NA	6817	putative 39S ribosomal protein L23 mitochondrial partial	custom
NA	6818	39S ribosomal protein L13 mitochondrial partial	custom
NA	6819	Nucleolar protein 6 partial	custom
90331	6821	Abhydrolase domain-containing protein partial	custom
NA	6822	Stromal cell-derived factor 2 partial	custom
89855	6823	Serine/threonine-protein phosphatase 4 catalytic subunit partial	custom
NA	6826	RNA-binding protein with serine-rich domain 1-B partial	custom
NA	6827	Sepiapterin reductase partial	custom
NA	6829	U3 small nucleolar RNA-associated protein 14-like protein partial	custom
NA	6831	hypothetical protein X975 11695 partial	custom
89852	6842	Voltage-dependent anion-selective channel protein 2 partial	custom
NA	6843	PREDICTED: axin-1-like	nr
NA	6844	rRNA-processing protein FCF1-like protein partial	custom

Supplemental Table 3 – continued from previous page

Arthropod Core ID	Spider Core ID	Putative Ortholog Description	Target Database
NA	6863	Tumor suppressor p53-binding protein 1 partial	custom
NA	6870	NA	nr
NA	6871	Prefoldin subunit 5 partial	custom
NA	6872	Methyltransferase-like protein 9 partial	custom
90056	6873	putative RNA-binding protein EIF1AD partial	custom
NA	6874	Ribonuclease H1 partial	custom
NA	6875	DNA-directed RNA polymerase III subunit RPC4 partial	custom
NA	6876	Glucocorticoid-induced transcript 1 protein partial	custom
NA	6877	NA	nr
NA	6878	Anaphase-promoting complex subunit 1 partial	custom
NA	6879	HEAT repeat-containing protein 2 partial	custom
NA	6880	DNA-directed RNA polymerase II subunit RPB3 partial	custom
NA	6882	Zinc finger protein 850 partial	custom
NA	6883	Pre-mRNA-splicing factor ISY1-like protein partial	custom
NA	6884	hypothetical protein X975 24266 partial	custom
NA	6885	Iron-sulfur cluster co-chaperone protein HscB mitochondrial partial	custom
NA	6886	Laminin subunit alpha-2 partial	custom
90150	6888	Glyceraldehyde-3-phosphate dehydrogenase partial	custom
NA	6889	60S acidic ribosomal protein P2 partial	custom

Supplemental Table 3 – continued from previous page

Arthropod Core ID	Spider Core ID	Putative Ortholog Description	Target Database
NA	6890	Proteasome subunit beta type-6 partial	custom
NA	6891	Protein translation factor SUI1-like protein partial	custom
NA	6892	hypothetical protein X975 04391 partial	custom
NA	6893	40S ribosomal protein S10b partial	custom
89760	6894	60S ribosomal protein L31 partial	custom
90031	6895	60S ribosomal protein L7 partial	custom
89731	6896	60S ribosomal protein L30 partial	custom
90264	6897	60S ribosomal protein L12 partial	custom
NA	6898	Ras-related protein Rap-1b partial	custom
NA	6899	Ferritin spleen middle subunit partial	custom
90110	6900	Ras-related protein Rab-14 partial	custom
NA	6901	Midasin partial	custom
90214	6902	General transcription factor IIH subunit 1 partial	custom
NA	6904	DDB1- and CUL4-associated factor 5 partial	custom
90237	6905	Myosin-2 essential light chain partial	custom
NA	6906	Cancer-related nucleoside-triphosphatase-like protein partial	custom
NA	6908	Intraflagellar transport protein 57-like protein partial	custom
NA	6909	hypothetical protein X975 12428 partial	custom
NA	6910	GPI ethanolamine phosphate transferase 1 partial	custom
NA	6911	General transcription factor IIH subunit 4 partial	custom

Supplemental Table 3 – continued from previous page

Arthropod Core ID	Spider Core ID	Putative Ortholog Description	Target Database
NA	6919	hypothetical protein X975 17561 partial	custom
NA	6922	Isobutyryl-CoA dehydrogenase mitochondrial partial	custom
NA	6933	Dimethyladenosine transferase 2 mitochondrial partial	custom
NA	6939	39S ribosomal protein L55 mitochondrial partial	custom
89989	6942	Tafazzin partial	custom
NA	6949	Protein SREK1IP1 partial	custom
NA	6951	Ribonuclease P protein subunit p14 partial	custom
NA	6955	Presqualene diphosphate phosphatase partial	custom
NA	6956	hypothetical protein X975 01203 partial	custom
NA	6957	putative tRNA (uracil-O(2)-)-methyltransferase partial	custom
NA	6958	L-cystatin partial	custom
NA	6959	hypothetical protein X975 24714 partial	custom
90526	6960	Hypothetical protein phosphatase 2C T23F11.1 partial	custom
90189	6961	Mediator of RNA polymerase II transcription subunit 7 partial	custom
NA	6962	Protein pangolin isoforms A/H/I partial	custom
NA	6964	Cytochrome b-c1 complex subunit 7 partial	custom
NA	6967	Golgi SNAP receptor complex member 2 partial	custom
NA	6968	Potassium channel subfamily K member 6 partial	custom

Supplemental Table 3 – continued from previous page

Arthropod Core ID	Spider Core ID	Putative Ortholog Description	Target Database
89817	6969	Cytochrome c oxidase subunit 6A1 mitochondrial partial	custom
NA	6976	Leucine-rich repeat and fibronectin type-III domain-containing protein 4 partial	custom
NA	6977	Tetratricopeptide repeat protein 31 partial	custom
NA	6978	putative methyltransferase-like protein 15 partial	custom
NA	6979	Protein toll partial	custom
NA	6986	Nitrogen permease regulator 2-like protein partial	custom
NA	6989	PREDICTED: NHL repeat-containing protein 2	nr
NA	6991	PREDICTED: UDP-xylose and UDP-N-acetylglucosamine transporter	nr
NA	6993	Sugar transporter SWEET1 partial	custom
NA	6999	Peptidyl-prolyl cis-trans isomerase E partial	custom
NA	7007	hypothetical protein X975 12423 partial	custom
NA	7009	Protoheme IX farnesyltransferase mitochondrial partial	custom
NA	7010	Arf-GAP domain and FG repeat-containing protein 2 partial	custom
NA	7015	Protein cappuccino-like protein partial	custom
NA	7016	Soluble guanylate cyclase 88E partial	custom
NA	7017	Chondroitin proteoglycan-2 partial	custom
NA	7019	hypothetical protein X975 14780 partial	custom

Supplemental Table 3 – continued from previous page

Arthropod Core ID	Spider Core ID	Putative Ortholog Description	Target Database
NA	7023	NADH dehydrogenase [ubiquinone] 1 beta subcomplex subunit 11 mitochondrial partial	custom
NA	7024	Epidermal retinol dehydrogenase 2 partial	custom
NA	7027	Kalirin partial	custom
90324	7032	Ubiquinone biosynthesis protein COQ7-like protein partial	custom
NA	7033	Phospholipase A2 partial	custom
NA	7044	Src kinase-associated phosphoprotein 2 partial	custom
NA	7053	Cysteine-rich secretory protein 2 partial	custom
NA	7064	PREDICTED: uncharacterized protein LOC106466561	nr
NA	7073	Partitioning defective 3-like protein partial	custom
NA	7074	DNA-(apurinic or apyrimidinic site) lyase 2 partial	custom
NA	7082	Leucine carboxyl methyltransferase 2 partial	custom
NA	7083	Acylphosphatase-2 partial	custom
NA	7084	G2/mitotic-specific cyclin-B partial	custom
NA	7085	Ubiquitin-related modifier 1-like protein partial	custom
NA	7087	U3 small nucleolar RNA-associated protein 18-like protein partial	custom
NA	7088	Dual specificity protein phosphatase 3 partial	custom
NA	7089	DNA-directed RNA polymerase II subunit RPB9 partial	custom

Supplemental Table 3 – continued from previous page

Arthropod Core ID	Spider Core ID	Putative Ortholog Description	Target Database
NA	7094	39S ribosomal protein L35 mitochondrial partial	custom
NA	7095	NADH dehydrogenase [ubiquinone] 1 subunit C2 partial	custom
NA	7097	Negative elongation factor E partial	custom
NA	7098	Cation-dependent mannose-6-phosphate receptor partial	custom
NA	7100	Pecanex-like protein 3 partial	custom
NA	7102	D-tyrosyl-tRNA(Tyr) deacylase 1 partial	custom
NA	7103	putative polyprenol reductase partial	custom
NA	7112	Ubiquitin-conjugating enzyme E2 J1 partial	custom
NA	7113	Transmembrane protein 19 partial	custom
NA	7122	NA	nr
NA	7128	hypothetical protein X975 14148 partial	custom
NA	7130	hypothetical protein X975 11730 partial	custom
NA	7133	Upstream activation factor subunit spp27 partial	custom
NA	7146	hypothetical protein X975 11405 partial	custom
NA	7150	hypothetical protein X975 14820 partial	custom
NA	7162	39S ribosomal protein L39 mitochondrial partial	custom
NA	7165	Histone H2B.3 partial	custom
90213	7167	Ras-related protein Rab-20 partial	custom
NA	7170	CUE domain-containing protein 1 partial	custom
NA	7173	Nucleoporin SEH1 partial	custom

Supplemental Table 3 – continued from previous page

Arthropod Core ID	Spider Core ID	Putative Ortholog Description	Target Database
NA	7175	Uridine diphosphate glucose pyrophosphatase partial	custom
NA	7176	Solute carrier family 22 member 1 partial	custom
NA	7177	CBP80/20-dependent translation initiation factor partial	custom
NA	7179	Gamma-glutamylaminecyclotransferase partial	custom
NA	7181	Solute carrier family 2 facilitated glucose transporter member 12 partial	custom
NA	7189	Cysteine-rich with EGF-like domain protein 1 partial	custom
NA	7193	Thymidine kinase cytosolic partial	custom
NA	7195	Acyl-CoA-binding domain-containing protein 5 partial	custom
NA	7197	hypothetical protein X975 23170 partial	custom
NA	7198	Melanoma-associated antigen D2 partial	custom
90551	7204	hypothetical protein X975 12812 partial	custom
NA	7206	hypothetical protein X975 23192 partial	custom
NA	7207	Ubiquinone biosynthesis protein COQ9 mitochondrial partial	custom
NA	7214	DTW domain-containing protein 1	nr
NA	7216	Diphosphomevalonate decarboxylase partial	custom
NA	7221	Cyclin-G-associated kinase partial	custom
NA	7226	RNA (guanine-9-)-methyltransferase domain-containing protein 2 partial	custom

Supplemental Table 3 – continued from previous page

Arthropod Core ID	Spider Core ID	Putative Ortholog Description	Target Database
NA	7228	RNA pseudouridylate synthase domain-containing protein 4 partial	custom
NA	7230	Targeting protein for Xklp2 partial	custom
NA	7237	Selenocysteine insertion sequence-binding protein 2-like protein partial	custom
NA	7247	Arf-GAP with SH3 domain ANK repeat and PH domain-containing protein 1 partial	custom
NA	7248	Protein inturned partial	custom
NA	7253	PREDICTED: DNA-directed RNA polymerases I and III subunit RPAC2-like	nr
NA	7257	L-azetidine-2-carboxylic acid acetyltransferase partial	custom
NA	7258	PREDICTED: intraflaellar transport protein 43 homolog A-like	nr
NA	7259	Proteasome assembly chaperone 3 partial	custom
NA	7260	conserved hypothetical protein	nr
NA	7261	Lysyl oxidase-like protein partial	custom
NA	7262	Protein pellino partial	custom
NA	7264	Coilin partial	custom
89866	7268	DNA repair protein RAD51-like protein partial	custom
NA	7273	DNA-directed RNA polymerase III subunit RPC8 partial	custom
NA	7277	hypothetical protein X975 17113 partial	custom
NA	7278	SUN domain-containing protein 1 partial	custom

Supplemental Table 3 – continued from previous page

Arthropod Core ID	Spider Core ID	Putative Ortholog Description	Target Database
NA	7284	hypothetical protein X975 23741 partial	custom
NA	7285	PREDICTED: protein expanded-like	nr
NA	7289	Ceramide glucosyltransferase partial	custom
NA	7292	Peroxisome biogenesis factor 10 partial	custom
NA	7294	Pre-mRNA cleavage complex 2 protein Pcf11 partial	custom
NA	7295	Histone H2A.V partial	custom
NA	7296	NA	nr
NA	7297	hypothetical protein X975 20039 partial	custom
NA	7298	NA	nr
NA	7299	Mitotic spindle-associated MMXD complex subunit MIP18 partial	custom
NA	7301	28S ribosomal protein S10 mitochondrial partial	custom
NA	7302	FtsJ methyltransferase domain-containing protein 1 partial	custom
NA	7303	Tight junction-associated protein 1 partial	custom
NA	7305	CpG-binding protein partial	custom
NA	7306	NA	nr
NA	7309	28S ribosomal protein S6 mitochondrial partial	custom
NA	7322	Zinc finger protein 830 partial	custom
NA	7323	Protein SGT1 partial	custom
NA	7324	hypothetical protein X975 19984 partial	custom
NA	7325	RNA-binding protein 42 partial	custom
NA	7326	Unconventional myosin-XVIIIa partial	custom

Supplemental Table 3 – continued from previous page

Arthropod Core ID	Spider Core ID	Putative Ortholog Description	Target Database
NA	7328	Thioredoxin domain-containing protein partial	custom
NA	7330	Coiled-coil domain-containing protein 85C partial	custom
NA	7331	Protein JTB partial	custom
NA	7332	putative Ras-related protein Rab-33 partial	custom
NA	7334	Protein C10 partial	custom
NA	7335	Zinc finger CCHC domain-containing protein 10 partial	custom
NA	7340	Autism susceptibility 2 protein partial	custom
NA	7342	Protease-associated domain-containing protein 1 partial	custom
90074	7343	Kinesin-like protein KIF21A partial	custom
NA	7344	DNA polymerase beta partial	custom
NA	7345	Protein VAC14-like protein partial	custom
NA	7350	Gem-associated protein 6 partial	custom
NA	7358	Protein TSSC4 partial	custom
NA	7359	THAP domain-containing protein 6 partial	custom
NA	7360	TATA box-binding protein-associated factor RNA polymerase I subunit C partial	custom
NA	7365	Protein arginine N-methyltransferase 1 partial	custom
NA	7366	Serine/threonine-protein kinase 19 partial	custom
NA	7367	hypothetical protein X975 15305 partial	custom
89793	7368	39S ribosomal protein L4 mitochondrial partial	custom

Supplemental Table 3 – continued from previous page

Arthropod Core ID	Spider Core ID	Putative Ortholog Description	Target Database
NA	7371	Coactosin-like protein partial	custom
NA	7372	Protein FAM18B1 partial	custom
NA	7377	BET1-like protein partial	custom
NA	7378	Peptidyl-prolyl cis-trans isomerase NIMA-interacting 1 partial	custom
NA	7379	Polycomb group RING finger protein 3 partial	custom
NA	7380	Protein patched partial	custom
90033	7382	hypothetical protein X975 26265 partial	custom
90356	7385	Dihydroorotate dehydrogenase (quinone) mitochondrial partial	custom
NA	7386	Zinc finger RAD18 domain-containing protein partial	custom
NA	7388	hypothetical protein X975 03076 partial	custom
NA	7392	PREDICTED: uncharacterized protein LOC106465114	nr
NA	7393	UbiA prenyltransferase domain-containing protein 1-like protein partial	custom
NA	7411	Voltage-gated hydrogen channel 1 partial	custom
NA	7413	TM2 domain-containing protein partial	custom
NA	7418	hypothetical protein X975 01283 partial	custom
NA	7427	Delta(35)-Delta(24)-dienoyl-CoA isomerase mitochondrial partial	custom
NA	7435	Golgi pH regulator partial	custom
NA	7436	D-dopachrome decarboxylase-A partial	custom

Supplemental Table 3 – continued from previous page

Arthropod Core ID	Spider Core ID	Putative Ortholog Description	Target Database
NA	7442	Transient receptor hypothetical cation channel subfamily M member 6 partial	custom
NA	7443	Nucleolar protein 12 partial	custom
NA	7444	FERM RhoGEF and pleckstrin domain-containing protein 2 partial	custom
NA	7445	hypothetical protein X975 05858 partial	custom
NA	7447	Metallo-beta-lactamase domain-containing protein 1 partial	custom
89648	7448	60S ribosomal protein L21 partial	custom
NA	7449	L-fucose kinase partial	custom
NA	7450	WD repeat SAM and U-box domain-containing protein 1 partial	custom
NA	7455	hypothetical protein X975 24529 partial	custom
NA	7456	putative dimethyladenosine transferase partial	custom
NA	7458	Hermansky-Pudlak syndrome 1 protein partial	custom
NA	7462	RPA-interacting protein A partial	custom
NA	7464	Hemicentin-1 partial	custom
NA	7466	MRG-binding protein partial	custom
NA	7467	hypothetical protein X975 14363 partial	custom
NA	7469	Sentrin-specific protease 8 partial	custom
NA	7475	Caseinolytic peptidase B protein-like protein partial	custom
NA	7476	Caseinolytic peptidase B protein-like protein partial	custom

Supplemental Table 3 – continued from previous page

Arthropod Core ID	Spider Core ID	Putative Ortholog Description	Target Database
NA	7477	PHD and RING finger domain-containing protein 1 partial	custom
90628	7478	Calcium channel flower partial	custom
NA	7490	NA	nr
90527	7499	Serine/threonine-protein phosphatase 5 partial	custom
NA	7505	cGMP-specific 3'5'-cyclic phosphodiesterase partial	custom
NA	7512	Heme-binding protein 1 partial	custom
NA	7514	hypothetical protein X975 11974 partial	custom
NA	7522	Mpv17-like protein 2 partial	custom
NA	7535	2'5'-phosphodiesterase 12 partial	custom
NA	7541	hypothetical protein X975 23765 partial	custom
NA	7547	Unhealthy ribosome biogenesis protein 2-like protein partial	custom
NA	7549	Transcription initiation factor TFIID subunit 1 partial	custom
NA	7551	putative nuclear hormone receptor HR38 partial	custom
NA	7554	putative lysine-specific demethylase 4B partial	custom
NA	7568	Lysyl oxidase-like protein partial	custom
NA	7572	NADH dehydrogenase [ubiquinone] 1 alpha subcomplex subunit 6 partial	custom
NA	7573	Protein phosphatase 1 regulatory subunit 11 partial	custom
NA	7577	hypothetical protein X975 06657 partial	custom

Supplemental Table 3 – continued from previous page

Arthropod Core ID	Spider Core ID	Putative Ortholog Description	Target Database
NA	7580	hypothetical protein X975 00950 partial	custom
NA	7583	hypothetical protein X975 17375 partial	custom
NA	7590	Cleavage stimulation factor subunit 3 partial	custom
NA	7592	Transmembrane protein 8A partial	custom
90403	7600	Vesicle transport through interaction with t-SNAREs-like protein 1A partial	custom
NA	7604	ADP-ribosylation factor-like protein 6-interacting protein 1 partial	custom
NA	7607	Actin-related protein 2/3 complex subunit 5 partial	custom
NA	7612	Transcription factor Sp9 partial	custom
NA	7623	Protein spaetzle partial	custom
NA	7625	DNA replication complex GINS protein SLD5 partial	custom
89809	7663	Ribosome biogenesis protein BRX1-like protein partial	custom
NA	7664	hypothetical protein X975 24843 partial	custom
NA	7665	GPI-anchor transamidase partial	custom
NA	7666	Ras-related protein Rab-23 partial	custom
NA	7667	Xaa-Pro dipeptidase partial	custom
NA	7669	Protein misato-like protein partial	custom
NA	7670	Ribosomal RNA-processing protein 8 partial	custom
NA	7671	WD repeat and HMG-box DNA-binding protein 1 partial	custom

Supplemental Table 3 – continued from previous page

Arthropod Core ID	Spider Core ID	Putative Ortholog Description	Target Database
NA	7672	YrdC domain-containing protein mitochondrial partial	custom
NA	7673	hypothetical protein X975 10626 partial	custom
NA	7674	NAD-dependent lysine demalonylase and desuccinylase sirtuin-5 mitochondrial partial	custom
NA	7679	Hermansky-Pudlak syndrome 3 protein-like protein partial	custom
NA	7680	Glutamyl aminopeptidase partial	custom
NA	7682	KIF1-binding protein-like protein partial	custom
NA	7683	DNA mismatch repair protein Mlh3 partial	custom
NA	7684	Dephospho-CoA kinase domain-containing protein partial	custom
NA	7685	Importin-13 partial	custom
NA	7686	G protein-coupled receptor kinase 5 partial	custom
NA	7687	Intraflagellar transport protein 81-like protein partial	custom
NA	7689	hypothetical protein X975 18367 partial	custom
NA	7690	Eukaryotic translation initiation factor 4E partial	custom
NA	7692	DNA-directed RNA polymerase II subunit RPB7 partial	custom
NA	7693	Tudor domain-containing protein 5 partial	custom
90239	7694	60S ribosomal protein L19 partial	custom
NA	7695	Rab-like protein 5 partial	custom

Supplemental Table 3 – continued from previous page

Arthropod Core ID	Spider Core ID	Putative Ortholog Description	Target Database
NA	7696	Eukaryotic translation initiation factor 4E-binding protein 1 partial	custom
NA	7697	GATA zinc finger domain-containing protein 1 partial	custom
90430	7698	putative small nuclear ribonucleoprotein Sm D2 partial	custom
89671	7699	60S ribosomal protein L18 partial	custom
90361	7702	PREDICTED: 40S ribosomal protein S8-like	nr
89966	7703	60S ribosomal protein L44 partial	custom
NA	7704	GTP-binding protein 8 partial	custom
NA	7705	Coiled-coil domain-containing protein 58 partial	custom
NA	7706	FRAS1-related extracellular matrix protein 1 partial	custom
NA	7708	Transcription initiation factor IIA subunit 2 partial	custom
NA	7709	U6 snRNA-associated Sm-like protein LSm7 partial	custom
NA	7710	hypothetical protein X975 05234 partial	custom
NA	7711	hypothetical protein X975 07387 partial	custom
NA	7712	hypothetical protein X975 00393 partial	custom
NA	7713	hypothetical protein X975 20400 partial	custom
NA	7714	N-alpha-acetyltransferase 10 partial	custom
NA	7715	hypothetical protein X975 12171 partial	custom
NA	7716	Asparagine synthetase domain-containing protein 1 partial	custom

Supplemental Table 3 – continued from previous page

Arthropod Core ID	Spider Core ID	Putative Ortholog Description	Target Database
90416	7717	Disintegrin and metalloproteinase domain-containing protein 10 partial	custom
NA	7718	hypothetical protein X975 10055 partial	custom
NA	7719	Alanine–tRNA ligase mitochondrial partial	custom
NA	7720	Poly(ADP-ribose) glycohydrolase ARH3 partial	custom
NA	7721	Biogenesis of lysosome-related organelles complex 1 subunit 2 partial	custom
NA	7730	hypothetical protein X975 18919 partial	custom
NA	7742	INO80 complex subunit B partial	custom
NA	7761	Heat shock protein 67B2 partial	custom
NA	7765	Serine/arginine-rich splicing factor 7 partial	custom
NA	7771	DnaJ-like protein subfamily C member 30 partial	custom
NA	7773	28S ribosomal protein S18a mitochondrial partial	custom
NA	7774	Syntaxin-7 partial	custom
NA	7779	putative peptidyl-tRNA hydrolase PTRHD1 partial	custom
NA	7780	28S ribosomal protein S33 mitochondrial partial	custom
89923	7781	Cytochrome c oxidase subunit 5B mitochondrial partial	custom
NA	7782	Receptor expression-enhancing protein 4 partial	custom
NA	7784	hypothetical protein X975 25077 partial	custom
90405	7787	3-hydroxyacyl-CoA dehydratase 1 partial	custom

Supplemental Table 3 – continued from previous page

Arthropod Core ID	Spider Core ID	Putative Ortholog Description	Target Database
NA	7794	Ribonuclease H2 subunit C partial	custom
NA	7799	Protein spaetzle partial	custom
NA	7804	PREDICTED: all-trans retinoic acid-induced differentiation factor-like	nr
NA	7809	hypothetical protein X975 01033 partial	custom
NA	7816	NA	nr
NA	7837	Glutathione S-transferase 1 partial	custom
NA	7845	hypothetical protein X975 07973 partial	custom
NA	7847	Immunoglobulin superfamily containing leucine-rich repeat protein partial	custom
NA	7857	hypothetical protein X975 14061 partial	custom
NA	7860	Thioredoxin-like protein 4B partial	custom
NA	7890	Fibroblast growth factor receptor-like 1 partial	custom
NA	7896	Peroxisomal membrane protein PEX14 partial	custom
NA	7897	Rab GTPase-binding effector protein 1 partial	custom
NA	7899	Trafficking protein particle complex subunit 1 partial	custom
NA	7900	DNA repair protein complementing XP-A cells-like protein partial	custom
NA	7902	Techylectin-5A partial	custom
NA	7903	La-related protein 7 partial	custom
NA	7908	Receptor-binding cancer antigen partial	custom
NA	7921	PREDICTED: UPF0669 protein C6orf120 homolog	nr

Supplemental Table 3 – continued from previous page

Arthropod Core ID	Spider Core ID	Putative Ortholog Description	Target Database
NA	7923	3-phosphoinositide-dependent protein kinase 1 partial	custom
NA	7933	Ras-related protein Rab-27A partial	custom
NA	7953	hypothetical protein X975 19126 partial	custom
NA	7968	PREDICTED: RNA polymerase II elongation factor ELL-like	nr
NA	7969	N-acylneuraminate-9-phosphatase partial	custom
NA	7972	Retinol dehydrogenase 3 partial	custom
NA	7973	hypothetical protein X975 12626 partial	custom
NA	7974	hypothetical protein X975 25143 partial	custom
NA	7975	Ribonuclease P protein subunit p30 partial	custom
NA	7976	PREDICTED: transcription and mRNA export factor ENY2	nr
NA	7977	General transcription factor 3C polypeptide 6 partial	custom
NA	7982	putative nuclease HARBI1 partial	custom
NA	7983	Death domain-associated protein 6 partial	custom
NA	7984	U11/U12 small nuclear ribonucleoprotein partial	custom
NA	7988	Cell division cycle protein 20-like protein partial	custom
NA	7991	Transmembrane protein 222 partial	custom
NA	7997	Alpha-tocopherol transfer protein-like protein partial	custom
NA	8003	Peroxisome assembly protein 12 partial	custom
NA	8008	G-protein coupled receptor Mth2 partial	custom

Supplemental Table 3 – continued from previous page

Arthropod Core ID	Spider Core ID	Putative Ortholog Description	Target Database
NA	8013	Chromodomain Y-like protein partial	custom
89670	8015	PREDICTED: 40S ribosomal protein S12, mitochondrial-like	nr
NA	8020	Protein FAM114A2 partial	custom
NA	8024	hypothetical protein X975 05541 partial	custom
NA	8034	hypothetical protein X975 08854 partial	custom
NA	8037	UDP-GalNAc:beta-13-N-acetylgalactosaminyltransferase 2 partial	custom
NA	8043	tRNA 2'-phosphotransferase 1 partial	custom
NA	8063	hypothetical protein X975 24139 partial	custom
90391	8070	Alanine aminotransferase 2 partial	custom
NA	8073	hypothetical protein X975 20395 partial	custom
NA	8075	Renalase partial	custom
NA	8080	UPF0047 protein yjbQ partial	custom
NA	8083	Exportin-6 partial	custom
NA	8086	Acyl carrier protein mitochondrial partial	custom
NA	8091	Tryptophan–tRNA ligase mitochondrial partial	custom
NA	8094	Glutamate-gated chloride channel partial	custom
NA	8104	39S ribosomal protein L43 mitochondrial partial	custom
NA	8110	hypothetical protein X975 11725 partial	custom
NA	8112	Single-strand selective monofunctional uracil DNA glycosylase partial	custom
NA	8113	Intraflagellar transport protein 27-like protein partial	custom

Supplemental Table 3 – continued from previous page

Arthropod Core ID	Spider Core ID	Putative Ortholog Description	Target Database
89756	8118	Rho GTPase-activating protein 1 partial	custom
NA	8129	Transmembrane channel-like protein 7 partial	custom
NA	8141	Protein farnesyltransferase subunit beta partial	custom
NA	8145	hypothetical protein X975 23521 partial	custom
NA	8149	Ankyrin repeat and SAM domain-containing protein 6 partial	custom
NA	8151	Serine/threonine-protein kinase Nek1 partial	custom
NA	8153	rho gtpase-activating protein 20	nr
NA	8155	Transmembrane protein 185B partial	custom
NA	8157	Cysteine protease ATG4C partial	custom
NA	8158	cAMP-regulated phosphoprotein 21 partial	custom
89941	8159	Mitochondrial intermembrane space import and assembly protein 40-B partial	custom
NA	8161	Selenocysteine-specific elongation factor partial	custom
NA	8164	TM2 domain-containing protein partial	custom
NA	8168	Mitochondrial inner membrane protease ATP23-like protein partial	custom
NA	8178	Laminin subunit alpha-2 partial	custom
NA	8181	PREDICTED: vacuolar protein-sorting-associated protein 36-like	nr
NA	8187	hypothetical protein X975 26977 partial	custom

Supplemental Table 3 – continued from previous page

Arthropod Core ID	Spider Core ID	Putative Ortholog Description	Target Database
NA	8197	hypothetical protein X975 01071 partial	custom
NA	8203	Mitochondrial fission process protein 1 partial	custom
NA	8208	Fatty acid-binding protein partial	custom
NA	8216	hypothetical protein X975 09663 partial	custom
NA	8222	Cytoplasmic tRNA 2-thiolation protein 2 B partial	custom
NA	8226	tRNA-splicing endonuclease subunit Sen2 partial	custom
NA	8227	Serine–tRNA ligase partial	custom
NA	8237	GTP-binding protein SAR1b partial	custom
NA	8239	ADP-ribosylation factor 1 partial	custom
NA	8269	Zinc finger protein 36 C3H1 type-like 1 partial	custom
NA	8281	Protein NDNF partial	custom
NA	8290	Methyltransferase-like protein 21D partial	custom
NA	8296	Transmembrane protein 63B partial	custom
NA	8302	RNA pseudouridylate synthase domain-containing protein 1 partial	custom
NA	8307	Intraflagellar transport protein 172-like protein partial	custom
NA	8311	Acidic leucine-rich nuclear phosphoprotein 32 family member A partial	custom
NA	8312	HEAT repeat-containing protein 1 partial	custom
NA	8313	Sulfotransferase family cytosolic 1B member 1 partial	custom

Supplemental Table 3 – continued from previous page

Arthropod Core ID	Spider Core ID	Putative Ortholog Description	Target Database
NA	8318	hypothetical protein X975 22437 partial	custom
NA	8319	Transmembrane protein 70-like protein mitochondrial partial	custom
NA	8325	hypothetical protein X975 24386 partial	custom
NA	8331	39S ribosomal protein L10 mitochondrial partial	custom
NA	8334	hypothetical protein X975 03325 partial	custom
NA	8335	Pyruvate dehydrogenase protein X component partial	custom
NA	8340	Pleckstrin domain-containing family M member 3 partial	custom
NA	8344	hypothetical protein X975 24110 partial	custom
90025	8371	Rap guanine nucleotide exchange factor 1 partial	custom
NA	8376	Protein UBASH3A-like protein partial	custom
NA	8387	Glycosyltransferase-like protein LARGE2 partial	custom
90547	8392	CAD protein partial	custom
NA	8408	tRNA (cytosine-5-)-methyltransferase partial	custom
NA	8410	Protein N-terminal glutamine amidohydrolase partial	custom
NA	8411	Fibronectin type-III domain-containing protein 3A partial	custom
NA	8429	Uracil-DNA glycosylase partial	custom
NA	8432	hypothetical protein X975 21317 partial	custom
NA	8436	Nuclear speckle splicing regulatory protein 1 partial	custom
NA	8437	PREDICTED: DNA damage-binding protein 2-like isoform	nr

Supplemental Table 3 – continued from previous page

Arthropod Core ID	Spider Core ID	Putative Ortholog Description	Target Database
NA	8438	hypothetical protein X975 18516 partial	custom
NA	8440	Mannose-1-phosphate guanyl-transferase beta-A partial	custom
NA	8441	Target of rapamycin complex 2 subunit MAPKAP1 partial	custom
NA	8446	Transmembrane and ubiquitin-like domain-containing protein 1 partial	custom
NA	8449	2-aminoethanethiol dioxygenase partial	custom
NA	8450	Transcription elongation factor SPT4 partial	custom
NA	8455	Diacylglycerol O-acyltransferase 1 partial	custom
NA	8460	Exosome complex component MTR3 partial	custom
NA	8468	COMM domain-containing protein 4 partial	custom
NA	8473	hypothetical protein X975 15998 partial	custom
NA	8497	Chondroitin proteoglycan-2 partial	custom
NA	8512	hypothetical protein X975 19816 partial	custom
NA	8518	FIT family protein partial	custom
NA	8519	Epithelial splicing regulatory protein 2 partial	custom
NA	8566	Valine–tRNA ligase partial	custom
90210	8569	Surfeit locus protein 4-like protein partial	custom
NA	8570	hypothetical protein X975 12389 partial	custom
NA	8571	hypothetical protein X975 20990 partial	custom

Supplemental Table 3 – continued from previous page

Arthropod Core ID	Spider Core ID	Putative Ortholog Description	Target Database
89903	8573	Transcription initiation factor TFIID subunit 13 partial	custom
NA	8574	Rho guanine nucleotide exchange factor 17 partial	custom
NA	8575	hypothetical protein X975 26350 partial	custom
NA	8576	E3 SUMO-protein ligase PIAS2 partial	custom
NA	8577	Tail-anchored protein insertion receptor WRB partial	custom
90159	8579	Sodium/glucose cotransporter 1 partial	custom
NA	8580	TGF-beta-activated kinase 1 and MAP3K7-binding protein 1 partial	custom
NA	8581	Nephrocystin-1 partial	custom
NA	8584	Gamma-tubulin complex component 2 partial	custom
NA	8586	DNA polymerase delta subunit 3 partial	custom
NA	8590	Ankyrin repeat and MYND domain-containing protein 2 partial	custom
NA	8592	Formimidoyltransferase-cyclodeaminase partial	custom
NA	8593	Golgin subfamily A member 3 partial	custom
NA	8594	putative palmitoyltransferase ZDHHC6 partial	custom
NA	8597	Mitochondrial inner membrane protease subunit 1 partial	custom
NA	8598	Alkylated DNA repair protein alkB-like protein partial	custom
NA	8599	hypothetical protein X975 17379 partial	custom
NA	8606	conserved hypothetical protein	nr

Supplemental Table 3 – continued from previous page

Arthropod Core ID	Spider Core ID	Putative Ortholog Description	Target Database
NA	8607	Nephrin partial	custom
NA	8610	NADH dehydrogenase [ubiquinone] iron-sulfur protein 6 mitochondrial partial	custom
NA	8611	60S ribosomal protein L34 partial	custom
NA	8613	Protein MTO1-like protein mitochondrial partial	custom
NA	8614	Ras-like GTP-binding protein rhoA partial	custom
NA	8615	Acyl-CoA-binding domain-containing protein 5-B partial	custom
NA	8616	Craniofacial development protein 1 partial	custom
NA	8617	Nesprin-2 partial	custom
NA	8618	Mitochondrial inner membrane protease subunit 2 partial	custom
89757	8619	hypothetical protein X975 14513 partial	custom
NA	8621	D-beta-hydroxybutyrate dehydrogenase mitochondrial partial	custom
NA	8623	hypothetical protein X975 00429 partial	custom
NA	8625	Methylmalonic aciduria type A-like protein mitochondrial partial	custom
NA	8626	Ras association domain-containing protein 1 partial	custom
NA	8627	Protein FRA10AC1-like protein partial	custom
NA	8628	p53-induced protein with a death partial	custom
NA	8631	PREDICTED: CDK-activating kinase assembly factor MAT1-like	nr
NA	8632	putative tyrosine–tRNA ligase mitochondrial partial	custom

Supplemental Table 3 – continued from previous page

Arthropod Core ID	Spider Core ID	Putative Ortholog Description	Target Database
NA	8633	Histone acetyltransferase KAT2B partial	custom
NA	8635	PREDICTED: ADP-ribosylation factor-like protein 2-binding protein isoform	nr
NA	8636	CaiB/baiF CoA-transferase family protein partial	custom
NA	8638	WD repeat-containing protein 92 partial	custom
NA	8667	hypothetical protein X975 08855 partial	custom
NA	8719	hypothetical protein X975 01092 partial	custom
NA	8728	Transmembrane protein 14C partial	custom
NA	8731	Heterogeneous nuclear ribonucleoprotein 27C partial	custom
NA	8733	Zinc finger CCCH domain-containing protein 11A partial	custom
NA	8735	2'5'-phosphodiesterase 12 partial	custom
NA	8818	Papilin partial	custom
NA	8880	PREDICTED: protein FAM206A	nr
NA	8882	Protein spaetzle partial	custom
NA	8912	P3 protein partial	custom
NA	8956	Ubiquitin-conjugating enzyme E2 partial	custom
NA	8957	Glutaredoxin-2 mitochondrial partial	custom
NA	8961	hypothetical protein X975 22064 partial	custom
NA	8979	Tryptase partial	custom
NA	8980	Chitotriosidase-1 partial	custom
NA	8982	GA-binding protein alpha chain partial	custom

Supplemental Table 3 – continued from previous page

Arthropod Core ID	Spider Core ID	Putative Ortholog Description	Target Database
NA	9039	hypothetical protein X975 10811 partial	custom
NA	9045	putative RNA polymerase II nuclear localization protein SLC7A6OS partial	custom
NA	9047	Zinc finger matrin-type protein 5 partial	custom
NA	9057	PREDICTED: cyclin-dependent kinase 2-interacting protein	nr
NA	9060	Transmembrane protein 120-like protein partial	custom
NA	9072	Protein BTG2 partial	custom
NA	9073	Fanconi-associated nuclease 1 partial	custom
NA	9077	NA	nr
NA	9081	Required for meiotic nuclear division protein 1-like protein partial	custom
NA	9109	GTP-binding protein Rit1 partial	custom
NA	9118	Peptidoglycan-recognition protein SC2 partial	custom
NA	9120	Beta-14-N-acetylgalactosaminyltransferase bre-4 partial	custom
NA	9124	Globin partial	custom
NA	9132	PREDICTED: myosin heavy chain, muscle-like	nr
NA	9143	hypothetical protein X975 18827 partial	custom
NA	9183	hypothetical protein X975 19496 partial	custom
NA	9188	putative ribosome-binding factor A mitochondrial partial	custom
NA	9202	Secernin-2 partial	custom
NA	9203	Innexin inx2 partial	custom
NA	9212	hypothetical protein X975 19700 partial	custom
NA	9234	AMME syndrome candidate 1 protein-like protein partial	custom

Supplemental Table 3 – continued from previous page

Arthropod Core ID	Spider Core ID	Putative Ortholog Description	Target Database
NA	9249	Tyrosine-protein phosphatase Lar partial	custom
NA	9283	Histone-lysine N-methyltransferase SUV420H1-A partial	custom
NA	9298	Transposable element Hobo transposase partial	custom
NA	9309	Round spermatid basic protein 1-like protein partial	custom
NA	9318	tRNA-specific adenosine deaminase-like protein 3 partial	custom
NA	9326	ADP-ribosylation factor 4 partial	custom
NA	9352	Cytosolic endo-beta-N-acetylglucosaminidase partial	custom
NA	9357	Activating signal cointegrator 1 complex subunit 3 partial	custom
NA	9358	hypothetical protein X975 17122 partial	custom
90579	9359	G2/mitotic-specific cyclin-B3 partial	custom
NA	9405	DNA repair protein XRCC4 partial	custom
NA	9435	DNA-directed RNA polymerase I subunit RPA49 partial	custom
NA	9449	Ribonuclease P protein subunit p20 partial	custom
NA	9455	Protein BTG1 partial	custom
NA	9466	putative ATP-dependent RNA helicase DDX4 partial	custom
NA	9486	Leucine-rich repeat-containing protein partial	custom
NA	9496	Lysosomal Pro-X carboxypeptidase partial	custom
NA	9522	NA	nr

Supplemental Table 3 – continued from previous page

Arthropod Core ID	Spider Core ID	Putative Ortholog Description	Target Database
NA	9533	Ataxin-10 partial	custom
NA	9618	FERM and PDZ domain-containing protein 4 partial	custom
NA	9619	Cob(I)yrinic acid ac-diamide adeno-syltransferase mitochondrial partial	custom
NA	9621	Poly(A) RNA poly-merase mitochondrial partial	custom
NA	9622	Tectonic-3 partial	custom
NA	9627	Ninjurin-1 partial	custom
NA	9629	Ubiquitin carboxyl-terminal hydrolase 3 partial	custom
NA	9632	F-box only protein 28 partial	custom
90201	9637	PREDICTED: PHD finger-like domain-containing protein 5A	nr
NA	9643	Synaptophysin partial	custom
NA	9644	RecQ-mediated genome instability protein 1 partial	custom
NA	9645	Trafficking protein particle complex subunit 2 partial	custom
NA	9647	Repulsive guidance molecule A partial	custom
NA	9648	Ethanolaminephosphotransferase 1 partial	custom
NA	9652	A disintegrin and metalloproteinase with thrombospondin motifs 7 partial	custom
NA	9653	F-box only protein 22 partial	custom
NA	9654	Peroxisomal mem-brane protein 11B partial	custom
NA	9656	hypothetical protein X975 26154 partial	custom
NA	9658	RNA-binding protein 26 partial	custom
NA	9662	hypothetical protein X975 22098 partial	custom

Supplemental Table 3 – continued from previous page

Arthropod Core ID	Spider Core ID	Putative Ortholog Description	Target Database
NA	9664	Rhomboid-related protein 3 partial	custom
NA	9665	Disintegrin and metalloproteinase domain-containing protein 23 partial	custom
NA	9674	Trifunctional nucleotide phosphoesterase protein YfkN partial	custom
NA	9680	hypothetical protein X975 15151 partial	custom
NA	9685	HRAS-like suppressor partial	custom
NA	9690	WD repeat domain-containing protein 83 partial	custom
NA	9691	Serine/threonine-protein kinase LMTK1 partial	custom
89998	9693	Protein jagunal partial	custom
NA	9695	DNA (cytosine-5)-methyltransferase PliMCI partial	custom
NA	9696	Acylphosphatase-2 partial	custom
NA	9697	39S ribosomal protein L14 mitochondrial partial	custom
NA	9699	Integrator complex subunit 5 partial	custom
89889	9700	Helicase SKI2W partial	custom
NA	9702	Uroporphyrinogen decarboxylase partial	custom
NA	9703	ATP synthase lipid-binding protein mitochondrial partial	custom
NA	9707	39S ribosomal protein L21 mitochondrial partial	custom
NA	9709	putative arginine–tRNA ligase mitochondrial partial	custom
NA	9713	Myosin regulatory light chain 2 partial	custom

Supplemental Table 3 – continued from previous page

Arthropod Core ID	Spider Core ID	Putative Ortholog Description	Target Database
NA	9719	Protein lin-54-like protein partial	custom
NA	9720	Trafficking protein particle complex subunit 5 partial	custom
NA	9721	Calmodulin-like protein 4 partial	custom
NA	9787	NA	nr
NA	9852	Integrin alpha-PS1 partial	custom
NA	10030	STAM-binding protein-like A partial	custom
NA	10236	Telomere-associated protein RIF1 partial	custom
NA	10568	DNA-directed RNA polymerase mitochondrial partial	custom
NA	10569	Kalirin partial	custom
NA	10628	Three prime repair exonuclease 2 partial	custom
NA	10673	Germ cell-less protein-like 1 partial	custom
NA	11025	Calcium/calmodulin-dependent protein kinase type 1 partial	custom
NA	11028	28S ribosomal protein S18b mitochondrial partial	custom
NA	11033	Transmembrane protein 173 partial	custom
NA	11034	hypothetical protein X975 22111 partial	custom
NA	11035	hypothetical protein X975 20920 partial	custom
NA	11036	PREDICTED: uncharacterized protein LOC106462232	nr
NA	11038	hypothetical protein X975 19857 partial	custom
NA	11039	hypothetical protein X975 20897 partial	custom
NA	11042	Heat shock protein beta-11 partial	custom
NA	11046	PREDICTED: uncharacterized protein LOC106468561	nr

Supplemental Table 3 – continued from previous page

Arthropod Core ID	Spider Core ID	Putative Ortholog Description	Target Database
NA	11048	Dermal papilla-derived protein 6-like protein partial	custom
NA	11052	CCAAT/enhancer-binding protein gamma partial	custom
NA	11053	NA	nr
NA	11056	CTD small phosphatase-like protein 2 partial	custom
NA	11058	Chaperone protein DnaJ partial	custom
NA	11059	CAP-Gly domain-containing linker protein 4 partial	custom
NA	11060	Peptidyl-prolyl cis-trans isomerase FKBP6 partial	custom
NA	11061	Glycolipid transfer protein domain-containing protein 1 partial	custom
NA	11062	N-acyl-phosphatidylethanolamine-hydrolyzing phospholipase D partial	custom
NA	11063	Serine–tRNA ligase mitochondrial partial	custom
NA	11067	hypothetical protein X975 07422 partial	custom
NA	11069	Toll-interacting protein partial	custom
NA	11072	Oxysterol-binding protein-related protein 11 partial	custom
NA	11075	hypothetical protein X975 14457 partial	custom
NA	11077	Zinc finger BED domain-containing protein 4 partial	custom
NA	11080	Ras-related C3 botulinum toxin substrate 1 partial	custom
NA	11082	Vacuolar protein-sorting-associated protein 25 partial	custom
NA	11085	hypothetical protein X975 14012 partial	custom

Supplemental Table 3 – continued from previous page

Arthropod Core ID	Spider Core ID	Putative Ortholog Description	Target Database
NA	11086	Werner syndrome ATP-dependent helicase partial	custom
NA	11092	hypothetical protein X975 06161 partial	custom
NA	11094	putative E3 ubiquitin-protein ligase MY-CBP2 partial	custom
NA	11095	Monocarboxylate transporter 12 partial	custom
NA	11098	Zinc finger protein 511 partial	custom
NA	11099	PREDICTED: anaphase-promoting complex subunit 4-like	nr
NA	11102	hypothetical protein X975 19798 partial	custom
NA	11111	hypothetical protein X975 13625 partial	custom
NA	11112	hypothetical protein X975 13639 partial	custom
NA	11117	Zonadhesin partial	custom
90435	11126	Mannose-P-dolichol utilization defect 1 protein-like protein partial	custom
NA	11127	Small nuclear ribonucleoprotein-associated protein B partial	custom
NA	11136	putative 60S ribosomal protein L37-A partial	custom
89706	11139	Ras-related protein Rab-39B partial	custom
NA	11140	RNA-binding protein 48	nr
90501	11141	Ras-related protein Rap-1b partial	custom
NA	11142	E3 ubiquitin-protein ligase SIAH1 partial	custom
NA	11143	USP6 N-terminal-like protein partial	custom
NA	11144	Heterogeneous nuclear ribonucleoprotein F partial	custom

Supplemental Table 3 – continued from previous page

Arthropod Core ID	Spider Core ID	Putative Ortholog Description	Target Database
NA	11147	hypothetical protein X975 06894 partial	custom
NA	11148	putative exonuclease mut-7-like protein partial	custom
NA	11149	Tensin-1 partial	custom
NA	11155	hypothetical protein X975 05048 partial	custom
NA	11156	Cell cycle checkpoint protein RAD1 partial	custom
NA	11157	Protein DEK partial	custom
NA	11159	hypothetical protein X975 19611 partial	custom
NA	11169	Transcription initiation protein SPT3-like protein partial	custom
NA	11177	Regulator of microtubule dynamics protein 2 partial	custom
NA	11178	Kinetochore protein NDC80-like protein partial	custom
NA	11180	Poly [ADP-ribose] polymerase 1 partial	custom
NA	11181	conserved hypothetical protein	nr
NA	11183	putative asparagine–tRNA ligase mitochondrial partial	custom
